# Genomic and transcriptomic insights into the thermo-regulated biosynthesis of validamycin in *Streptomyces hygroscopicus* 5008

**DOI:** 10.1186/1471-2164-13-337

**Published:** 2012-07-24

**Authors:** Hang Wu, Shuang Qu, Chenyang Lu, Huajun Zheng, Xiufen Zhou, Linquan Bai, Zixin Deng

**Affiliations:** 1 State Key Laboratory of Microbial Metabolism, and School of Life Sciences & Biotechnology, Shanghai Jiao Tong University, Shanghai, 200030, China; 2 Shanghai-MOST Key Laboratory of Disease and Health Genomics, Chinese National Human Genome Center at Shanghai, Shanghai, 201203, China

**Keywords:** Validamycin, Genome, Transcriptome, *Streptomyces*, Metabolism, Thermo-regulation

## Abstract

**Background:**

*Streptomyces hygroscopicus* 5008 has been used for the production of the antifungal validamycin/jinggangmycin for more than 40 years. A high yield of validamycin is achieved by culturing the strain at 37°C, rather than at 30°C for normal growth and sporulation. The mechanism(s) of its thermo-regulated biosynthesis was largely unknown.

**Results:**

The 10,383,684-bp genome of strain 5008 was completely sequenced and composed of a linear chromosome, a 164.57-kb linear plasmid, and a 73.28-kb circular plasmid. Compared with other *Streptomyces* genomes, the chromosome of strain 5008 has a smaller core region and shorter terminal inverted repeats, encodes more α/β hydrolases, major facilitator superfamily transporters, and Mg^2+^/Mn^2+^-dependent regulatory phosphatases. Transcriptomic analysis revealed that the expression of 7.5% of coding sequences was increased at 37°C, including biosynthetic genes for validamycin and other three secondary metabolites. At 37°C, a glutamate dehydrogenase was transcriptionally up-regulated, and further proved its involvement in validamycin production by gene replacement. Moreover, efficient synthesis and utilization of intracellular glutamate were noticed in strain 5008 at 37°C, revealing glutamate as the nitrogen source for validamycin biosynthesis. Furthermore, a SARP-family regulatory gene with enhanced transcription at 37°C was identified and confirmed to be positively involved in the thermo-regulation of validamycin production by gene inactivation and transcriptional analysis.

**Conclusions:**

Strain 5008 seemed to have evolved with specific genomic components to facilitate the thermo-regulated validamycin biosynthesis. The data obtained here will facilitate future studies for validamycin yield improvement and industrial bioprocess optimization.

## Background

Validamycin A (also named as jinggangmycin A, abbreviated as VAL-A/JIN-A), a basic C_7_N aminocyclitol-containing antibiotic, has been widely used as an antifungal agent against rice sheath blight disease in China and other Eastern Asian countries [1]. Meanwhile, its transformed product valienamine is a pharmaceutically important precursor for the synthesis of voglibose, a highly effective drug for insulin-independent diabetes [2]. VAL-A is produced at large scale in China by the derivatives of *Streptomyces hygroscopicus* var. *jinggangensis* 5008 (hereafter abbreviated as *S. hygroscopicus* 5008 or strain 5008) for more than 40 years. In other countries including Japan and Korea, VAL-A is produced by *S. hygroscopicus* var. *limoneus*[3]. Moreover, a relatively higher fermentation temperature (37°C) for strain 5008, rather than 30°C for normal *Streptomyces* growth and sporulation, rendered remarkably increased VAL-A yield, which stands for an unusual example of positive thermo-regulation on antibiotic production [4,5].

Earlier interests and intensive investigation into the primary metabolism, genetic manipulation, bio-catalysis, and biotransformation of the VAL-A producers were greatly stimulated by its agro-medical importance [6-8]. Feeding experiment with isotope-labeled compounds had established that the sedoheptulose 7-phosphate from the pentose phosphate pathway and d-glucose are the precursors for VAL-A biosynthesis [9,10]. Furthermore, feeding with a series of ^15^ N-labeled precursors showed that glutamate is the likely primary source of the bridge nitrogen [11,12]. Recently, the complete VAL-A biosynthetic gene clusters were independently cloned from strain 5008, *S. hygroscopicus* var. *yingchengensis* 10*–*22, and *S. hygroscopicus* var. *limoneus*[13-16]. Subsequent genetic and biochemical analysis of 10 VAL-A biosynthetic genes confirmed that the sedoheptulose 7-phosphate and UDP-glucose are indeed the precursors and cofactors Co^2+^, Mg^2+^/Mn^2+^, Zn^2+^, GTP/ATP, and NAD^+^ are essentially required for VAL-A biosynthesis [17-22]. Moreover, a correlation between enhanced VAL-A productivity and an increased transcription of biosynthetic genes was established in strain 5008 at 37°C instead of 30°C [4,5]. Functional studies of the VAL-A biosynthetic genes at genetic and biochemical levels have also enabled much improved VAL-A productivity, e.g. through feeding proper amount of Co^2+^, duplicating biosynthetic gene set, or enhancing UDP-glucose biosynthesis [23]. However, the mechanism of the thermo-regulation of VAL-A production by strain 5008 remains largely unknown.

To date, the whole genomes of several antibiotic-producing actinomycetes have been sequenced, including the avermectin producer *S. avermitilis*, the erythromycin producer *Saccharopolyspora erythraea*, the streptomycin producer *S. griseus*, and the rifamycin producer *Amycolatopsis mediterranei*[24-27]. Since the advent of transcriptomics, proteomics and metabolomics, the genome-based functional studies of antibiotic producers shed new lights on antibiotic biosynthesis, development, regulation, phylogeny and evolution, and mining of the rich repertoire of secondary metabolites [28,29]. In this study, we completely sequenced the genome of *S. hygroscopicus* 5008 and analyzed its transcriptomes by cultivating the strain at 30°C or 37°C. A Streptomyces Antibiotic Regulatory Protein (SARP)-family regulator was identified to be involved in a positive control of the thermo-regulated VAL-A biosynthesis by strain 5008.

## Results

### General features of the *S. hygroscopicus* 5008 genome

Except for a linear chromosome, the strain 5008 also harbors a linear plasmid pSHJG1 and a 73,282-bp large circular plasmid (Additional file 1: Figure S1). In order to clone both ends of the linear chromosome and plasmid pSHJG1, we searched for a putative helicase gene homologous to *ttrA*, which is usually located in the termini of actinomycetal chromosomes and linear plasmids [30]. Four putative telomere regions were identified by Southern blotting with *ttrA* probe, cloned, and sequenced [30]. With a total length of 10,383,684 bp, the genome of strain 5008 is larger than most published *Streptomyces* genomes (Table 1).

**Table 1:** General features of seven completely sequenced* Streptomyces *chromosomes *

**Species**	**Length (bp)**	**TIR (bp)**	**GC Content (%)**	**CDS (no.)**	**Average CDS size (bp)**	**Coding (%)**	**rRNA Operons (no.)**	**tRNA (no.)**
*S. hygroscopicus* 5008	10,145,833	14	71.9	8,849	952	83.2	6	68
*S. coelicolor* A3(2)	8,667,507	21,653	72.1	7,825	991	88.9	6	63
*S. avermitilis* MA-4680	9,025,608	49	70.7	7,582	1,027	86.3	6	68
*S. griseus* IFO13350	8,545,929	132,910	72.2	7,138	1,055	88.1	6	66
*S. scabies* 87.22	10,148,695	18,488	71.5	8,746	1,005	86.2	6	75
*S. bingchenggensis* BCW-1	11,936,683	400,000	70.8	10,023	1,031	86.6	6	ND
*S. clavuligerus* ATCC 27064	6,760,392	ND	72.0	5,710	1,031	87.1	6	66

* Data were obtained from GenBank: *S. coelicolor* A3(2), NC_003888; *S. avermitilis* MA-4680, NC_003155; *S. griseus* IFO13350, NC_010572; *S. scabies* 87.22, NC_013929; *S. bingchenggensis* BCW-1, CP002047; *S. clavuligerus* ATCC 27064, CM000913. ND, not determined.

**Figure 1:**
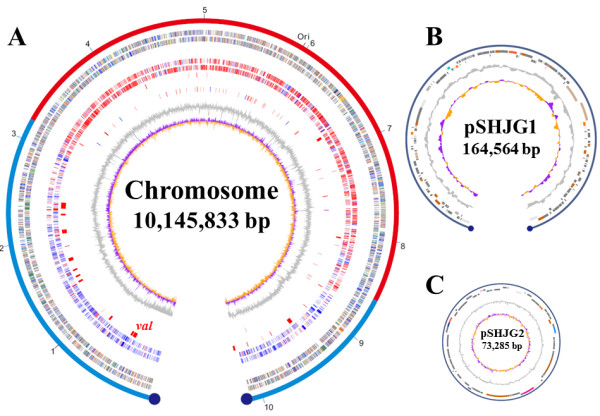
**Schematic representation of the *S. hygroscopicus* 5008 chromosome and two plasmids.** (**A**) The chromosome atlas. The outer scale is numbered in megabases from the left to the right ends and indicates the core (red) and noncore (blue) chromosomal regions; Circle 1 and 2 (forward and reverse strands), predicted protein coding sequences colored according to COG function categories; Circle 3 and 4 (forward and reverse strands), distribution of conserved (red) or strain-specific genes (blue) in 5008 compared with other *Streptomyces* chromosomes; Circle 5, distribution of secondary metabolic gene clusters (red); Circle 6, distribution of tRNA (red) and rRNA operon (blue); Circle 7, GC content; Circle 8, GC bias. Ori, origin of replication. *val*, validamycin biosynthetic gene cluster. (**B**) Atlas of linear plasmid pSHJG1. Circle 1 and 2, predicted coding sequences on the plus and minus strands, respectively, colored according to COG functional categories; Circle 3, GC content; Circle 4, GC bias. (**C**) Atlas of circular plasmid pSHJG2. Circle 1 and 2, predicted coding sequences on the plus and minus strands, respectively, colored according to COG functional categories; Circle 3, GC content; Circle 4, GC bias.

The linear chromosome (10,145,833 bp) of strain 5008, with an average G + C% mol content of 71.9%, comprises 8,849 predicted protein-coding sequences (locus tagged as SHJG), 6 rRNA operons (16 S-23 S-5 S), and 68 tRNA genes (Table 1). The replication origin *oriC* contains at least 18 DnaA box-like sequences [31] and is shifted 875 kb away from the center to the right (Figure 1A). Intriguingly, it only has 14-bp terminal inverted repeats (TIRs), which is one of the shortest TIRs hitherto found in actinomycetes. Based on a BLASTCLUST analysis, 4,607 (41.6%) of predicted protein coding sequences (CDSs) are clustered into 924 families.

The linear plasmid pSHJG1 (164,566 bp) (Figure 1B) contains 184 CDSs possibly involved in replication, partitioning, transfer and other biological functions. It lacks a conserved telomere-associated protein (Tap) and TIRs. However, the rightmost 1.2-kb region of pSHJG1 demonstrates a strong homology to the right arm of the chromosome, implying an evolutionary recombination event occurred between the linear plasmid and the chromosome. Moreover, the left terminus of pSHJG1 is equipped with atypical nucleotide sequences consisting of several packed palindromes with non-conserved loop sequences, thereby forming a different secondary structure from its right end and both chromosome ends (Additional file 2: Figure S2). Intriguingly, a complete bacterial immune system CRISPR-Cas [32] was identified in pSHJG1, suggesting a resistance to phages and other invading genetic elements by strain 5008.

Genome-wide comparison among completely sequenced *Streptomyces* chromosomes revealed highly conserved core regions ranging from 5.50 to 7.25 Mb [SCLAV0503-SCLAV5245 (5.50 Mb), SCO1209-SCO6774 (6.25 Mb), SGR0954-SGR6311 (6.36 Mb), SAV1638-SAV7128 (6.48 Mb), SBI25785-SBI889 (7.12 Mb), SCAB12831-SCAB78641 (7.25 Mb)], substantially in proportion to the corresponding chromosomal length. However, the genome of strain 5008 was predicted to have a relatively small core region (5.56 Mb), with a left arm of 3.16 Mb and a right arm of 1.43 Mb (Figure 1A). Syntenic analyses showed that, except for *S. scabies*, large continuous or separate inversions centered at *oriC* were detected in the chromosome of strain 5008, when compared with other *Streptomyces* species (Additional file 3: Figure S3A).

To further identify commonly conserved or species-specific proteins in strain 5008, orthologs shared among the seven *Streptomyces* strains were analyzed by MBGD [33]. The results showed that 2,954 SHJG proteins (33.3% of the total CDSs), 2,899 SCO proteins (37.3%), 2,901 SAV proteins (38.3%), 2,879 SGR proteins (40.3%), 2,989 SCAB proteins (33.4%), 2,989 SBI proteins (29.8%), and 2,806 SCLAV proteins (49.1%) could be classified into 2,754 clusters (Additional file 3: Figure S3B). The major conserved proteins are assigned with functions for transcription, translation, energy production, and amino acid and carbohydrate metabolisms (Additional file 4: Table S1). Notably, 1,640 strain-specific orthologous clusters including 1,749 proteins for strain 5008 could be detected. Surprisingly, the *amfABST* cluster [34] and the *ramR*-activated gene (*rag*) cluster for aerial-mycelium formation and sporulation [35] were not found in strain 5008 (Additional file 5: Table S2).

### Gene clusters for secondary metabolites

Totally 29 gene clusters were identified in the chromosome of strain 5008. Twenty are located in sub-telomeric regions with 14 in the left arm and 6 in the right arm. The VAL-A gene cluster (*val*) is located at a region 350 kb away from the left end of the chromosome (Figure 1A). Interestingly, the cluster for a peptidyl antibiotic jingsimycin is situated near the right end, which was found to be identical to cyclothiazomycin from *S. hygroscopicus* var. *yingchengensis* 10*–*22 [36] (Additional file 6: Table S3). Among additional 27 gene clusters putatively for secondary metabolites, 6 were assigned for the biosynthesis of polyketides (PKS), 8 for non-ribosomal peptides (NRPS), 5 for hybrid PKS-NRPSs, 4 for terpenoids, 1 for lantibiotics, and other 3 for melanin, norcardamine siderophore, and ochronotic pigment (Additional file 6: Table S3), respectively.

### Primary metabolism and precursors for validamycin production

**Figure 2:**
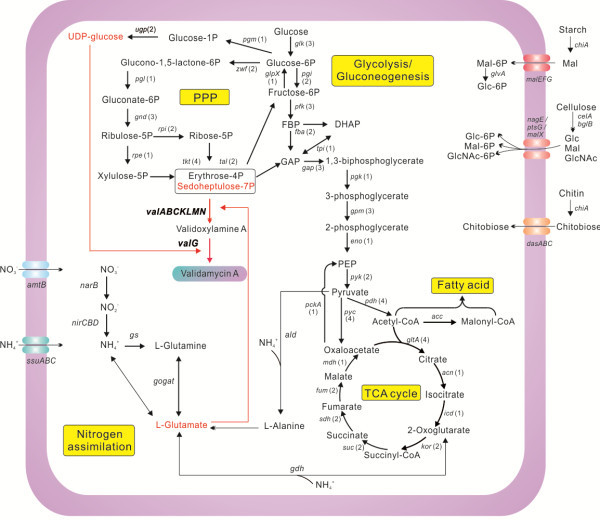
**Schematic diagram of central carbon and nitrogen metabolisms for validamycin A production in strain 5008.** Red arrows and characters represent metabolic pathways and precursors directly related to VAL-A biosynthesis, respectively. Numbers in parentheses indicated copy numbers of annotated proteins or complexes in the metabolic pathways. Abbreviation: Glc, glucose; Mal, maltose; GlcNAc, *N*-acetylglucosamine; FBP, fructose-1, 6-bisphosphate; GAP, glyceraldehyde-3 phosphate; DHAP, dihydroxyacetone phosphate; PEP, phosphoenolpyruvate; ALD, alanine dehydrogenase; GS, glutamine synthetase; GOGAT, glutamate synthase; GDH, glutamate dehydrogenase.

The production of secondary metabolites highly depends on the availability of primary metabolic building blocks [37]. Similar to most *Streptomyces*, the central carbon metabolism of strain 5008 includes complete glycolysis, the pentose phosphate pathway (PPP), the tricarboxylic acid (TCA) cycle, and gluconeogenesis pathway with multiple copies of genes encoding key enzymes for these pathways (Figure 2, in Additional file 7: Table S4).

VAL-A synthesis requires sedoheptulose 7-phosphate and UDP-glucose derived from carbohydrate metabolism as precursors. The UDP-glucose synthesis is possibly catalyzed by UDP-glucose-1-phosphate uridylyltransferases (Ugp), SHJG4652 and SHJG7333 (sharing 77% identity), preceded by the isomerization of glucose-6-phosphate to glucose-1-phosphate catalyzed by phosphoglucomutase (SHJG1995). Unlike strain 5008, only one copy of *ugp* gene is present in other sequenced *Streptomyces* genome, implicating a stronger carbon fluxes from glucose to UDP-glucose for the VAL-A synthesis in strain 5008 (Figure 2). We have recently showed that when a *ugp* gene (*SHJG4652*) was overexpressed in an industrial VAL-A producer, increased VAL-A and synchronously decreased validoxylamine A titers were achieved [23].

### A vast array of proteins for hydrolysis, transportation, and regulation

Compared with other six *Streptomyces* species, strain 5008 has relatively larger proportion of CDSs encoding α/β hydrolases (46 vs. 34.4 on average) and MFS transporters (132 vs. 99.3 on average) (Additional file 8: Table S5). There are numerous secreted hydrolases comprising α/β hydrolases (46), proteases/peptidases (155), chitinases/chitosanases (9), cellulases/endoglucanases (5), and amylases/pullulanases (6) (Additional file 9: Table S6), suggesting strain 5008 could use a wide range of carbohydrates, including a variety of mono-/oligo-saccharides, chitins, cellulose and starch.

Furthermore, strain 5008 has a complex regulatory network to coordinate the expression of genes involved in metabolism and differentiation, principally including two-component systems (TCSs, 53 paired and 28 unpaired), transcriptional regulators (751), sigma (62)/anti-sigma (5)/anti-anti-sigma (11) factors, and serine/threonine/tyrosine protein kinases (36) (Additional file 9: Table S6). Nevertheless, abundant magnesium or manganese-dependent protein phosphatases (PPMs, 54), including ValP of the VAL-A biosynthetic gene cluster, are clustered in strain 5008 (Additional file 8: Table S5). Some of PPMs are usually involved in sigma B(σ^B^)-mediated responds to stress, including heat, osmosis, oxidation responds [38], which agrees well with the VAL-A fermentation process at higher temperature of 37°C. Whereas each of other *Streptomyces* strain usually has 1 γ-butyrolactone biosynthetic gene and 1–3 receptor genes [39], strain 5008 contains 3 and 6 homologs of γ-butyrolactone biosynthetic genes and receptor genes, respectively (Additional file 10: Table S7), signifying a more complicated signaling network in this species.

### Transcriptional profiling at different cultivation temperatures

**Figure 3:**
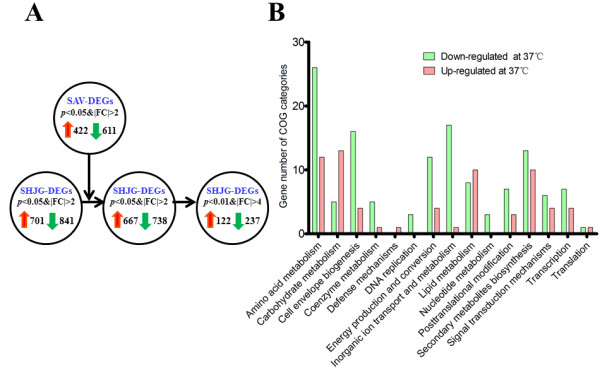
**Transcriptomic analysis of strain 5008 (SHJG) and *S. avermitilis* NRRL8165 (SAV) response to elevated temperature.** (**A**) Differentially expressed genes (DEGs) at 37°C and 30°C. The specific and common DEGs in 5008 and NRRL8165 were listed in Table S8 in Additional file 13; (**B**) COG category distributions of DEGs in 5008 by DNA microarray (*p* < 0.01, n = 4). >4-fold up-regulated (red); >4-fold down-regulated (green).

To explore the molecular mechanism of the positive thermo-regulation on VAL-A biosynthesis, the transcriptomes of strain 5008 cultured at 30°C or 37°C in liquid medium were compared by microarray analysis. Given more shared orthologs (4,845) between strain 5008 and *S. avermitilis* NRRL 8165, we chose *S. avermitilis* cultivated under the same conditions as a filter. Using the statistical criteria of >2-fold change and *p* < 0.05, a total of 1,542 differentially expressed genes (DEGs) were identified at 37°C in strain 5008 (Figure 3A). Likewise, 1,033 genes were differentially transcribed by NRRL8165 under the same cultivation condition (Figure 3A). Filtered with the DNA microarray dataset from NRRL8165, the number of DEGs in 5008 was reduced to 1,405, and subsequently to 359 using more stringent criteria of >4-fold change and *p* < 0.01 (Figure 3A).

The markedly down-regulated DEGs at 37°C are largely assigned with functions for amino acid transport and metabolism, inorganic ion transport and metabolism, and cell envelope biogenesis (Figure 3B). Consistent with previous observation [5], numerous ribosomal protein genes were moderately up-regulated at 37°C. As expected, the transcriptional levels of most of the VAL-A biosynthetic genes were markedly enhanced at 37°C, except for glucosyltransferase gene *valG*, transporter gene *valH*, and the two-component regulatory genes *valP and valQ*. Furthermore, other three gene clusters of PKS-NPRSs and type-III PKS were also up-regulated by the strain at 37°C (Table 2, Additional file 11: Table S8).

**Table 2:** **Selected differentially expressed genes in *S. hygroscopicus* 5008 at 37**°C **compared with at 30**°C

**Description**	**Gene ID or name**	**Fold change**
**Up-regulated genes at 37°C**		
Validamycin biosynthesis	*valABCD, valKLMN, valEF, valIJ*	4.1-23.8
PKS-NRPS biosynthesis	*SHJG0303-SHJG0325*	6.8-707.3
PKS-NRPS biosynthesis	*SHJG1907-SHJG1918, SHJG1921-SHJG1929*	2.1-11.8
Type III PKS biosynthesis	*SHJG8479*	12.9
Central carbon metabolisms	*SHJG3123 (gntK), SHJG3510 (citE)*	4.6-5.2
Nitrogen metabolism	*SHJG7666 (gdhA), SHJG7685 (glnA3)*	2.7-3.0
Ribosomal proteins	*SHJG0705-SHJG0706, SHJG0835-SHJG0836, SHJG2929, SHJG5163-SHJG5164, SHJG5778, SHJG5809-SHJG5816*	2.1-3.6
Heat shock proteins	*SHJG4359 (hspX), SHJG5369-SHJG5372 (dnaK-grpE-dnaJ-hspR), SHJG7073, SHJG8393*	3.3-5.3
Markedly expressed regulators	*SHJG0301, SHJG0319, SHJG0322, SHJG0477, SHJG6588, SHJG6678, SHJG6961, SHJG7352*	4.3-128.2
**Down-regulated genes at 37°C**		
Glycolysis	*SHJG2653 (pfk), SHJG3403 (gap), SHJG3488 (pyk)*	2.6-3.5
Nitrogen metabolism	*SHJG2665 (ureD), SHJG2667 (ureE),SHJG2668-SHJG2670 (ureCBA), SHJG3687 (glnA),SHJG3702 (glnII), SHJG3730 (glnA2),SHJG3958 (nirB),SHJG4429 (narK), SHJG4923 (glnR),SHJG6704 (amtB),SHJG6705 (glnB),SHJG6706 (glnD)*	4.1-16.7
Phosphate starvation response	*SHJG0329 (phoA), SHJG2705, SHJG4290 (ppe), SHJG4865 (phoU), SHJG4934 (ppk), SHJG4942-SHJG4945 (pstBACS), SHJG5998-SHJG5999 (neuAB), SHJG8008 (glpQ), SHJG8009*	3.9-144.3
Sulfate assimilation	*SHJG1828-SHJG1830, SHJG4909 (cysA), SHJG7116, SHJG7181-SHJG7183 (ssuABC), SHJG7184-SHJG7187 (cysCNDH), SHJG7188, SHJG7189 (cycI)*	4.7-16.8
Markedly expressed regulators	*SHJG0180, SHJG1204, SHJG1557, SHJG4402, SHJG4865, SHJG5108, SHJG5332, SHJG6822, SHJG7752, SHJG8626, SHJG8650,SHJG8654, SHJG8656, SHJG8657*	4.3-31.3

Differentially expressed genes both in strain 5008 and NRRL 8165 at 37°C relative to at 30°C are underlined.

Although the genes involved in pentose-phosphate pathway were not dramatically overexpressed at 37°C, key enzymes for glycolysis were moderately down-regulated, including the 6-phosphofructokinase (Pfk), glyceraldehyde-3-phosphate dehydrogenase (Gap), and pyruvate kinase (Pyk). Moreover, gluconokinase (GntK) for phosphorylation of gluconate to generate 6-phosphogluconate and citrate lyase (CitE), catalyzing the cleavage of citrate to yield oxaloacetate and acetyl-CoA, had notably enhanced transcription, which could increase the carbon flux to pentose-phosphate pathway and decrease the TCA cycle (Figure 2, Table 2, Additional file 11: Table S8).

**Figure 4:**
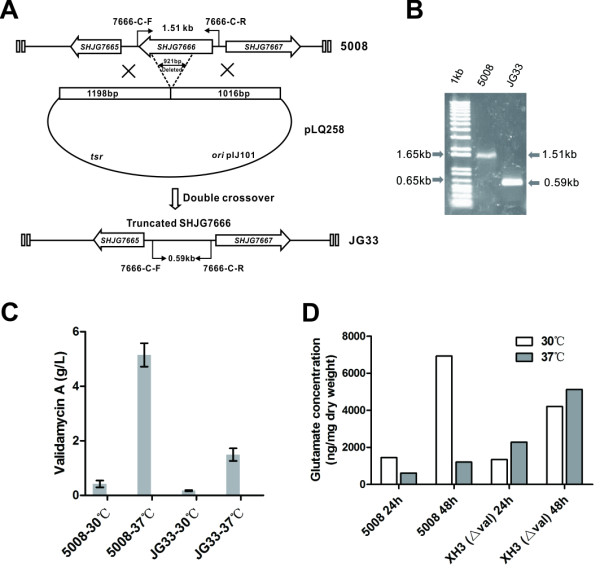
**Inactivation of the glutamate dehydrogenase gene *SHJG7666*, and measurement of intracellular glutamate (Glu) in strain 5008 and its mutant XH3 with validamycin gene cluster deleted.** (**A**) Schematic deletion of *SHJG7666* in strain 5008. (**B**) PCR analysis of strain 5008 and *SHJG7666* mutant JG33. (**C**) VAL-A production in strain 5008 and mutant JG33 cultivated at 30°C or 37°C. Mean values of three independent experiments with SD are indicated by error bars. (**D**) Concentration of intracellular Glu in strain 5008 and mutant XH3. Samples were extracted in strain 5008 and mutant XH3 for one or two days at 30°C or 37°C. Each value is the average of two measures using an amino acid analyzer. XH3 is a mutant with *val* gene cluster deleted.

A glutamine synthetase gene *glnA* and its positive regulatory gene *glnR*[40] were also down-regulated by strain 5008 at 37°C, suggesting a low concentration of glutamine and a high concentration of ammonium accumulated in bacterial cells. On the other hand, the gene of glutamate dehydrogenase GdhA (*SHJG7666*) for converting 2-oxoglutarate into L-glutamate was moderately up-regulated at 37°C, implying a mechanism for generating more amino group for VAL-A biosynthesis. Accordingly, *SHJG7666* was deleted in strain 5008, and a desired mutant JG33 was obtained (Figure 4A-B). HPLC analysis of the extracts from the mutant JG33 displayed obvious reduction of VAL-A production (Figure 4C).

Also, the intracellular concentration of glutamate in strain 5008 and its mutant XH3, with *val* gene cluster deleted (Additional file 12: Figure S4A, Additional file 13: Method SI), was quantified after 24- and 48-h cultivation using an amino acid analyzer. In 48-h cultured mutant XH3 with validamycin productivity abolished, the intracellular glutamate concentration at 37°C (5,123 ng/mg dry weight) was higher than that at 30°C (4,201 ng/mg dry weight), indicating an efficient synthesis of glutamate in *S. hygroscopicus* 5008 and its derivatives at both temperatures. Moreover, when validamycin was over-produced in strain 5008 at 37°C for 48 h, the intracellular glutamate concentration dropped to 1,203 ng/mg dry weight, less than a fourth of mutant XH3 and a fifth of strain 5008 cultivated at 30°C (6,933 ng/mg dry weight) (Figure 4D). Therefore, the dramatic decrease of intracellular glutamate concentration and the synchronic accumulation of VAL-A in strain 5008 indicated most of the glutamate was consumed for VAL-A biosynthesis at the higher temperature.

### A SARP-family regulator involved in the thermo-regulation of VAL-A biosynthesis

**Figure 5:**
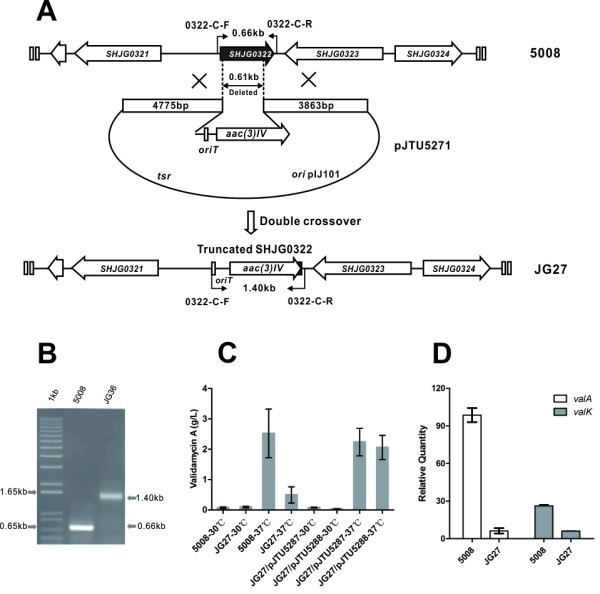
**Inactivation of the regulatory gene *SHJG0322*.** (**A**) Schematic replacement of an internal 610 bp fragment of *SHJG0322* with *aac(3)IV-oriT* cassette. (**B**) PCR analysis of strain 5008 and *SHJG0322* mutant JG27. (**C**) VAL-A production in strain 5008, mutant JG27, JG27/pJTU5287 (with PermE* promoter), and JG27/pJTU5288 (with native promoter) cultivated at 30°C or 37°C. Mean values of three independent experiments with SD are indicated by error bars. (**D**) Relative transcriptional levels of *valA* and *valK* in strain 5008 or JG27 at 37°C against 30°C.

Among the 22 markedly expressed regulators by strain 5008 at 37°C (Table 2, in Additional file 11: Table S8), a SARP-family regulatory gene (*SHJG0322*) was most highly expressed, with a maximum enhancement of 128-fold. *SHJG0322* was inactivated by replacing a 610-bp internal sequence with the apramycin resistance gene *aac(3)IV* in strain 5008, generating a thiostrepton-sensitive, apramycin-resistant (Thio^S^Apr^R^) mutant (JG27) (Figure 5A-B).

The wild-type 5008 and the *SHJG0322* mutant JG27 were cultivated at 30°C or 37°C for two days, and the extracts of these cultures were analyzed by HPLC. At 30°C, the mutant produced 0.07 g/L VAL-A, similar to the amount produced by the wild-type 5008 (0.09 g/L). At 37°C, however, the yield of VAL-A in the mutant JG27 was 0.49 g/L, which was less than 20% of the wide-type productivity (2.52 g/L) (Figure 5C). Detected by quantitative RT-PCR, the relative transcription of VAL -A biosynthetic genes *valA* and *valK* of the wild-type 5008 were increased by 100-fold and 26-fold at 37°C than at 30°C, respectively (Figure 5D). However, the transcription of *valA* and *valK* in the mutant JG27 at 37°C were both dropped to only 6-fold than at 30°C (Figure 5D).

Furthermore, the mutant JG27 was complemented with a cloned *SHJG0322* under the control of the PermE* constitutive promoter (pJTU5287) or its native promoter (pJTU5288). A similar amount of VAL-A was produced in both complemented derivatives JG27/pJTU5287 (2.23 g/L) and JG27/pJTU5288 (2.06 g/L) at 37°C, which accounted respectively for 88.6% and 81.6% of the wild-type yield (Figure 5C). At 30°C, both strains produced comparable amounts of VAL-A to strain 5008 and mutant JG27 (Figure 5C). These results suggested that the SARP gene *SHJG0322* was necessary but not adequate for the thermo-regulated validamycin biosynthesis in strain 5008. Probably some other regulatory factors are recruited as well.

## Discussion

Frequently, DNA recombinations including gene duplication, deletion, and acquisition were observed in or near the long-terminal inverted repeats of the linear chromosome ends in *Streptomyces*[41]. However, the terminal inverted repeats of the chromosome of strain 5008 are 14 bp, particularly similar to that of the chromosome of earlier industrial strain *S. avermitilis*[42], suggesting a stable phenotype under higher temperature condition. Unexpectedly, we found the variation in length for the core regions of seven *Streptomyces* chromosomes from 5.50 to 7.25 Mb, apparently different from a previous claim, which was deduced from three chromosomes with similar sizes and certain close taxonomical relatedness [26]. Therefore, apart from the chromosome arms, the variation for the core regions could also serve as an auxiliary strategy for *Streptomyces* genome plasticity, e.g. a major deletion in the central region of *S. avermitilis* was identified [43]. Also, the presence of multiple-copy protein families for central carbon metabolism secures a vigorous and regulable primary metabolism in strain 5008, which probably provides various precursors for secondary metabolism in different niches.

As revealed by the transcriptome, many genes seemed to be involved in the enhanced VAL-A yield at 37°C. Besides the significantly enhanced expression of VAL-A biosynthetic genes as previously detected [4], a few key enzymes (Pfk-Gap-Pyk-GntK-CitE) for central carbon metabolism were differentially expressed to redirect carbon metabolic flux into the pentose phosphate pathway, which generates more carbon precursors for VAL-A production (Figure 2). Moreover, the lower transcripts of most nitrogen assimilation genes and enhanced expression of the glutamate dehydrogenase gene at 37°C suggested a condition of high nitrogen supply. Also, when the validamycin gene cluster was deleted, intracellular glutamate concentration at 37°C was higher than that at 30°C (Figure 4D). When validamycin was overproduced at 37°C by strain 5008, the intracellular glutamate concentration in the two-day culture was less than a fifth of that at 30°C (Figure 4D), indicating that the glutamate was the most probable primary nitrogen source for VAL-A biosynthesis, which agrees well with previous feeding experiments with isotope-labeled precursors [11,12].

Also, the majority of genes involved in nutrient stress responses, especially the phosphate metabolism, displayed lower transcriptional profiles at 37°C in strain 5008 (Table 2). It was demonstrated that the production of secondary metabolites was generally impeded by excessive inorganic phosphate [44]. Therefore the low expression of phosphate assimilation genes probably results in a low concentration of intracellular inorganic phosphate, which is essential for VAL-A overproduction. Previous reports revealed that these genes for nutrient stress responses are usually mediated by sigma factor σ^B^ or pleiotropic antibiotic regulator AfsS [45,46]. Thus it appears that specific regulatory genes operate in a direct or indirect manner to inhibit the transcription of these genes at 37°C in strain 5008.

At 37°C, the SARP-family transcriptional regulator SHJG0322 displayed significantly enhanced expression, whose involvement in the thermo-regulation of VAL-A biosynthesis was confirmed through gene inactivation/complementation and transcriptional analysis (Figure 5). Orthologs of SHJG0322 with bacterial transcriptional activation domain (BTAD) are widely distributed in actinomycetes, including AfsR for global secondary metabolite regulation (identities 42%) [47], RedD of *S. coelicolor* for undecylprodigiosin biosynthesis (identities 37%) [48], etc. However, the 205-aa SHJG0322 is the shortest and the only identified protein related to thermo-regulation so far among its orthologs. It may mediate the interactions with RNA polymerase, other transcription complex proteins, or downstream pathway-specific regulators for the thermo-regulated VAL-A biosynthesis [49]. Furthermore, three up-regulated genes encoding one ECF sigma factor (*SHJG4152*) and two heat shock proteins (*SHJG4359SHJG8393*) at 37°C were individually inactivated by homologous recombination in strain 5008 (Additional file 12: Figure S4B-D). Obvious reduction of VAL-A yield was detected in each mutant (Additional file 12: Figure S4E), implying that the SARP gene *SHJG0322* might be under the control of sigma factor or heat stress genes. Nevertheless, regulatory mechanism of the SARP gene remains to be further studied. The identification of SHJG0322 would be served as a starting point to investigate the thermo-regulatory cascade for VAL-A biosynthesis.

## Conclusions

In conclusion, the genome of the VAL-A producer *S. hygroscopicus* 5008 was completely sequenced, and the thermo-regulated VAL-A biosynthesis was investigated through transcriptomic analysis, which highlighted unique features for VAL-A production and identified a SARP regulator positively involved in VAL-A biosynthesis. In perspective, the complete genome sequence of *S. hygroscopicus* 5008 will highly facilitate the elucidation of the metabolic and regulatory networks for VAL-A biosynthesis, rational design of high-titer VAL-A producers, and mining of its potentials for secondary metabolism within the context of functional genomics.

## Methods

### Genome sequencing and assembly

*S. hygroscopicus* var. *jinggangensis* 5008 genome was sequenced by 454 GS FLX sequencer [50], which resulted in 893,215 reads and provided 17.7-fold coverage. Plasmid library (6–8 kb inserts) and fosmid library (35–45 kb inserts) were respectively constructed with genomic DNA of strain 5008, and end-sequenced to provide contig linkage information. Gaps were filled by primer walking, subcloning, or multiplex PCR. Final sequence assembly of 900,758 reads was done using Phred/Phrap/Consed package, including 893,215 reads from 454 GS FLX, 2,756 from plasmid ends, 3,355 from fosmid ends, and 1,432 specific PCR products and primer walking.

The telomere sequences of the linear chromosome and the linear plasmid pSHJG1 were separately determined as follows. The location of each telomere was identified in the genome using *ttrA* gene, which encodes a putative helicase and generally exists in the terminal regions of *Streptomyces* chromosomes or linear plasmids [30]. Each *ttrA* homologous sequence was amplified and used as probe to localize the corresponding telomere by Southern blotting against genomic DNA, which was digested with appropriate restriction enzymes and separated by agarose gel electrophoresis. Subsequently, the fragments with positive signals were purified from the agarose gel, treated with 0.1- 0.2 M NaOH to remove the terminal proteins, and ligated with pBlueScript II SK digested with a blunt-end enzyme and the same enzyme used for the genomic DNA digestion. The correct recombinant plasmids were verified by restriction digestion and sequencing.

Data of Solexa and Sanger re-sequencing were used to revise the homopolymer error in 454 raw data and the low-quality (phrap score <40) bases in assembled sequence. Finally, the genome sequence was estimated to have an error rate of <1/10,000 bases (Phrap score ≥40).

### Genome annotation and analysis

Putative protein-coding sequences (CDSs) were predicted using Glimmer 3.02 [51] trained with all annotated CDSs of published complete *Streptomyces* genomes and Z-Curve [52] software. CDS annotation was based on the BLASTP program with NR, COG, KEGG and CDD databases, followed by manual inspection. The tRNA and transfer-messenger RNA genes were predicted using the tRNAscan-SE [53] and ARAGORN [54] programs, respectively. Pair-wise alignments between the 5008 genome and published *Streptomyces* genomes were performed using Nucmer or Promer program of the MUMmer package [55]. Proteins were clustered using the BLASTCLUST program under the conditions of a minimum of 30% identity and 70% length coverage. Ortholog analysis was submitted to the MBGD platform with default parameters [33]. Clustered regularly interspaced short palindromic repeats (CRISPRs) were identified using CRISPR Finder [56].

### DNA microarray analysis

An array of 15,000 specific 60-mer oligonucleotides was designed based on predicted CDSs from *S. hygroscopicus* 5008 and *S. avermitilis* NRRL8165, respectively. The oligonucleotides were synthesized and printed onto a glass slide according to the manufacturer’s protocol (Agilent).

Strain 5008 or NRRL8165 were pre-cultured at 30°C for 48 h in 50 ml TSB liquid medium plus 1% yeast extract in 250 ml shaking flasks with reciprocal shaking (220 rpm). 0.5 ml of each culture was inoculated into 50 ml liquid fermentation medium (gram per liter: rice 4.95%, peanut cake 0.9%, KH_2_PO_4_ 0.035%, NaCl 0.07%, and CaCO_3_ 0.03%, pH 7.5) in three 250 ml shaking flasks. The batch cultures were incubated at 30°C or 37°C for 48 h (220 rpm). Total RNA was isolated using the Trizol reagent according to the manufacturer’s instructions (Invitrogen). The RNA was purified by QIAGEN RNeasy Mini Kit, and the quality and quantity was assessed using the Agilent Bioanalyzer 2100 system. 2 μg RNA was used to synthesize cDNA, which was further transcribed into cRNA using a transcription mix containing aa-UTP and T7 RNA polymerase*.* The Cy3-labeled cRNA was purified by QIAGEN RNeasy Mini Kit. Hybridization was performed in an Agilent Microarray Hybridization Chamber (Agilent G2545A) for 17 h at 65°C. After hybridization, the slides were washed in Gene Expression Wash Buffer (Agilent), and the microarrays were scanned (Agilent G2565BA).

Using Agilent Feature Extraction Software, acquisition and quantification of array images were performed to normalize raw data with Quantile algorithm. Normalized expression ratios were calculated for each gene and tested for significance with the criteria |fold change| > 2.0 and *p* < 0.05. The change value with the lowest *p* value in a statistical analysis (*t* test) was employed as the most reliable one.

To represent the variation in triplicate measurements for each culture condition with one technical replicate, the coefficient of variation (CV) was estimated. Among the four conditions including sixteen samples, at least 98% of the genome yielded detectable transcripts, and the average coefficient of variation did not exceed 0.15, as recommended by Agilent for the quality control (Additional file 14: Table S9). Furthermore, these data were verified by qRT-PCR analysis of 8 randomly selected genes in strain 5008. Good consistency was shown between DNA microarrays and qRT-PCR analysis in terms of transcriptional changes (Additional file 15: Table S10), indicating the reliability of the microarray data.

### Quantitative RT-PCR analysis

The transcriptional levels of *valA*, *valK*, and 8 selected genes were determined by quantitative RT-PCR. Specific primers were designed using Primer 5.0 as shown in Additional file 16: Table S11. Total RNA was isolated from the mycelia cultured in fermentation medium collected at 48 h in 30°C or 37°C. The concentration of total RNA treated with DNase I (MBI Fermentas) was measured with Nanodrop (Thermo). Reverse transcription was achieved using cDNA Synthesis Kit (MBI Fermentas). Real-time PCR reactions were performed on the Applied Biosystems 7500 system with Maxima^TM^ SYBR Green/ROX qPCR Master Mix (MBI Fermentas). The *hrdB* gene encoding the major sigma factor in *Streptomyces* was used as the internal control.

### Measurement of intracellular free glutamate concentration

The amino acid analyzer (HITACHI L-8900) was used for the quantification of intracellular free glutamate of two-day cultured strain 5008 or mutant XH3 with *val* gene cluster deleted at 30°C or 37°C (220 rpm) with the ninhydrine colorimetric method. The mycelia were collected from the fermentation medium by centrifugation at 12,000 rpm for 5 min, resuspended in 1 mL ddH_2_O, and disrupted by sonication in an ice-bath (20 cycles of 5 s sonication with 10 s intervals). Cell debris was removed by centrifugation at 12,000 rpm for 5 min. The supernatants were mixed with 10% salicylsulfonic acid for 20 min at −20°C, and separated by centrifugation at 12,000 rpm for 60 min. Then, the extracts were evaporated, resuspended in 0.02 M HCl, and filtered with a 0.22 μm water-phase filter. 20 μL of each sample was injected into the amino acid analyzer for glutamate measurement.

### Inactivation of the glutamate dehydrogenase gene *SHJG7666*

Two segments of 1.20-kb HindIII/EcoRI and 1.01-kb EcoRI/BamHI from strain 5008 were amplified using two primer pairs 7666-L-F/7666-L-R (5'-AATAAGCTTCGACATCAAGATGCGGATC-3'; 5'-AATGAATTCGGACGTGGTGGTCAACTC-3') and 7666-R-F/7666-R-R (5'-AATGAATTCCCAGACGAGTGACAGCAA-3'; 5'-AATGGATCCGCACCGATGTCCTTGAAG-3'), and ligated into HindIII/BamHI-digested pJTU1278 [57], an *E. coliStreptomyces* shuttle vector, to obtain pLQ258. Then, pLQ258 was introduced into strain 5008 by intergeneric conjugation, and single-crossover exconjugants were screened by thiostrepton resistance (Tsr^R^). Subsequently, marker-free deletion mutants JG33 were selected from the initial Tsr^R^ exconjugants after several rounds of nonselective growth, and confirmed by PCR amplification using the primer pairs 7666-C-F (5'-TCGTGGAGAAGTGGTACA-3') and 7666-C-R (5'-GCCGCTACTGACTAACTG-3').

### Inactivation and complementation of the SARP-family regulatory gene *SHJG0322*

Using Redirect Technology [58], *SHJG0322* in wild-type strain 5008 was replaced by a cassette containing an apramycin-resistant marker *aac(3)IV* and the origin of transfer *oriT* as described below. The oligonucleotides 0322-T-F (5'-CGGGACAAACTCCCCGGTGCTCCGGACAACGCGCTCCAGGCCATC -3') and 0322-T-R (5'-GGCGCCGTCATGTCGCGCACGGCCGGTTCACCGGGCGAGCGCGGTTGTAGGCTGGAGCTGCTTC-3') were used as the forward and reverse primers with the plasmid pIJ773 homologous sequences underlined. The *aac(3)IV-oriT* cassette was amplified with pIJ773 as a template by PCR and electroporated into *E. coli* BW25113/pKD46/Fosmid13B1, generating a recombinant fosmid pJTU5270. Then, a 9.2-kb KpnI fragment containing the mutated *SHJG0322* from pJTU5270 was cloned into pJTU1278 [57] to give pJTU5271. The plasmid pJTU5271 was introduced into the strain 5008 by intergeneric conjugation as described [13]. Exconjugants with double cross-overs were screened for their thiostrepton sensitivity and apramycin resistance, and total DNA was isolated and further confirmed using PCR with primers 0322-C-F (5'-TCCGGACAACGCGCTCCAGGCCATC-3') and 0322-C-R (5'-GGCCGGTTCACCGGGCGAGCGCGGT-3').

Also, the mutant JG27 was complemented with cloned *SHJG0322.* Firstly, a 618-bp *SHJG0322* was amplified using the primers 0322-A-F (5'-AATCATATGGTGGCACGGGTCCGCAAGGTACT-3') and 0322-A-R (5'-AATGAATTCTCACCGGGCGAGCGCGGTCACGG-3'), cleaved with NdeI and EcoRI, and cloned into pJTU968 to obtain pJTU5286. Then, the fragment containing PermE* and *SHJG0322* was cleaved by MunI and EcoRI digestion of pJTU5286, and cloned into the integrative vector pPM927 to generate pJTU5287. Alternatively, a 1.3-kb fragment including the 618-bp *SHJG0322* and its 706-bp upstream sequence, probably containing the native promoter, was amplified by PCR using the primers 0322-B-F (5'-AATGAATTCGGCGGTCGGCGGACACATGCGGG-3') and 0322-B-R (5'-AATGAATTCTCACCGGGCGAGCGCGGTCACGG-3'), with the engineered EcoRI sites underlined. The 1.3-kb EcoRI-digested fragment was cloned into EcoRI-digested pPM927 to generate pJTU5288. Subsequently, pJTU5287 and pJTU5288 were introduced from *E. coli* into JG27 through conjugation, and exconjugants were selected with thiostrepton as previously described [23].

Constructions of other knock-out mutants of strain 5008 in this study are described in Additional file 13: Method SI.

### Analysis of validamycin A production

VAL-A production by the wide-type strain 5008 and its derivatives were detected using high performance liquid chromatography (HPLC) analysis. The strains were cultured with 50 mL fermentation medium in 250 mL baffled flasks at 30°C or 37°C for two days (220 rpm). Fermentation broths were centrifuged at 12,000 rpm for 1 min followed by purification of the supernatant with water-phase filters. The extracted supernatants were directly loaded onto a ZORBAX-SB-C18 column for HPLC analysis [13].

### Accession numbers

The genome sequences have been deposited at NCBI under the accession numbers [GenBank: CP003275], [GenBank: CP003276] and [GenBank: CP003277]. Details of the microarray design, transcriptome experimental design, and transcriptome data have been deposited in the NCBI Gene Expression Omnibus under accession numbers [GEO: GSE35610] and [GEO: GSE35611].

## Abbreviations

ABC, ATP-binding cassette; CDSs, Protein coding sequences; DEGs, Differentially expressed genes; MFS, Major facilitator superfamily; NRPS, Non-ribosomal peptide synthetase; PKS, Polyketide synthase; PPMs, Magnesium or manganese-dependent protein phosphatases; PPP, Pentose phosphate pathway; PTS, Phosphotransferase systems; qRT-PCR, Quantitative real-time polymerase chain reaction; SARP, Streptomyces antibiotic regulatory protein; TCA, Tricarboxylic acid; TCS, Two-component systems.

## Competing interests

The authors declare that they have no competing interests.

## Authors’ contributions

LQB and HW conceived and designed the experiments. HW and HJZ were responsible for sequencing, finishing and annotation. HW, SQ and LQB performed and contributed to the microarray data processing and analysis. SQ, CYL, HW and LQB carried out the experiments and data analysis. HW, LQB and ZXD were involved in drafting the manuscript. All authors read and approved the final manuscript.

## Supplementary Material

Additional file 1 **Figure S1.** Confirmation of the two plasmids from *S. hygroscopicus* 5008. Click here for file

Additional file 2 **Figure S2.** Telomeres of *S. hygroscopicus* 5008. Click here for file

Additional file 3 **Figure S3.** Comparative analyses of the chromosomes of *S. hygroscopicus *5008 with that of other six *Streptomyces* species. Click here for file

Additional file 4 **Table S1.** COG categories of conserved and specific proteins in the seven *Streptomyces* chromosomes. Click here for file

Additional file 5 **Table S2.** Selected genes for aerial mycelium and spore formation in *S. hygroscopicus* 5008 and other sequenced *Streptomyces* genomes. Click here for file

Additional file 6 **Table S3.** Putative gene or gene clusters for secondary metabolites in *S. hygroscopicus* 5008. Click here for file

Additional file 7 **Table S4.** Central carbon metabolic pathways and corresponding gene copy numbers in *S*. *hygroscopicus* 5008. Click here for file

Additional file 8 **Table S5.** Protein families in *S*. *hygroscopicus* 5008 and other six *Streptomyces* chromosomes completed. Click here for file

Additional file 9 **Table S6.** Selected genes encoding hydrolases, and regulators as mentioned in the main text. Click here for file

Additional file 10 **Table S7.** Alignment between ArpA/AfsA from *S. griseus and S. hygroscopicus* 5008 genome with BLASTP program (e value = 1e-10). Click here for file

Additional file 11 **Table S8.** Detailed transcriptomic analysis in *S. hygroscopicus* 5008 and *S. avermitilis* NRRL8165. Click here for file

Additional file 12 **Figure S4.** Constructions of other knock-out mutants of *S. hygroscopicus* 5008. Click here for file

Additional file 13 **Method SI.** Constructions of other knock-out mutants of *S. hygroscopicus* 5008 described in this study. Click here for file

Additional file 14 **Table S9.** Quality-control parameters of DNA microarray data set in *S. hygroscopicus* 5008 and *S. avermitilis* NRRL8165. Click here for file

Additional file 15 **Table S10.** Quantitative RT-PCR analysis of selected genes in *S. hygroscopicus* 5008. Click here for file

Additional file 16 **Table S11.** Sequences of primer pairs for qRT-PCR assay. Click here for file
