# Supplementary material for: Genomic and transcriptomic insights into the thermo-regulated biosynthesis of validamycin in Streptomyces hygroscopicus 5008
Source: BMC Genomics. 2012 Jul 24;13:337. doi: 10.1186/1471-2164-13-337 (PMC3424136; doi:10.1186/1471-2164-13-337)
Supplement: Additional file 1 — Figure S1. Confirmation of the two plasmids from S. hygroscopicus 5008. [file 1471-2164-13-337-S1.docx]

**Additional file 1: Figure S1 Confirmation of the two plasmids from *S*. *hygroscopicus* 5008.** (A) Detection of the linear plasmid pSHJG1 by pulsed-field gel electrophoresis. (B) Detection the circular plasmid pSHJG2 by conventional agarose gel electrophoresis.

**
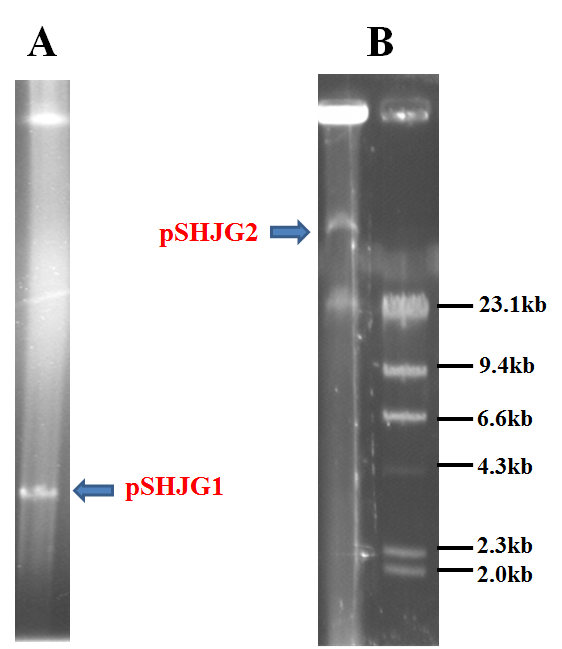
**
