# Supplementary material for: Genomic and transcriptomic insights into the thermo-regulated biosynthesis of validamycin in Streptomyces hygroscopicus 5008
Source: BMC Genomics. 2012 Jul 24;13:337. doi: 10.1186/1471-2164-13-337 (PMC3424136; doi:10.1186/1471-2164-13-337)

**Additional file 2: Figure S2 Telomeres of *S*. *hygroscopicus* 5008.** (A) Alignment of the telomere sequences from the genome of strain 5008. Palindromes are indicated by converging arrows. (B) Secondary structures of the 3'-terminal 164 nucleotides of the 5008 genome predicted by the Mfold program.


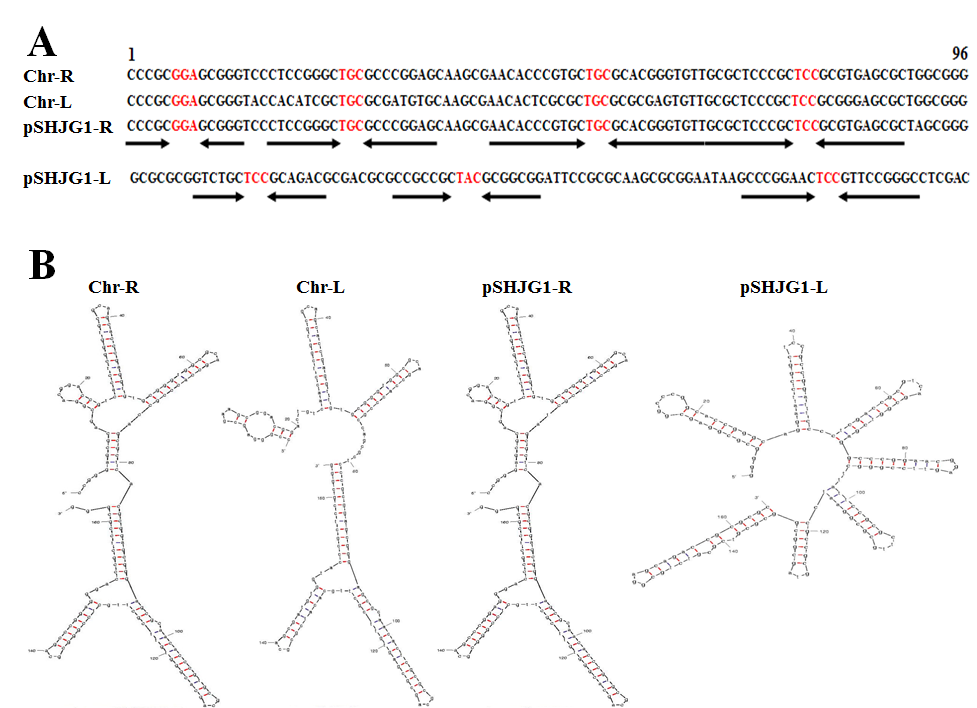

Supplement: Additional file 2 — Figure S2. Telomeres of S. hygroscopicus 5008. [file 1471-2164-13-337-S2.docx]
