# Supplementary material for: Genomic and transcriptomic insights into the thermo-regulated biosynthesis of validamycin in Streptomyces hygroscopicus 5008
Source: BMC Genomics. 2012 Jul 24;13:337. doi: 10.1186/1471-2164-13-337 (PMC3424136; doi:10.1186/1471-2164-13-337)

**Additional file 3: Figure S3** **Comparative analyses of the chromosomes of *S. hygroscopicus* 5008 with that of other six *Streptomyces* species**. **(A)** Dot-plot comparisons of the chromosomes between strain 5008 and other six *Streptomyces* species using MUMmer. Matches on the same strand are in red, and those on the opposite strand are in blue. Green bar represents the conserved core region on the 5008 chromosome. **(B)** Summary of the orthologous relationships showing cluster numbers of conserved or strain-specific genes in the seven *Streptomyces* chromosomes by MBGD platform. Abbreviation for these species: SHJG, *S. hygroscopicus* 5008; SCO, *S. coelicolor*; SAV, *S. avermitilis*; SGR, *S. griseus*; SCAB, *S. scabies*; SBI, *S. bingchenggensis*; SCLAV, *S. clavuligerus*.


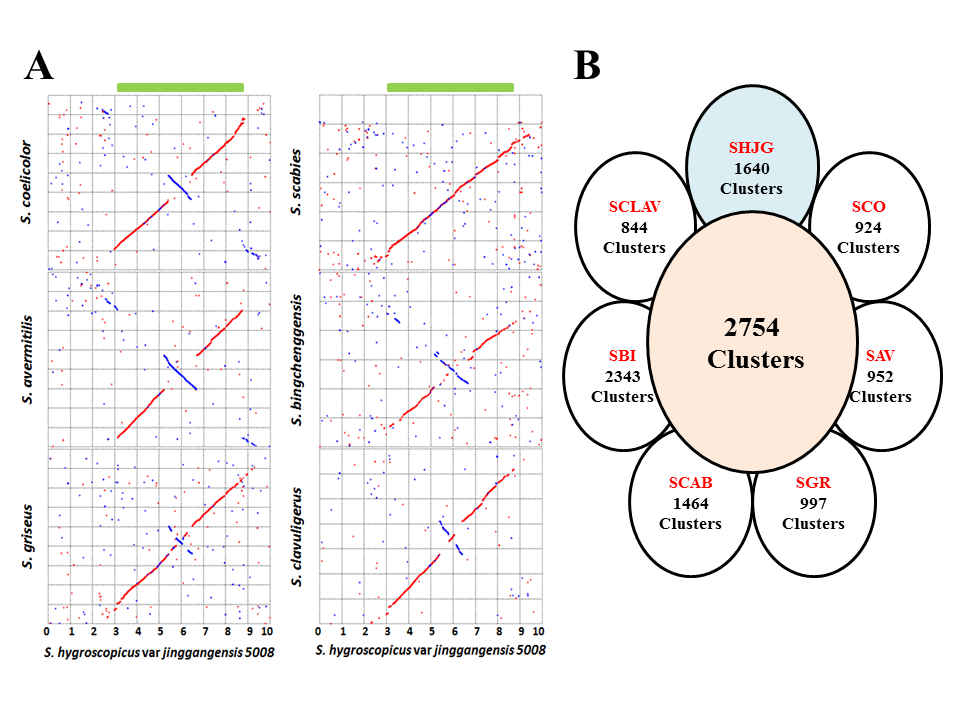

Supplement: Additional file 3 — Figure S3. Comparative analyses of the chromosomes of S. hygroscopicus 5008 with that of other six Streptomyces species. [file 1471-2164-13-337-S3.docx]
