# Supplementary material for: Genomic and transcriptomic insights into the thermo-regulated biosynthesis of validamycin in Streptomyces hygroscopicus 5008
Source: BMC Genomics. 2012 Jul 24;13:337. doi: 10.1186/1471-2164-13-337 (PMC3424136; doi:10.1186/1471-2164-13-337)
Supplement: Additional file 4 — Table S1. COG categories of conserved and specific proteins in the seven Streptomyces chromosomes. [file 1471-2164-13-337-S4.docx]

**Additional file 6: Table S1 COG categories of conserved and specific proteins in the seven *Streptomyces* chromosomes**

|  | **Total ^a^** | **Core ^a^** | **Specific ^a^** | **Core/total ^b^** | **Specific/total ^b^** |
| --- | --- | --- | --- | --- | --- |
| Total | 8868 (100) | 2954 (100) | 1749 (100) | 33.3 | 19.7 |
| Amino acid transport/metabolism [E] | 535 (6.03) | 230 (7.79) | 67 (3.83) | 43 | 12.5 |
| Carbohydrate transport/metabolism [G] | 555 (6.26) | 176 (5.96) | 59 (3.37) | 31.7 | 10.6 |
| Cell division and chromosome partitioning [D] | 39 (0.44) | 30 (1.02) | 3 (0.17) | 76.9 | 7.69 |
| Cell envelope biogenesis, outer membrane [M] | 266 (3) | 107 (3.62) | 23 (1.32) | 40.2 | 8.65 |
| Coenzyme metabolism [H] | 237 (2.67) | 114 (3.86) | 25 (1.43) | 48.1 | 10.5 |
| Defense mechanisms [V] | 135 (1.52) | 44 (1.49) | 12 (0.69) | 32.6 | 8.89 |
| DNA replication, recombination and repair [L] | 247 (2.79) | 109 (3.69) | 31 (1.77) | 44.1 | 12.6 |
| Energy production and conversion [C] | 414 (4.67) | 171 (5.79) | 62 (3.54) | 41.3 | 15 |
| Inorganic ion transport/metabolism [P] | 196 (2.21) | 79 (2.67) | 17 (0.98) | 40.3 | 8.67 |
| Intracellular trafficking and secretion [U] | 38 (0.43) | 30 (1.02) | 1 (0.06) | 78.9 | 2.63 |
| Lipid metabolism [I] | 261 (2.94) | 111 (3.76) | 29 (1.66) | 42.5 | 11.1 |
| Nucleotide transport/metabolism [F] | 128 (1.44) | 77 (2.61) | 13 (0.74) | 60.2 | 10.2 |
| Posttranslational modification [O] | 190 (2.14) | 85 (2.88) | 19 (1.09) | 44.7 | 10 |
| Secondary metabolites biosynthesis [Q] | 273 (3.08) | 68 (2.3) | 50 (2.86) | 24.9 | 18.3 |
| Signal transduction mechanisms [T] | 394 (4.44) | 140 (4.74) | 41 (2.34) | 35.5 | 10.4 |
| Transcription [K] | 662 (7.47) | 232 (7.85) | 78 (4.46) | 35 | 11.8 |
| Translation [J] | 210 (2.37) | 147 (4.98) | 11 (0.63) | 70 | 5.24 |
| Cell motility and secretion [N] | 4 (0.05) | 2 (0.07) | 0 (0) | 50 | 0 |
| RNA processing and modification [A] | 3 (0.03) | 2 (0.07) | 1 (0.06) | 66.7 | 33.3 |
| Chromatin structure and dynamics [B] | 1 (0.01) | 1 (0.03) | 0 (0) | 100 | 0 |
| General function prediction only [R] | 804 (9.07) | 285 (9.65) | 69 (3.95) | 35.4 | 8.58 |
| Function unknown [S] | 446 (5.03) | 166 (5.62) | 47 (2.69) | 37.2 | 10.5 |
| Not in COGs [-] | 2830 (31.9) | 548 (18.6) | 1091 (62.4) | 19.4 | 38.6 |

^a^ Numbers and Numbers in parentheses indicate absolute number and percentage of total, core and specific proteins within COG category in 5008 chromosome, respectively.

^b^ Numbers indicate the percentage of core and specific proteins relative to total proteins with the same COG category in 5008 chromosome.
