# Supplementary material for: Genomic and transcriptomic insights into the thermo-regulated biosynthesis of validamycin in Streptomyces hygroscopicus 5008
Source: BMC Genomics. 2012 Jul 24;13:337. doi: 10.1186/1471-2164-13-337 (PMC3424136; doi:10.1186/1471-2164-13-337)
Supplement: Additional file 5 — Table S2. Selected genes for aerial mycelium and spore formation in S. hygroscopicus 5008 and other sequenced Streptomyces genomes. [file 1471-2164-13-337-S5.docx]

**Additional file 7: Table S2 Selected genes for aerial mycelium and spore formation in *S*. *hygroscopicus* 5008 and other sequenced *Streptomyces* genomes.**

| **Name** | **SHJG ^a^** | **SCO ^a^** | **SAV ^a^** | **SGR ^a^** | **SCAB ^a^** | **SBI ^a^** | **SCLAV ^a^** |
| --- | --- | --- | --- | --- | --- | --- | --- |
| BldA | SHJGt26 | SCOt24 | SAVt57 | SGRt42 | SCABt50 | bldA ^b^ | bldA ^b^ |
| BldB | SHJG6822 | SCO5723 | SAV2529 | SGR1796 | SCAB25271 | SBI03394 | SCLAV4613 |
| BldC | SHJG5006 | SCO4091 | SAV4130 | SGR3882 | SCAB47901 | SBI05734 | SCLAV3110 |
| BldD | SHJG2914 | SCO1489 | SAV6861 | SGR6045 | SCAB75171 | SBI02366 | SCLAV0719 |
| BldG. | SHJG5461 | SCO3549 | SAV4614 | SGR3307 | SCAB40861 | SBI05854 | SCLAV2542 |
| BldH | SHJG4295 | SCO2792 | SAV5261 | SGR4742 | SCAB57831 | SBI06911 | SCLAV1957 |
| BldKA-BldKE | SHJG6208-6212; SHJG6214-6218 | SCO5112-5116 | SAV3152-3156; SAV3172-3176 | SGR2414-2418 | SCAB31501-31541 | SBI04105-04108 | SCLAV3975-3979 |
| BldM (WhiK) | SHJG5865 | SCO4768 | SAV4998 | SGR2759 | SCAB36231 | SBI06268 | SCLAV3699 |
| BldN | SHJG5601 | SCO3323 | SAV4735 | SGR4151 | SCAB39121 | SBI06006 | SCLAV2349 |
| AmfC | SHJG4904 | SCO4184 | SAV4026 | SGR3974 | SCAB49711 | SBI05067 | SCLAV3202 |
| WhiA | SHJG3405 | SCO1950 | SAV6294 | SGR5572 | SCAB69681 | SBI08027 | SCLAV1150 |
| WhiB | SHJG4500 | SCO3034 | SAV5042 | SGR4503 | SCAB55081 | SBI04463 | SCLAV2117 |
| WhiD | SHJG5864 | SCO4767 | SAV4997 | SGR2760 | SCAB36241 | SBI06267 | SCLAV3698 |
| WhiE | SHJG3678-3685 | SCO5314-5321 | SAV2837-2844 | ND | SCAB43281-43341 | SBI02761-02768 | SCLAV4912-4919 |
| WhiG | SHJG6725 | SCO5621 | SAV2630 | SGR1866 | SCAB26051 | SBI03454 | SCLAV4553 |
| WhiH | SHJG6919 | SCO5819 | SAV2445 | SGR1702 | SCAB24461 | SBI03300 | SCLAV4697 |
| WhiI | SHJG7094 | SCO6029 | SAV2230 | SGR1475 | SCAB20881 | SBI03088 | SCLAV4847 |
| CrgA (WhiP) | SHJG5212 | SCO3854 | SAV4331 | SGR3718 | SCAB45631 | SBI05399 | SCLAV2939 |
| AmfR (RamR) | SHJG3798 | SCO6685 | SAV7499 | SGR2393 | SCAB8642 | SBI7598 | SCLAV4956 |
| AmfA (RamB) | ND | SCO6684 | SAV7500 | SGR2394 | SCAB8641 | SBI7596 | SCLAV4955 |
| AmfB (RamA) | ND | SCO6683 | SAV7501 | SGR2395 | SCAB8631 | SBI7597 | SCLAV4954 |
| AmfS (RamS) | ND | SCO6682 | SAV7502 | SGR2396 | SCAB8621 | SBI7595 | ND |
| AmfT (RamC) | ND | SCO6681 | SAV7503 | SGR2397 | SCAB8611 | ND | SCLAV4953 |
| RagABKR | ND | SCO4072-4075 | SAV4142-4145 | SGR3863-3866 | SCAB47741-47771 | SBI05719-05722 | SCLAV3093-3096 |
| ChpABC | SHJG3114-7985 | SCO2716-7257-1674 | SAV6636 | SGR5829 | SCAB73031 | SBI08442 | SCLAV0900 |
| ChpDEFGH | SHJG3115-3251-4197-4205-4218 | SCO2717-1800-2705-2699-1675 | SAV6478-SAV6635 | SGR526-1375-5696-5828 | SCAB59021-59171-59251-71491-73021 | SBI08195 | SCLAV0901-3876-3900 |
| RdlAB | SHJG4219-4220 | SCO2718-SCO2719 | ND | SGR1371-1373-1376 | SCAB58991-59001-59011 | ND | SCLAV3877-3878 |

^a^ SHJG, *S. hygroscopicus* 5008; SCO, *S. coelicolor*; SAV, *S. avermitilis*; SGR, *S. griseus*; SCAB, *S. scabies*; SBI, *S. bingchenggensis*; SCLAV, *S. clavuligerus*.

^b^ These ORFs (corresponding to position 5693045-5692962 and 2610887-2610804 from the chromosome of SBI and SCLAV respectively) has not yet been detected in the submitted annotations.

ND, not determined.
