# Supplementary material for: Genomic and transcriptomic insights into the thermo-regulated biosynthesis of validamycin in Streptomyces hygroscopicus 5008
Source: BMC Genomics. 2012 Jul 24;13:337. doi: 10.1186/1471-2164-13-337 (PMC3424136; doi:10.1186/1471-2164-13-337)
Supplement: Additional file 6 — Table S3. Putative gene or gene clusters for secondary metabolites in S. hygroscopicus 5008. [file 1471-2164-13-337-S6.docx]

| **Gene clusters** | | **Length (bp)** | **Product or type** |
| --- | --- | --- | --- |
| Known (2) | SHJG0268-SHJG0294 | 43, 043 | validamycin (aminocyclitol) |
|  | SHJG8823-SHJG8839 | 23, 256 | Jingsimycin (lantibiotic) |
| PKS (6) | SHJG0717 | 1, 047 | Type III PKS |
|  | SHJG1187-SHJG1205 | 20, 575 | Type II PKS |
|  | SHJG1543-SHJG1544 | 7, 808 | Type I PKS |
|  | SHJG2643-SHJG2649 | 6, 845 | Type III PKS for HPQ melanin or flaviolin |
|  | SHJG3678-SHJG3685 | 7, 032 | Type II PKS for spore pigment |
|  | SHJG8479 | 1, 137 | Type III PKS for germidicin |
| NRPS (8) | SHJG0597-SHJG0609 | 22, 241 | NRPS |
|  | SHJG1384-SHJG1401 | 39, 660 | NRPS |
|  | SHJG1662-SHJG1673 | 36, 703 | NRPS |
|  | SHJG5926- SHJG5938 | 30, 763 | NRPS |
|  | SHJG7056- SHJG7067 | 16, 110 | NRPS |
|  | SHJG8125- SHJG8131 | 45, 965 | NRPS |
|  | SHJG8373- SHJG8378 | 6, 081 | NRPS |
|  | SHJG8672- SHJG8675 | 6, 709 | NRPS |
| PKS-NRPS (5) | SHJG0303-SHJG0327 | 25, 113 | Type I PKS, NRPS |
|  | SHJG1129-SHJG1138 | 21, 996 | PKS-NRPS hybrid, NRPS |
|  | SHJG1261-SHJG1268 | 19, 213 | Type I PKS, NRPS |
|  | SHJG1844-SHJG1855 | 20, 438 | Type I PKS, NRPS |
|  | SHJG1906-SHJG1932 | 75, 674 | Type I PKS, NRPS |
| Terpene (4) | SHJG1675-SHJG1682 | 10, 560 | Carotenoid |
|  | SHJG6335-SHJG6336 | 2, 408 | Terpene |
|  | SHJG7146-SHJG7148 | 4, 369 | Germacradienol / geosmin |
|  | SHJG7650-SHJG7654 | 6, 824 | Hopanoid |
| Lantibiotic (1) | SHJG7923-SHJG7929 | 6, 879 | Lantibiotic |
| Others (3) | SHJG4198-SHJG4199 | 1, 345 | Melanin |
|  | SHJG4283-SHJG4287 | 5, 971 | Nocardamine (sideropore) |
|  | SHJG4405-SHJG4406 | 1, 751 | Ochronotic pigment |

**Additional file 8: Table S3 Putative gene or gene clusters for secondary metabolites in *S. hygroscopicus* 5008**
