# Supplementary material for: Genomic and transcriptomic insights into the thermo-regulated biosynthesis of validamycin in Streptomyces hygroscopicus 5008
Source: BMC Genomics. 2012 Jul 24;13:337. doi: 10.1186/1471-2164-13-337 (PMC3424136; doi:10.1186/1471-2164-13-337)
Supplement: Additional file 7 — Table S4. Central carbon metabolic pathways and corresponding gene copy numbers in S. hygroscopicus 5008. [file 1471-2164-13-337-S7.docx]

**Additional file 9: Table S4 Central carbon metabolic pathways and corresponding gene copy numbers in *S*. *hygroscopicus* 5008**

| **Pathway** | **Name** | **Enzyme** | **Copy ^a^ number** | **Gene** |
| --- | --- | --- | --- | --- |
| UDP-glucose synthesis | Pgm | Phosphoglucomutase | 1 | SHJG1995 |
|  | Ugp | UTP-glucose-1-phosphate uridylyltransferase | 2 | SHJG4652-SHJG7333 |
| Pentose phosphate pathway | Zwf | Glucose-6-phosphate 1-dehydrogenase | 2 | SHJG3385-7575 |
|  | Pgl | 6-phosphogluconolactonase | 1 | SHJG3387 |
|  | Gnd | 6-phosphogluconate dehydrogenase | 3 | SHJG5193-7572-7834 |
|  | Rpi | Ribose-5-phosphate isomerase | 2 | SHJG2244-4121 |
|  | Rpe | Ribulose-phosphate 3-epimerase | 1 | SHJG2896 |
|  | Tkt | Transketolase | 4 | SHJG3383-7455-7577-7682 |
|  | Tal | Transaldolase | 2 | SHJG3384-7576 |
| Glycolysis | Glk | Glucokinase | 3 | SHJG2527-3609-7311 |
|  | Pfk | Phosphofructokinase | 3 | SHJG2653-3602-6502 |
|  | Fba | Fructose-bisphosphate aldolase | 2 | SHJG2507-5386 |
|  | Gap | Glyceraldehyde 3-phosphate dehydrogenase | 3 | SHJG1825-2035-3403 |
|  | Pgk | Phosphoglycerate kinase | 1 | SHJG3402 |
|  | Eno | Enolase | 1 | SHJG4565 |
|  | Pyk | Pyruvate kinase | 2 | SHJG3488-6499 |
| Gluconeogenesis specific | GlpX | Fructose-1,6-bisphosphatase | 1 | SHJG6144 |
|  | PckA | Phosphoenolpyruvate carboxykinase | 1 | SHJG6080 |
| Tricarboxylic acid cycle | GltA | Citrate synthase | 4 | SHJG4239-4755-6937-6938 |
|  | Acn | Aconitate hydratase | 1 | SHJG7070 |
|  | Icd | Isocitrate dehydrogenase | 1 | SHJG7759 |
|  | Kor | 2-oxoglutarate dehydrogenase complex | 2 **^b^** | SHJG0780-3659-3660-5736-5737-6033-6389-7914 |
|  | Suc | Succinyl-CoA synthetase | 2 **^b^** | SHJG5910-5911-7527-7528 |
|  | Sdh | Succinate dehydrogenase | 2 **^b^** | SHJG5977-5978-5979-5980-6202-6203-7880-7881-7882 |
|  | Fum | Fumarate hydratase | 2 | SHJG6140-6141 |
|  | Mdh | Malate dehydrogenase | 1 | SHJG5950 |

The table was generated by using the KEGG database.

**^a^** Number of predicted gene copies. **^b^** Number of annotated complexes
