# Supplementary material for: Genomic and transcriptomic insights into the thermo-regulated biosynthesis of validamycin in Streptomyces hygroscopicus 5008
Source: BMC Genomics. 2012 Jul 24;13:337. doi: 10.1186/1471-2164-13-337 (PMC3424136; doi:10.1186/1471-2164-13-337)
Supplement: Additional file 8 — Table S5. Protein families in S. hygroscopicus 5008 and other six Streptomyces chromosomes completed. [file 1471-2164-13-337-S8.docx]

**Additional file 10: Table S5 Protein families in *S*. *hygroscopicus* 5008 and other six completely sequenced *Streptomyces*.**

| **Family** | **SHJG** | **SCO** | **SAV** | **SGR** | **SCAB** | **SBI** | **SCLAV** |
| --- | --- | --- | --- | --- | --- | --- | --- |
| RNA polymerase ECF sigma factor | 41 (0.46) | 46 (0.59) | 42 (0.55) | 31 (0.43) | 49 (0.56) | 58 (0.58) | 26 (0.46) |
| RNA polymerase sigma factor | 13 (0.15) | 14 (0.18) | 13 (0.17) | 13 (0.18) | 12 (0.14) | 11 (0.11) | 10 (0.18) |
| Two-component response regulator | 72 (0.81) | 84 (1.07) | 72 (0.95) | 74 (1.04) | 96 (1.10) | 116 (1.16) | 63 (1.10) |
| Two-component histidine kinase (1) | 27 (0.30) | 44 (0.56) | 28 (0.37) | 31 (0.43) | 41 (0.47) | 48 (0.48) | 23 (0.40) |
| Two-component histidine kinase (2) | 20 (0.23) | 23 (0.29) | 25 (0.33) | 23 (0.32) | 27 (0.31) | 34 (0.34) | 20 (0.35) |
| Serine/threonine protein kinase | 31 (0.35) | 26 (0.33) | 26 (0.34) | 24 (0.34) | 43 (0.49) | 52 (0.52) | 18 (0.32) |
| Mg or Mn-dependent protein phosphatase | 54 (0.61) | 41 (0.52) | 41 (0.54) | 35 (0.49) | 44 (0.50) | 38 (0.38) | 14 (0.25) |
| TetR-family transcriptional regulator | 131 (1.48) | 133 (1.70) | 104 (1.37) | 97 (1.36) | 99 (1.13) | 150 (1.50) | 45 (0.79) |
| MarR-family transcriptional regulator | 47 (0.53) | 41 (0.52) | 34 (0.45) | 33 (0.46) | 41 (0.47) | 49 (0.49) | 17 (0.30) |
| GntR-family transcriptional regulator | 38 (0.43) | 41 (0.52) | 35 (0.46) | 28 (0.39) | 41 (0.47) | 53 (0.53) | 22 (0.39) |
| LacI-family transcriptional regulator | 29 (0.33) | 36 (0.46) | 28 (0.37) | 23 (0.32) | 46 (0.53) | 73 (0.73) | 9 (0.16) |
| LysR-family transcriptional regulator | 37 (0.42) | 40 (0.51) | 29 (0.38) | 25 (0.35) | 24 (0.27) | 52 (0.52) | 12 (0.21) |
| AraC-family transcriptional regulator | 35 (0.39) | 26 (0.33) | 24 (0.32) | 22 (0.31) | 26 (0.30) | 38 (0.38) | 9 (0.16) |
| ROK-family transcriptional regulator | 22 (0.25) | 27 (0.35) | 22 (0.29) | 16 (0.22) | 20 (0.23) | 25 (0.25) | 13 (0.23) |
| IclR-family transcriptional regulator | 16 (0.18) | 16 (0.20) | 11 (0.15) | 14 (0.20) | 24 (0.27) | 22 (0.22) | 7 (0.12) |
| AsnC-family transcriptional regulator | 12 (0.14) | 13 (0.17) | 13 (0.17) | 10 (0.14) | 16 (0.18) | 15 (0.15) | 8 (0.14) |
| DNA-binding protein | 16 (0.18) | 24 (0.31) | 26 (0.34) | 19 (0.27) | 22 (0.25) | 54 (0.54) | 26 (0.46) |
| ABC transporter ATP-binding protein | 114 (1.29) | 133 (1.70) | 134 (1.77) | 162 (2.27) | 139 (1.59) | 171 (1.71) | 99 (1.73) |
| ABC transporter permease protein | 72 (0.81) | 83 (1.06) | 74 (0.98) | 53 (0.74) | 82 (0.94) | 133 (1.33) | 30 (0.53) |
| ABC transporter sugar-binding protein | 16 (0.18) | 16 (0.20) | 14 (0.18) | 13 (0.18) | 19 (0.22) | 23 (0.23) | 4 (0.07) |
| MFS-family transporter | 132 (1.49) | 99 (1.27) | 88 (1.16) | 97 (1.36) | 87 (0.99) | 140 (1.40) | 52 (0.91) |
| Short chain dehydrogenase | 103 (1.16) | 81 (1.04) | 73 (0.96) | 57 (0.80) | 80 (0.91) | 122 (1.22) | 42 (0.74) |
| Alpha/beta hydrolase | 46 (0.52) | 35 (0.45) | 38 (0.50) | 28 (0.39) | 37 (0.42) | 43 (0.43) | 14 (0.25) |
| Cytochrome P450 | 34 (0.38) | 18 (0.23) | 32 (0.42) | 27 (0.38) | 28 (0.32) | 48 (0.48) | 32 (0.56) |
| Zn-dependent dehydrogenase | 59 (0.67) | 37 (0.47) | 44 (0.58) | 27 (0.38) | 38 (0.43) | 61 (0.61) | 17 (0.30) |
| Aldehyde dehydrogenase | 30 (0.34) | 21 (0.27) | 23 (0.30) | 22 (0.31) | 34 (0.39) | 32 (0.32) | 18 (0.32) |
| Acyl-CoA synthetase | 46 (0.52) | 25 (0.32) | 40 (0.53) | 37 (0.52) | 44 (0.50) | 65 (0.65) | 28 (0.49) |
| Acyl-CoA dehydrogenase | 17 (0.19) | 18 (0.23) | 25 (0.33) | 27 (0.38) | 23 (0.26) | 35 (0.35) | 22 (0.39) |
| Luciferase family monooxygenase | 33 (0.37) | 14 (0.18) | 26 (0.34) | 20 (0.28) | 23 (0.26) | 36 (0.36) | 13 (0.23) |
| FAD-dependent monooxygenase | 24 (0.27) | 22 (0.28) | 13 (0.17) | 12 (0.17) | 28 (0.32) | 27 (0.27) | 13 (0.23) |
| Aldo/keto reductase | 29 (0.33) | 25 (0.32) | 21 (0.28) | 19 (0.27) | 20 (0.23) | 41 (0.41) | 8 (0.14) |
| Methyltransferase | 23 (0.26) | 21 (0.27) | 24 (0.32) | 27 (0.38) | 30 (0.34) | 24 (0.24) | 11 (0.19) |
| Enoyl-CoA hydratase | 13 (0.15) | 8 (0.10) | 16 (0.21) | 14 (0.20) | 14 (0.16) | 17 (0.17) | 11 (0.19) |
| Aminotransferase | 17 (0.19) | 16 (0.20) | 14 (0.18) | 14 (0.20) | 17 (0.19) | 14 (0.14) | 16 (0.28) |

Numbers indicate absolute number of proteins from each genome in each family. Numbers in parentheses indicate the percentage of the total proteins in that genome. SHJG, *S. hygroscopicus* 5008; SCO, *S. coelicolor*; SAV, *S. avermitilis*; SGR, *S. griseus*; SCAB, *S. scabies*; SBI, *S. bingchenggensis*; SCLAV, *S. clavuligerus*.
