# Supplementary material for: Genomic and transcriptomic insights into the thermo-regulated biosynthesis of validamycin in Streptomyces hygroscopicus 5008
Source: BMC Genomics. 2012 Jul 24;13:337. doi: 10.1186/1471-2164-13-337 (PMC3424136; doi:10.1186/1471-2164-13-337)
Supplement: Additional file 9 — Table S6. Selected genes encoding hydrolases, and regulators as mentioned in the main text. [file 1471-2164-13-337-S9.docx]

**Additional file 11: Table S6 Selected genes encoding hydrolases and regulators as mentioned in the main text.**

**1. Secreted hydrolases**

**⑴ Alpha/beta hydrolases**

| **ID** | **Start** | **End** | **Size** | **Function** |
| --- | --- | --- | --- | --- |
| SHJG0097 | 128701 | 129312 | 612bp | hydrolase, alpha/beta fold family protein |
| SHJG0171 | 232801 | 233559 | 759bp | chloride peroxidase |
| SHJG0226 | 298953 | 299609 | 657bp | alpha/beta hydrolase fold |
| SHJG0421 | 551817 | 551062 | 756bp | putative hydrolase |
| SHJG0425 | 555989 | 554925 | 1065bp | alpha/beta hydrolase fold |
| SHJG0433 | 565557 | 566597 | 1041bp | hydrolase |
| SHJG0449 | 584092 | 583154 | 939bp | hydrolase |
| SHJG0554 | 698984 | 699826 | 843bp | chloride peroxidase |
| SHJG0618 | 778092 | 778928 | 837bp | non-heme chloroperoxidase |
| SHJG0773 | 949878 | 950714 | 837bp | hydrolase |
| SHJG0778 | 954645 | 955511 | 867bp | hydrolase, alpha/beta hydrolase fold family |
| SHJG0795 | 968962 | 968120 | 843bp | hydrolase |
| SHJG0804 | 982143 | 982988 | 846bp | alpha/beta hydrolase fold |
| SHJG0816 | 998468 | 997632 | 837bp | hydrolase |
| SHJG0857 | 1038697 | 1039530 | 834bp | hydrolase |
| SHJG0960 | 1146674 | 1145847 | 828bp | hydrolase |
| SHJG1081 | 1298136 | 1297360 | 777bp | hydrolase |
| SHJG1161 | 1395744 | 1396682 | 939bp | hydrolase |
| SHJG1163 | 1398954 | 1399832 | 879bp | hydrolase |
| SHJG1165 | 1402267 | 1401281 | 987bp | epoxide hydrolase |
| SHJG1293 | 1556012 | 1555143 | 870bp | hydrolase |
| SHJG1596 | 1910928 | 1911758 | 831bp | non-heme chloroperoxidase (chloride peroxidase) |
| SHJG1605 | 1929229 | 1928393 | 837bp | alpha/beta hydrolase fold |
| SHJG1691 | 2056057 | 2056887 | 831bp | non-heme chloroperoxidase (chloride peroxidase) |
| SHJG1793 | 2169422 | 2168508 | 915bp | hydrolase, alpha/beta fold family |
| SHJG1881 | 2278236 | 2277358 | 879bp | putative secreted hydrolase |
| SHJG2030 | 2487719 | 2487090 | 630bp | putative hydrolase |
| SHJG2215 | 2683276 | 2682428 | 849bp | hydrolase |
| SHJG2331 | 2806571 | 2807497 | 927bp | hydrolase |
| SHJG2457 | 2957676 | 2956555 | 1122bp | branched-chain alpha-keto acid dehydrogenase |
| SHJG2609 | 3122286 | 3121639 | 648bp | putative alpha/beta hydrolase |
| SHJG2704 | 3210784 | 3211614 | 831bp | hydrolase |
| SHJG3700 | 4289965 | 4289111 | 855bp | carboxylesterase |
| SHJG4642 | 5349896 | 5348943 | 954bp | hydrolase |
| SHJG4749 | 5466839 | 5465991 | 849bp | hydrolase |
| SHJG5284 | 6053753 | 6052971 | 783bp | hydrolase |
| SHJG5445 | 6230439 | 6229516 | 924bp | hydrolase |
| SHJG5843 | 6650869 | 6652128 | 1260bp | hydrolase |
| SHJG6267 | 7130087 | 7129281 | 807bp | hydrolase |
| SHJG6274 | 7136607 | 7137554 | 948bp | hydrolase |
| SHJG6581 | 7490736 | 7489954 | 783bp | alpha/beta hydrolase fold |
| SHJG7147 | 8157201 | 8156401 | 801bp | hydrolase |
| SHJG7349 | 8386196 | 8385321 | 876bp | alpha/beta hydrolase fold |
| SHJG7386 | 8416083 | 8417378 | 1296bp | 3-oxoadipate enol-lactone hydrolase/4-carboxymuconolactone decarboxylase |
| SHJG7604 | 8657553 | 8656432 | 1122bp | 3-oxoadipate enol-lactone hydrolase |
| SHJG8277 | 9488560 | 9487757 | 804bp | putative hydrolase |

**⑵ Proteases/peptidases**

| **ID** | **Start** | **End** | **Size** | **Function** |
| --- | --- | --- | --- | --- |
| SHJG0334 | 441958 | 440387 | 1572bp | putative serine protease |
| SHJG0335 | 443379 | 441928 | 1452bp | putative serine protease |
| SHJG0406 | 539046 | 535384 | 3663bp | secreted peptidase |
| SHJG0451 | 586063 | 587082 | 1020bp | peptidase |
| SHJG0489 | 627954 | 628985 | 1032bp | putative D-alanyl-D-alanine carboxypeptidase |
| SHJG0495 | 635456 | 634062 | 1395bp | carboxypeptidase |
| SHJG0641 | 805801 | 809607 | 3807bp | peptidase S8 and S53 subtilisin kexin sedolisin |
| SHJG0689 | 865298 | 862803 | 2496bp | peptidase S8 and S53 subtilisin kexin sedolisin |
| SHJG0751 | 926522 | 927289 | 768bp | methionine aminopeptidase |
| SHJG0860 | 1044117 | 1043215 | 903bp | peptidase S15 |
| SHJG0991 | 1177071 | 1174732 | 2340bp | neutral zinc metalloprotease |
| SHJG1036 | 1225973 | 1224762 | 1212bp | putative DD-peptidase precursor |
| SHJG1041 | 1231859 | 1232824 | 966bp | putative trypsin-like protease |
| SHJG1115 | 1332118 | 1333314 | 1197bp | peptidase |
| SHJG1123 | 1344956 | 1341837 | 3120bp | peptidase |
| SHJG1140 | 1374356 | 1375582 | 1227bp | dipeptidyl aminopeptidase/acylaminoacyl-peptidase |
| SHJG1155 | 1390465 | 1389287 | 1179bp | dipeptidase |
| SHJG1216 | 1456922 | 1456035 | 888bp | putative metalloprotease |
| SHJG1314 | 1571894 | 1571334 | 561bp | dipeptidase |
| SHJG1318 | 1576337 | 1575288 | 1050bp | metalloendopeptidase |
| SHJG1404 | 1684130 | 1683429 | 702bp | putative peptidase |
| SHJG1468 | 1748436 | 1746424 | 2013bp | peptidase |
| SHJG1476 | 1757631 | 1756375 | 1257bp | dipeptidyl aminopeptidase |
| SHJG1602 | 1920317 | 1923922 | 3606bp | secreted peptidase |
| SHJG1738 | 2107932 | 2107408 | 525bp | serine protease, subtilase family |
| SHJG1779 | 2153150 | 2152428 | 723bp | peptidase C26 |
| SHJG1856 | 2247588 | 2246236 | 1353bp | probable aminopeptidase |
| SHJG1862 | 2252543 | 2256163 | 3621bp | probable secreted peptidase |
| SHJG1916 | 2343507 | 2342611 | 897bp | proline iminopeptidase |
| SHJG1973 | 2425426 | 2424848 | 579bp | hydrogenase maturation protease |
| SHJG1990 | 2442915 | 2441053 | 1863bp | metallopeptidase |
| SHJG2060 | 2517185 | 2514786 | 2400bp | neutral zinc metalloprotease |
| SHJG2120 | 2581340 | 2579931 | 1410bp | metallopeptidase |
| SHJG2136 | 2600532 | 2598844 | 1689bp | neutral zinc metalloprotease |
| SHJG2138 | 2600934 | 2602100 | 1167bp | peptidase |
| SHJG2139 | 2603487 | 2602168 | 1320bp | serine protease precursor |
| SHJG2198 | 2661389 | 2662993 | 1605bp | protease (secreted protein) |
| SHJG2224 | 2697537 | 2694898 | 2640bp | ATP-dependent Clp protease |
| SHJG2230 | 2702955 | 2702389 | 567bp | intracellular protease/amidase |
| SHJG2589 | 3098612 | 3099940 | 1329bp | metallopeptidase |
| SHJG2664 | 3179300 | 3177735 | 1566bp | secreted tripeptidylaminopeptidase |
| SHJG2672 | 3184572 | 3185207 | 636bp | ATP-dependent Clp protease |
| SHJG2917 | 3452614 | 3451517 | 1098bp | peptidase |
| SHJG3044 | 3583692 | 3582523 | 1170bp | serine protease (membrane protein) |
| SHJG3089 | 3630834 | 3629704 | 1131bp | peptidase |
| SHJG3188 | 3734924 | 3734022 | 903bp | fibrinolytic protease precursor |
| SHJG3276 | 3831552 | 3833078 | 1527bp | subtilisin-like protease |
| SHJG3278 | 3833175 | 3834692 | 1518bp | subtilisin-like protease |
| SHJG3404 | 3966362 | 3963525 | 2838bp | carboxypeptidase |
| SHJG3492 | 4065566 | 4063074 | 2493bp | aminopeptidase |
| SHJG3553 | 4130696 | 4130046 | 651bp | putative signal peptidase II |
| SHJG3650 | 4237192 | 4238271 | 1080bp | putative secreted serine protease |
| SHJG3658 | 4246853 | 4248400 | 1548bp | leucyl aminopeptidase |
| SHJG3666 | 4258104 | 4259288 | 1185bp | serine protease |
| SHJG3735 | 4331907 | 4333847 | 1941bp | serine protease, subtilase family |
| SHJG3757 | 4354467 | 4355324 | 858bp | methionine aminopeptidase |
| SHJG3773 | 4369604 | 4368381 | 1224bp | D-alanyl-D-alanine carboxypeptidase |
| SHJG3917 | 4515981 | 4512370 | 3612bp | secreted peptidase |
| SHJG3952 | 4559492 | 4561141 | 1650bp | neutral zinc metalloprotease |
| SHJG4018 | 4629794 | 4630864 | 1071bp | metalloprotease |
| SHJG4038 | 4650711 | 4647553 | 3159bp | protease |
| SHJG4110 | 4734521 | 4733235 | 1287bp | ATP-dependent protease ATP-binding subunit |
| SHJG4111 | 4735358 | 4734693 | 666bp | ATP-dependent Clp protease proteolytic subunit |
| SHJG4112 | 4736043 | 4735426 | 618bp | putative ATP-dependent Clp protease proteolytic subunit 1 |
| SHJG4128 | 4751025 | 4753604 | 2580bp | aminopeptidase |
| SHJG4132 | 4758244 | 4757138 | 1107bp | D-alanyl-D-alanine carboxypeptidase |
| SHJG4138 | 4762968 | 4766264 | 3297bp | serine protease |
| SHJG4144 | 4770562 | 4773138 | 2577bp | aminopeptidase N |
| SHJG4261 | 4907479 | 4906064 | 1416bp | secreted tripeptidyl aminopeptidase |
| SHJG4393 | 5048491 | 5048183 | 309bp | ATP-dependent Clp protease adaptor protein ClpS |
| SHJG4398 | 5054627 | 5052270 | 2358bp | secreted protease |
| SHJG4437 | 5100261 | 5099134 | 1128bp | carboxy-terminal processing protease precursor |
| SHJG4464 | 5142493 | 5143059 | 567bp | putative intracellular protease/amidase |
| SHJG4490 | 5177045 | 5177602 | 558bp | putative signal peptidase I |
| SHJG4523 | 5214288 | 5213095 | 1194bp | dipeptidase |
| SHJG4639 | 5344378 | 5347668 | 3291bp | protease |
| SHJG4702 | 5417137 | 5419611 | 2475bp | D-alanyl-D-alanine carboxypeptidase |
| SHJG4841 | 5569173 | 5570459 | 1287bp | metallopeptidase |
| SHJG4925 | 5646379 | 5647368 | 990bp | protease |
| SHJG4972 | 5710244 | 5709207 | 1038bp | peptidase |
| SHJG5098 | 5844534 | 5843098 | 1437bp | protease (secreted protein) |
| SHJG5107 | 5851171 | 5852655 | 1485bp | aminopeptidase P |
| SHJG5260 | 6025763 | 6024861 | 903bp | D-alanyl-D-alanine carboxypeptidase |
| SHJG5271 | 6035795 | 6034545 | 1251bp | putative aminopeptidase 2 |
| SHJG5378 | 6153002 | 6155599 | 2598bp | ATP-dependent protease ATP-binding subunit |
| SHJG5471 | 6266192 | 6267805 | 1614bp | protease |
| SHJG5529 | 6316890 | 6318434 | 1545bp | D-alanyl-D-alanine carboxypeptidase |
| SHJG5565 | 6351377 | 6353899 | 2523bp | Clp-family ATP-binding protease |
| SHJG5567 | 6355104 | 6355655 | 552bp | peptidase |
| SHJG5642 | 6442138 | 6442968 | 831bp | D-aminopeptidase |
| SHJG5691 | 6498464 | 6499237 | 774bp | type IV peptidase |
| SHJG5784 | 6600076 | 6599084 | 993bp | peptidase, M48 family protein |
| SHJG5819 | 6626770 | 6627606 | 837bp | methionine aminopeptidase |
| SHJG5849 | 6654990 | 6656090 | 1101bp | O-sialoglycoprotein endopeptidase |
| SHJG5900 | 6715727 | 6714195 | 1533bp | peptidase |
| SHJG5970 | 6813060 | 6811813 | 1248bp | D-alanyl-D-alanine carboxypeptidase |
| SHJG6003 | 6845508 | 6846695 | 1188bp | peptidase |
| SHJG6053 | 6898971 | 6902177 | 3207bp | subtilisin-like protease |
| SHJG6098 | 6946038 | 6944821 | 1218bp | carboxypeptidase |
| SHJG6224 | 7088261 | 7086120 | 2142bp | peptidase |
| SHJG6239 | 7102245 | 7103330 | 1086bp | dipeptidase |
| SHJG6249 | 7110729 | 7112429 | 1701bp | protease |
| SHJG6285 | 7152999 | 7151446 | 1554bp | protease |
| SHJG6286 | 7154706 | 7153162 | 1545bp | protease |
| SHJG6292 | 7165834 | 7167237 | 1404bp | peptidase |
| SHJG6395 | 7274679 | 7277099 | 2421bp | ATP-dependent protease |
| SHJG6527 | 7429979 | 7428339 | 1641bp | neutral zinc metalloprotease |
| SHJG6529 | 7432285 | 7430231 | 2055bp | neutral zinc metalloprotease |
| SHJG6570 | 7479136 | 7480527 | 1392bp | metallopeptidase |
| SHJG6716 | 7640182 | 7640883 | 702bp | signal peptidase I |
| SHJG6717 | 7640876 | 7641967 | 1092bp | signal peptidase I |
| SHJG6718 | 7641918 | 7642835 | 918bp | signal peptidase I |
| SHJG6719 | 7642916 | 7643689 | 774bp | signal peptidase I |
| SHJG6758 | 7684168 | 7683023 | 1146bp | secreted peptidase |
| SHJG6784 | 7711997 | 7713298 | 1302bp | metalloprotease |
| SHJG6806 | 7741104 | 7742354 | 1251bp | serine protease |
| SHJG6813 | 7749442 | 7750605 | 1164bp | secreted serine protease |
| SHJG6825 | 7766452 | 7767831 | 1380bp | protease |
| SHJG6893 | 7846246 | 7847769 | 1524bp | metallopeptidase |
| SHJG6921 | 7886193 | 7887047 | 855bp | serine protease |
| SHJG6941 | 7909193 | 7910596 | 1404bp | protease |
| SHJG6942 | 7910593 | 7911981 | 1389bp | protease |
| SHJG6943 | 7912392 | 7913147 | 756bp | peptidase |
| SHJG7025 | 8011436 | 8010483 | 954bp | D-alanyl-D-alanine carboxypeptidase |
| SHJG7103 | 8115366 | 8113708 | 1659bp | secreted protease |
| SHJG7151 | 8162419 | 8163546 | 1128bp | peptidase |
| SHJG7252 | 8278098 | 8277307 | 792bp | trypsin-like protease |
| SHJG7393 | 8422668 | 8424437 | 1770bp | gamma-glutamyltranspeptidase |
| SHJG7394 | 8427142 | 8424599 | 2544bp | clp protease ATP binding subunit |
| SHJG7395 | 8427700 | 8427329 | 372bp | methionine aminopeptidase |
| SHJG7440 | 8473519 | 8473388 | 132bp | secreted peptidase |
| SHJG7475 | 8506553 | 8506011 | 543bp | protease |
| SHJG7631 | 8686854 | 8685367 | 1488bp | metallopeptidase |
| SHJG7632 | 8687818 | 8688891 | 1074bp | X-prolyl-dipeptidyl aminopeptidase |
| SHJG7755 | 8820084 | 8821523 | 1440bp | protease |
| SHJG7835 | 8922911 | 8924065 | 1155bp | peptidase |
| SHJG8043 | 9191934 | 9193235 | 1302bp | putative prolyl aminopeptidase |
| SHJG8104 | 9255842 | 9256276 | 435bp | protease inhibitor protein |
| SHJG8168 | 9366400 | 9365024 | 1377bp | putative secreted protease |
| SHJG8182 | 9377892 | 9380111 | 2220bp | putative acylaminoacyl-peptidase |
| SHJG8192 | 9393161 | 9392268 | 894bp | putative M48-family peptidase |
| SHJG8283 | 9493450 | 9493902 | 453bp | aminopeptidase |
| SHJG8288 | 9498155 | 9499297 | 1143bp | alkaline D-peptidase |
| SHJG8289 | 9499294 | 9500853 | 1560bp | putative peptidase |
| SHJG8304 | 9511664 | 9512035 | 372bp | putative S41-family peptidase |
| SHJG8310 | 9517962 | 9517633 | 330bp | peptidase |
| SHJG8394 | 9616072 | 9617280 | 1209bp | secreted serine protease |
| SHJG8419 | 9640893 | 9640204 | 690bp | secreted extracellular small neutral protease |
| SHJG8461 | 9689224 | 9688316 | 909bp | penicillin-resistant DD-carboxypeptidase |
| SHJG8680 | 9922713 | 9923585 | 873bp | metalloprotease |
| SHJG8773 | 10026434 | 10026922 | 489bp | hydrogenase maturation protease |
| SHJG8797 | 10049340 | 10048201 | 1140bp | peptidase M50 |
| SHJG8810 | 10067431 | 10067643 | 213bp | neutral zinc metalloprotease |
| SHJG8811 | 10068367 | 10067909 | 459bp | serine protease, subtilase family protein |

**⑶ Chitinases/chitosanases**

| **ID** | **Start** | **End** | **Size** | **Function** |
| --- | --- | --- | --- | --- |
| SHJG0250 | 331217 | 332602 | 1386bp | chitinase |
| SHJG2174 | 2635884 | 2635174 | 711bp | chitinase |
| SHJG2863 | 3394818 | 3396050 | 1233bp | chitinase |
| SHJG8387 | 9608264 | 9607161 | 1104bp | chitinase |
| SHJG8388 | 9609225 | 9608404 | 822bp | chitinase |
| SHJG7582 | 8630939 | 8629254 | 1686bp | chitinase A |
| SHJG6770 | 7696084 | 7697895 | 1812bp | chitinase B |
| SHJG6879 | 7833496 | 7832153 | 1344bp | putative chitinase |
| SHJG1996 | 2450250 | 2449393 | 858bp | chitosanase |

**⑷ Cellulases/endoglucanases**

| **ID** | **Start** | **End** | **Size** | **Function** |
| --- | --- | --- | --- | --- |
| SHJG1118 | 1335199 | 1336164 | 966bp | cellulase |
| SHJG1119 | 1336256 | 1337530 | 1275bp | cellulase |
| SHJG8384 | 9602281 | 9600356 | 1926bp | cellulase |
| SHJG4626 | 5331293 | 5330241 | 1053bp | cellulase |
| SHJG5828 | 6633770 | 6634789 | 1020bp | putative endoglucanase |

**⑸ Amylases/pullulanases**

| **ID** | **Start** | **End** | **Size** | **Function** |
| --- | --- | --- | --- | --- |
| SHJG2097 | 2553831 | 2555822 | 1992bp | putative alpha-amylase |
| SHJG3717 | 4309802 | 4308483 | 1320bp | alpha-amylase |
| SHJG6521 | 7423059 | 7420927 | 2133bp | alpha-amylase |
| SHJG7153 | 8166323 | 8164578 | 1746bp | alpha-amylase |
| SHJG7159 | 8174248 | 8171906 | 2343bp | alpha-amylase |
| SHJG3716 | 4308391 | 4302998 | 5394bp | pullulanase |

**2. Regulators**

**⑴ Two-component regulatory systems**

| **ID** | **Start** | **End** | **Size** | **Function** |
| --- | --- | --- | --- | --- |
| SHJG0288 | 391253 | 390372 | 882bp | ValP- Phosphatase |
| SHJG0289 | 391855 | 391250 | 606bp | ValQ- Histidine kinase |
| SHJG0487 | 625831 | 626559 | 729bp | two-component system response regulator |
| SHJG0488 | 626561 | 627805 | 1245bp | two-component system sensor kinase |
| SHJG0508 | 648872 | 650200 | 1329bp | two-component system sensor kinase |
| SHJG0509 | 650197 | 650937 | 741bp | two-component transcriptional regulator |
| SHJG0540 | 685907 | 686677 | 771bp | putative two-component system response regulator |
| SHJG0541 | 686667 | 688562 | 1896bp | putative two-component system sensor kinase |
| SHJG0929 | 1114046 | 1114807 | 762bp | two-component system response regulator |
| SHJG0930 | 1114804 | 1116264 | 1461bp | two-component system sensor kinase |
| SHJG1103 | 1321131 | 1322402 | 1272bp | two-component system sensor kinase |
| SHJG1104 | 1322399 | 1323058 | 660bp | two-component system response regulator |
| SHJG1179 | 1413999 | 1413361 | 639bp | two-component system response regulator |
| SHJG1180 | 1415216 | 1413996 | 1221bp | two-component system histidine kinase |
| SHJG1362 | 1622358 | 1621708 | 651bp | two-component system response regulator |
| SHJG1363 | 1623602 | 1622346 | 1257bp | two-component system sensor kinase |
| SHJG1922 | 2349786 | 2349142 | 645bp | two-component system response regulator |
| SHJG1923 | 2351039 | 2349750 | 1290bp | two-component system sensor kinase |
| SHJG2117 | 2578402 | 2576552 | 1851bp | putative two-component system sensor kinase |
| SHJG2118 | 2579106 | 2578399 | 708bp | putative two-component system response regulator |
| SHJG2394 | 2879886 | 2879386 | 501bp | two-component system sensor kinase |
| SHJG2395 | 2880571 | 2879888 | 684bp | putative two-component system response regulator |
| SHJG2450 | 2949234 | 2948608 | 627bp | putative two-component system response regulator |
| SHJG2451 | 2950639 | 2949263 | 1377bp | putative two-component system sensor kinase |
| SHJG2516 | 3021424 | 3020759 | 666bp | two-component system response regulator |
| SHJG2517 | 3022650 | 3021421 | 1230bp | two-component system sensor kinase |
| SHJG2697 | 3204608 | 3205762 | 1155bp | two-component system sensor kinase |
| SHJG2698 | 3205777 | 3206433 | 657bp | two-component system response regulator |
| SHJG2715 | 3224369 | 3223269 | 1101bp | two-component system sensor kinase |
| SHJG2716 | 3225019 | 3224366 | 654bp | two-component system response regulator |
| SHJG2999 | 3540676 | 3539270 | 1407bp | two-component system sensor kinase |
| SHJG3000 | 3541479 | 3540739 | 741bp | two-component system response regulator |
| SHJG3252 | 3807416 | 3806775 | 642bp | two-component system response regulator |
| SHJG3253 | 3808563 | 3807409 | 1155bp | two-component system sensor kinase |
| SHJG3603 | 4187037 | 4186363 | 675bp | two-component system response regulator |
| SHJG3604 | 4188269 | 4187034 | 1236bp | two-component system sensor kinase |
| SHJG3644 | 4232859 | 4232179 | 681bp | two-component system response regulator |
| SHJG3645 | 4234100 | 4232856 | 1245bp | two-component sensor kinase |
| SHJG3795 | 4390424 | 4391740 | 1317bp | two-component sensor kinase |
| SHJG3798 | 4394673 | 4395323 | 651bp | two-component system response regulator |
| SHJG4305 | 4961878 | 4960475 | 1404bp | two-component system sensor kinase |
| SHJG4306 | 4962573 | 4961875 | 699bp | two-component system response regulator |
| SHJG4477 | 5158356 | 5156341 | 2016bp | two-component system histidine kinase |
| SHJG4478 | 5159143 | 5158454 | 690bp | putative two-component system response regulator |
| SHJG4527 | 5217946 | 5216675 | 1272bp | two-component system sensor kinase |
| SHJG4528 | 5218706 | 5217981 | 726bp | two-component system response regulator |
| SHJG4863 | 5589442 | 5588762 | 681bp | putative two-component system response regulator |
| SHJG4864 | 5590734 | 5589439 | 1296bp | two-component system sensor kinase |
| SHJG4927 | 5647746 | 5648480 | 735bp | two-component system response regulator |
| SHJG4928 | 5648477 | 5649931 | 1455bp | two-component system sensor kinase |
| SHJG5062 | 5804505 | 5802994 | 1512bp | two-component system sensor kinase |
| SHJG5063 | 5805368 | 5804595 | 774bp | two-component system response regulator |
| SHJG5317 | 6090534 | 6089896 | 639bp | putative two-component system response regulator |
| SHJG5318 | 6091739 | 6090531 | 1209bp | putative two-component system sensor kinase |
| SHJG5393 | 6171239 | 6172597 | 1359bp | two-component system sensor kinase |
| SHJG5394 | 6172594 | 6173259 | 666bp | two-component system response regulator |
| SHJG5548 | 6337063 | 6338268 | 1206bp | two-component system sensor kinase |
| SHJG5550 | 6338300 | 6338971 | 672bp | two-component system response regulator |
| SHJG5570 | 6359801 | 6358404 | 1398bp | two-component system sensor kinase |
| SHJG5571 | 6360519 | 6359815 | 705bp | two-component system response regulator |
| SHJG5637 | 6436891 | 6437607 | 717bp | two-component system response regulator |
| SHJG5638 | 6437608 | 6439044 | 1437bp | two-component system sensor kinase |
| SHJG5738 | 6549185 | 6548526 | 660bp | two-component system response regulator |
| SHJG5739 | 6550945 | 6549620 | 1326bp | two-component system sensor kinase |
| SHJG5740 | 6552478 | 6551201 | 1278bp | two-component system sensor kinase |
| SHJG5893 | 6705609 | 6706898 | 1290bp | two-component system sensor kinase |
| SHJG5894 | 6706895 | 6707638 | 744bp | two-component system response regulator |
| SHJG5942 | 6780666 | 6781988 | 1323bp | putative two-component system sensor kinase |
| SHJG5943 | 6781952 | 6782596 | 645bp | putative two-component system response regulator |
| SHJG5947 | 6785017 | 6786156 | 1140bp | two-component system sensor kinase |
| SHJG5948 | 6786201 | 6786857 | 657bp | two-component system response regulator |
| SHJG6020 | 6864626 | 6863148 | 1479bp | two-component system sensor kinase |
| SHJG6021 | 6865429 | 6864734 | 696bp | two-component system response regulator |
| SHJG6114 | 6963754 | 6965289 | 1536bp | putative two-component system sensor kinase |
| SHJG6115 | 6965300 | 6966061 | 762bp | putative two-component system response regulator |
| SHJG6231 | 7094394 | 7095587 | 1194bp | two-component system sensor kinase |
| SHJG6232 | 7095584 | 7096198 | 615bp | two-component system response regulator |
| SHJG6391 | 7271909 | 7270854 | 1056bp | two-component system sensor kinase |
| SHJG6392 | 7272715 | 7271975 | 741bp | two-component system response regulator |
| SHJG6458 | 7350103 | 7349462 | 642bp | chitinase two-component response regulator |
| SHJG6459 | 7351272 | 7350100 | 1173bp | chitinase two-component sensor kinase |
| SHJG6510 | 7405410 | 7404715 | 696bp | two-component response regulator |
| SHJG6511 | 7407026 | 7405407 | 1620bp | two-component sensor kinase |
| SHJG6836 | 7783809 | 7789184 | 5376bp | two-component system sensor kinase |
| SHJG6837 | 7789479 | 7790159 | 681bp | two-component system response regulator |
| SHJG6867 | 7823553 | 7824353 | 801bp | two-component system response regulator |
| SHJG6869 | 7824363 | 7825793 | 1431bp | two-component system sensor kinase |
| SHJG6925 | 7891183 | 7892259 | 1077bp | two-component system sensor kinase |
| SHJG6926 | 7892256 | 7892945 | 690bp | two-component system response regulator |
| SHJG6934 | 7899514 | 7898834 | 681bp | two-component system response regulator |
| SHJG6935 | 7901139 | 7899511 | 1629bp | two-component system sensor kinase |
| SHJG6960 | 7930032 | 7930685 | 654bp | two-component system response regulator |
| SHJG6961 | 7930691 | 7931947 | 1257bp | two-component system sensor kinase |
| SHJG6969 | 7937206 | 7939764 | 2559bp | two-component system sensor kinase |
| SHJG6970 | 7939800 | 7940477 | 678bp | putative two-component system response regulator |
| SHJG7492 | 8522752 | 8521976 | 777bp | two-component system response regulator |
| SHJG7495 | 8523737 | 8525458 | 1722bp | two-component system sensor kinase |
| SHJG7733 | 8793039 | 8793827 | 789bp | putative two-component system response regulator |
| SHJG7736 | 8796144 | 8797361 | 1218bp | putative two-component system sensor kinase |
| SHJG7991 | 9132162 | 9132836 | 675bp | two-component system response regulator |
| SHJG7992 | 9132838 | 9133947 | 1110bp | two-component system sensor kinase |
| SHJG8262 | 9472914 | 9474119 | 1206bp | two-component system sensor kinase |
| SHJG8263 | 9474116 | 9474769 | 654bp | two-component system response regulator |
| SHJG8528 | 9754629 | 9755582 | 954bp | two-component system sensor kinase |
| SHJG8529 | 9755585 | 9756286 | 702bp | two-component system response regulator |
| SHJG8601 | 9835492 | 9836553 | 1062bp | two-component system sensor kinase |
| SHJG8602 | 9836553 | 9837212 | 660bp | two-component system response regulator |
| SHJG5089 | 5829553 | 5834742 | 5190bp | two-component system sensor kinase/response regulator, bifunctional protein |
| SHJG0512 | 652492 | 651833 | 660bp | two-component system response regulator |
| SHJG1129 | 1352614 | 1351718 | 897bp | two-component system response regulator |
| SHJG1490 | 1772297 | 1772977 | 681bp | two-component system response regulator |
| SHJG3092 | 3632558 | 3633229 | 672bp | two-component system response regulator |
| SHJG3485 | 4055133 | 4054477 | 657bp | two-component system response regulator |
| SHJG3632 | 4217323 | 4217724 | 402bp | two-component system response regulator |
| SHJG4472 | 5152253 | 5151510 | 744bp | two-component system response regulator |
| SHJG4605 | 5307828 | 5307061 | 768bp | two-component system response regulator |
| SHJG4614 | 5315753 | 5315082 | 672bp | two-component system response regulator |
| SHJG4923 | 5644349 | 5645128 | 780bp | two-component system response regulator |
| SHJG5865 | 6670285 | 6670827 | 543bp | two-component system response regulator |
| SHJG6929 | 7895858 | 7895166 | 693bp | two-component system response regulator |
| SHJG7094 | 8103829 | 8103170 | 660bp | two-component system response regulator |
| SHJG8584 | 9819360 | 9819154 | 207bp | two-component system response regulator |
| SHJG8626 | 9860156 | 9859536 | 621bp | two-component system response regulator |
| SHJG0091 | 121209 | 120880 | 330bp | two-component system sensor kinase |
| SHJG0521 | 663265 | 661868 | 1398bp | two-component system sensor kinase |
| SHJG0640 | 804426 | 805751 | 1326bp | two-component system sensor kinase |
| SHJG1062 | 1264498 | 1265478 | 981bp | two-component system sensor kinase |
| SHJG1480 | 1761207 | 1759468 | 1740bp | two-component system sensor kinase |
| SHJG2006 | 2463705 | 2459443 | 4263bp | two-component system sensor kinase |
| SHJG2130 | 2590179 | 2591630 | 1452bp | two-component system sensor kinase |
| SHJG2400 | 2888174 | 2885679 | 2496bp | two-component system sensor kinase |
| SHJG3034 | 3576106 | 3574976 | 1131bp | two-component system sensor kinase |
| SHJG4592 | 5294569 | 5293331 | 1239bp | two-component system sensor kinase |
| SHJG6350 | 7226244 | 7227710 | 1467bp | two-component system sensor kinase |
| SHJG6413 | 7297933 | 7296689 | 1245bp | two-component system sensor kinase |
| SHJG8801 | 10053708 | 10055426 | 1719bp | two-component system sensor kinase |

**⑵ Transcriptional regulators**

| **ID** | **Start** | **End** | **Size** | **Function** |
| --- | --- | --- | --- | --- |
| SHJG0024 | 29155 | 28589 | 567bp | TetR-family transcriptional regulator |
| SHJG0043 | 47955 | 48530 | 576bp | TetR-family transcriptional regulator |
| SHJG0116 | 154550 | 155086 | 537bp | TetR-family transcriptional regulator |
| SHJG0160 | 221819 | 221163 | 657bp | TetR-family transcriptional regulator |
| SHJG0186 | 251637 | 252245 | 609bp | TetR-family transcriptional regulator |
| SHJG0395 | 528146 | 527574 | 573bp | TetR-family transcriptional regulator |
| SHJG0408 | 540088 | 539600 | 489bp | TetR-family transcriptional regulator |
| SHJG0417 | 549544 | 548954 | 591bp | TetR-family transcriptional regulator |
| SHJG0427 | 560264 | 559776 | 489bp | TetR-family transcriptional regulator |
| SHJG0431 | 564642 | 565262 | 621bp | TetR-family transcriptional regulator |
| SHJG0472 | 608406 | 607879 | 528bp | TetR-family transcriptional regulator |
| SHJG0491 | 629780 | 630541 | 762bp | TetR-family transcriptional regulator |
| SHJG0542 | 689335 | 688649 | 687bp | TetR-family transcriptional regulator |
| SHJG0547 | 694154 | 693405 | 750bp | TetR-family transcriptional regulator |
| SHJG0567 | 713098 | 713703 | 606bp | TetR-family transcriptional regulator |
| SHJG0652 | 818689 | 819336 | 648bp | TetR-family transcriptional regulator |
| SHJG0672 | 842227 | 842823 | 597bp | TetR-family transcriptional regulator |
| SHJG0678 | 848830 | 849408 | 579bp | TetR-family transcriptional regulator |
| SHJG0763 | 941540 | 940983 | 558bp | TetR-family transcriptional regulator |
| SHJG0769 | 946862 | 947425 | 564bp | TetR-family transcriptional regulator |
| SHJG0770 | 948134 | 947565 | 570bp | TetR-family transcriptional regulator |
| SHJG0783 | 959270 | 958602 | 669bp | TetR-family transcriptional regulator |
| SHJG0787 | 963065 | 962439 | 627bp | TetR-family transcriptional regulator |
| SHJG0820 | 1004429 | 1005073 | 645bp | TetR-family transcriptional regulator |
| SHJG0887 | 1067401 | 1068015 | 615bp | putative TetR-family transcriptional regulator |
| SHJG0942 | 1127002 | 1127604 | 603bp | TetR-family transcriptional regulator |
| SHJG0951 | 1136460 | 1136975 | 516bp | TetR-family transcriptional regulator |
| SHJG1026 | 1215981 | 1216577 | 597bp | TetR-family transcriptional regulator |
| SHJG1109 | 1327136 | 1327573 | 438bp | TetR-family transcriptional regulator |
| SHJG1138 | 1373132 | 1373713 | 582bp | TetR-family transcriptional regulator |
| SHJG1144 | 1378710 | 1379297 | 588bp | TetR-family transcriptional regulator |
| SHJG1147 | 1381714 | 1382295 | 582bp | TetR-family transcriptional regulator |
| SHJG1152 | 1387476 | 1386877 | 600bp | TetR-family transcriptional regulator |
| SHJG1159 | 1393650 | 1394171 | 522bp | putative TetR-family transcriptional regulator |
| SHJG1160 | 1395020 | 1394409 | 612bp | TetR-family transcriptional regulator |
| SHJG1168 | 1404814 | 1404272 | 543bp | TetR-family transcriptional regulator |
| SHJG1172 | 1408671 | 1408054 | 618bp | TetR-family transcriptional regulator |
| SHJG1198 | 1436049 | 1435480 | 570bp | TetR-family transcriptional regulator |
| SHJG1210 | 1449584 | 1450189 | 606bp | TetR-family transcriptional regulator |
| SHJG1215 | 1454904 | 1455596 | 693bp | TetR-family transcriptional regulator |
| SHJG1236 | 1482689 | 1482120 | 570bp | TetR-family transcriptional regulator |
| SHJG1244 | 1490524 | 1489841 | 684bp | TetR-family transcriptional regulator |
| SHJG1307 | 1566107 | 1565631 | 477bp | TetR-family transcriptional regulator |
| SHJG1321 | 1578653 | 1578075 | 579bp | TetR-family transcriptional regulator |
| SHJG1325 | 1581276 | 1581824 | 549bp | TetR-family transcriptional regulator |
| SHJG1334 | 1591971 | 1591306 | 666bp | Shy4-TetR-family transcriptional regulator |
| SHJG1626 | 1949179 | 1949766 | 588bp | TetR-family transcriptional regulator |
| SHJG1763 | 2136366 | 2136965 | 600bp | TetR-family transcriptional regulator |
| SHJG1782 | 2156987 | 2156373 | 615bp | putative TetR-family transcriptional regulator |
| SHJG1982 | 2433706 | 2433149 | 558bp | putative TetR-family transcriptional regulator |
| SHJG2037 | 2493553 | 2494128 | 576bp | putative TetR-family transcriptional regulator |
| SHJG2085 | 2542792 | 2542217 | 576bp | putative TetR-family transcriptional regulator |
| SHJG2123 | 2584257 | 2583637 | 621bp | TetR-family transcriptional regulator |
| SHJG2153 | 2617027 | 2616398 | 630bp | TetR-family transcriptional regulator |
| SHJG2500 | 3005889 | 3006479 | 591bp | TetR-family transcriptional regulator |
| SHJG2519 | 3024550 | 3023858 | 693bp | putative TetR-family transcriptional regulator |
| SHJG2555 | 3057159 | 3057827 | 669bp | TetR-family transcriptional regulator |
| SHJG2649 | 3162901 | 3163545 | 645bp | TetR-family transcriptional regulator |
| SHJG2864 | 3396712 | 3396065 | 648bp | TetR-family transcriptional regulator |
| SHJG2992 | 3532762 | 3532211 | 552bp | TetR-family transcriptional regulator |
| SHJG3137 | 3680607 | 3680005 | 603bp | TetR-family transcriptional regulator |
| SHJG3149 | 3693738 | 3693103 | 636bp | TetR-family transcriptional regulator |
| SHJG3152 | 3695415 | 3696050 | 636bp | TetR-family transcriptional regulator |
| SHJG3161 | 3706989 | 3706372 | 618bp | TetR-family transcriptional regulator |
| SHJG3354 | 3912657 | 3912085 | 573bp | TetR-family transcriptional regulator |
| SHJG3365 | 3924280 | 3923747 | 534bp | TetR-family transcriptional regulator |
| SHJG3499 | 4071688 | 4071101 | 588bp | TetR-family transcriptional regulator |
| SHJG3582 | 4165877 | 4166506 | 630bp | TetR-family transcriptional regulator |
| SHJG3710 | 4299151 | 4299816 | 666bp | TetR-family transcriptional regulator |
| SHJG3743 | 4340102 | 4339539 | 564bp | TetR-family transcriptional regulator |
| SHJG3767 | 4362592 | 4361978 | 615bp | TetR-family transcriptional regulator |
| SHJG3811 | 4406263 | 4406880 | 618bp | TetR-family transcriptional regulator |
| SHJG3868 | 4465311 | 4465937 | 627bp | TetR-family transcriptional regulator |
| SHJG3961 | 4569536 | 4570123 | 588bp | TetR-family transcriptional regulator |
| SHJG4236 | 4875160 | 4875963 | 804bp | putative TetR-family transcriptional regulator |
| SHJG4276 | 4925181 | 4924585 | 597bp | TetR-family transcriptional regulator |
| SHJG4457 | 5129272 | 5128634 | 639bp | TetR-family transcriptional regulator |
| SHJG4601 | 5304655 | 5305332 | 678bp | TetR-family transcriptional regulator |
| SHJG4638 | 5344197 | 5343592 | 606bp | TetR-family transcriptional regulator |
| SHJG4673 | 5382298 | 5381690 | 609bp | TetR-family transcriptional regulator |
| SHJG4680 | 5389791 | 5389144 | 648bp | TetR-family transcriptional regulator |
| SHJG4726 | 5444703 | 5445338 | 636bp | TetR-family transcriptional regulator |
| SHJG4757 | 5475073 | 5474468 | 606bp | TetR-family transcriptional regulator |
| SHJG4898 | 5623838 | 5623221 | 618bp | TetR-family transcriptional regulator |
| SHJG4951 | 5673124 | 5673726 | 603bp | TetR-family transcriptional regulator |
| SHJG4963 | 5693625 | 5694401 | 777bp | TetR-family transcriptional regulator |
| SHJG4979 | 5716940 | 5717626 | 687bp | TetR-family transcriptional regulator |
| SHJG5058 | 5797745 | 5797023 | 723bp | putative TetR-family transcriptional regulator |
| SHJG5090 | 5835333 | 5834761 | 573bp | TetR-family transcriptional regulator |
| SHJG5241 | 6003287 | 6003874 | 588bp | TetR-family transcriptional regulator |
| SHJG5568 | 6356256 | 6355657 | 600bp | TetR-family transcriptional regulator |
| SHJG5582 | 6371876 | 6372511 | 636bp | TetR-family transcriptional regulator |
| SHJG5625 | 6429873 | 6429289 | 585bp | TetR-family transcriptional regulator |
| SHJG5677 | 6486403 | 6487017 | 615bp | TetR-family transcriptional regulator |
| SHJG5766 | 6577280 | 6577840 | 561bp | TetR-family transcriptional regulator |
| SHJG5771 | 6581848 | 6582471 | 624bp | TetR-family transcriptional regulator |
| SHJG5963 | 6804772 | 6804191 | 582bp | TetR-family transcriptional regulator |
| SHJG5975 | 6817952 | 6817206 | 747bp | TetR-family transcriptional regulator |
| SHJG6077 | 6923530 | 6922898 | 633bp | TetR-family transcriptional regulator |
| SHJG6272 | 7135795 | 7135229 | 567bp | TetR-family transcriptional regulator |
| SHJG6319 | 7195392 | 7195649 | 258bp | TetR-family transcriptional regulator |
| SHJG6320 | 7195674 | 7195997 | 324bp | TetR-family transcriptional regulator |
| SHJG6349 | 7225281 | 7225910 | 630bp | TetR-family transcriptional regulator |
| SHJG6407 | 7290493 | 7289843 | 651bp | TetR-family transcriptional regulator |
| SHJG6490 | 7381889 | 7382539 | 651bp | TetR-family transcriptional regulator |
| SHJG6494 | 7386273 | 7385632 | 642bp | TetR-family transcriptional regulator |
| SHJG6575 | 7485764 | 7485138 | 627bp | TetR-family transcriptional regulator |
| SHJG6588 | 7498806 | 7498207 | 600bp | TetR-family transcriptional regulator |
| SHJG6614 | 7526036 | 7526620 | 585bp | TetR-family transcriptional regulator |
| SHJG6629 | 7542180 | 7542788 | 609bp | TetR-family transcriptional regulator |
| SHJG6726 | 7649093 | 7649650 | 558bp | TetR-family transcriptional regulator |
| SHJG6910 | 7868706 | 7869266 | 561bp | TetR-family transcriptional regulator |
| SHJG7067 | 8061273 | 8061872 | 600bp | putative TetR-family transcriptional regulator |
| SHJG7212 | 8228957 | 8228382 | 576bp | TetR-family transcriptional regulator |
| SHJG7318 | 8349078 | 8348398 | 681bp | TetR-family transcriptional regulator |
| SHJG7322 | 8352840 | 8352223 | 618bp | TetR-family transcriptional regulator |
| SHJG7435 | 8469339 | 8468614 | 726bp | TetR-family transcriptional regulator |
| SHJG7562 | 8606215 | 8605631 | 585bp | TetR-family transcriptional regulator |
| SHJG7565 | 8607733 | 8608323 | 591bp | TetR-family transcriptional regulator |
| SHJG7567 | 8611106 | 8610441 | 666bp | putative TetR-family transcriptional regulator |
| SHJG7600 | 8650804 | 8651418 | 615bp | TetR-family transcriptional regulator |
| SHJG7665 | 8725533 | 8724922 | 612bp | TetR-family transcriptional regulator |
| SHJG7731 | 8791068 | 8790490 | 579bp | putative TetR-family transcriptional regulator |
| SHJG7793 | 8861259 | 8861879 | 621bp | TetR-family transcriptional regulator |
| SHJG7908 | 8999646 | 9000251 | 606bp | putative TetR-family transcriptional regulator |
| SHJG7910 | 9002755 | 9002889 | 135bp | TetR-family transcriptional regulator |
| SHJG7911 | 9002795 | 9002079 | 717bp | TetR-family transcriptional regulator |
| SHJG8023 | 9170987 | 9171559 | 573bp | TetR-family transcriptional regulator |
| SHJG8046 | 9196215 | 9196823 | 609bp | TetR-family transcriptional regulator |
| SHJG8150 | 9347974 | 9348636 | 663bp | TetR-family transcriptional regulator |
| SHJG8312 | 9520296 | 9519685 | 612bp | TetR-family transcriptional regulator |
| SHJG8331 | 9541222 | 9541788 | 567bp | putative TetR-family transcriptional regulator |
| SHJG8401 | 9625406 | 9624738 | 669bp | TetR-family transcriptional regulator |
| SHJG8654 | 9892212 | 9892673 | 462bp | putative TetR-family transcriptional regulator |
| SHJG8684 | 9927253 | 9927900 | 648bp | TetR-family transcriptional regulator |
| SHJG8694 | 9942515 | 9941883 | 633bp | TetR-family transcriptional regulator |
| SHJG0021 | 27077 | 26556 | 522bp | MarR-family transcriptional regulator |
| SHJG0196 | 264930 | 264457 | 474bp | MarR-family transcriptional regulator |
| SHJG0662 | 827989 | 828348 | 360bp | putative MarR-family transcriptional regulator |
| SHJG0663 | 828785 | 828357 | 429bp | MarR-family transcriptional regulator |
| SHJG0759 | 935871 | 935440 | 432bp | MarR-family transcriptional regulator |
| SHJG0781 | 957658 | 957167 | 492bp | putative MarR-family transcriptional regulator |
| SHJG0866 | 1048561 | 1048091 | 471bp | MarR-family transcriptional regulator |
| SHJG0950 | 1135743 | 1136237 | 495bp | MarR-family transcriptional regulator |
| SHJG0961 | 1146732 | 1147205 | 474bp | MarR-family transcriptional regulator |
| SHJG1005 | 1192004 | 1191606 | 399bp | MarR-family transcriptional regulator |
| SHJG1224 | 1465324 | 1464860 | 465bp | MarR-family transcriptional regulator |
| SHJG1477 | 1757762 | 1758208 | 447bp | MarR-family transcriptional regulator |
| SHJG1617 | 1939385 | 1938891 | 495bp | MarR-family transcriptional regulator |
| SHJG1684 | 2050563 | 2049970 | 594bp | putative MarR-family transcriptional regulator |
| SHJG1778 | 2151804 | 2152376 | 573bp | MarR-family transcriptional regulator |
| SHJG2055 | 2511584 | 2511102 | 483bp | putative MarR-family transcriptional regulator |
| SHJG2091 | 2547983 | 2547528 | 456bp | MarR-family transcriptional regulator |
| SHJG2286 | 2760228 | 2760668 | 441bp | putative MarR-family transcriptional regulator |
| SHJG2319 | 2796022 | 2796456 | 435bp | putative MarR-family transcriptional regulator |
| SHJG2513 | 3018848 | 3018447 | 402bp | MarR-family transcriptional regulator |
| SHJG2550 | 3052440 | 3051847 | 594bp | MarR-family transcriptional regulator |
| SHJG2587 | 3096055 | 3096504 | 450bp | MarR-family transcriptional regulator |
| SHJG3886 | 4481837 | 4481400 | 438bp | MarR-family transcriptonal regulator |
| SHJG3998 | 4609206 | 4609628 | 423bp | putative MarR-family transcriptional regulator |
| SHJG4148 | 4775437 | 4775937 | 501bp | MarR-family transcriptional regulator |
| SHJG4233 | 4871382 | 4871582 | 201bp | MarR-family transcriptional regulator |
| SHJG4256 | 4899453 | 4900379 | 927bp | MarR-family transcriptional regulator |
| SHJG4604 | 5306605 | 5307102 | 498bp | MarR-family transcriptional regulator |
| SHJG4677 | 5385517 | 5386029 | 513bp | MarR-family transcriptional regulator |
| SHJG4782 | 5503927 | 5503547 | 381bp | MarR-family transcriptional regulator |
| SHJG4929 | 5650429 | 5649941 | 489bp | MarR-family transcriptional regulator |
| SHJG4959 | 5688458 | 5687910 | 549bp | MarR-family transcriptional regulator |
| SHJG4975 | 5713332 | 5713859 | 528bp | putative MarR-family transcriptional regulator |
| SHJG5054 | 5792457 | 5792951 | 495bp | MarR-family transcriptional regulator |
| SHJG5067 | 5807990 | 5808481 | 492bp | MarR-family transcriptional regulator |
| SHJG5158 | 5906750 | 5907208 | 459bp | MarR-family transcriptional regulator |
| SHJG5316 | 6089753 | 6089415 | 339bp | MarR-family transcriptional regulator |
| SHJG6066 | 6913267 | 6912767 | 501bp | MarR-family transcriptional regulator |
| SHJG6398 | 7280836 | 7281327 | 492bp | MarR-family transcriptional regulator |
| SHJG6482 | 7377316 | 7376840 | 477bp | MarR-family transcriptional regulator |
| SHJG6491 | 7382915 | 7383421 | 507bp | MarR-family transcriptional regulator |
| SHJG6872 | 7827984 | 7827541 | 444bp | MarR-family transcriptional regulator |
| SHJG7011 | 7996738 | 7996241 | 498bp | MarR-family transcriptional regulator |
| SHJG7511 | 8547845 | 8548282 | 438bp | MarR-family transcriptional regulator |
| SHJG7608 | 8660487 | 8660951 | 465bp | MarR-family transcriptional regulator |
| SHJG8138 | 9329716 | 9329279 | 438bp | putative MarR-family regulatory protein |
| SHJG8402 | 9625565 | 9626065 | 501bp | MarR-family transcriptional regulator |
| SHJG8404 | 9627466 | 9627900 | 435bp | MarR-family transcriptional regulator |
| SHJG0438 | 572760 | 571855 | 906bp | LysR-family transcriptional regulator |
| SHJG0645 | 812329 | 813309 | 981bp | LysR-family transcriptional regulator |
| SHJG0710 | 881945 | 881010 | 936bp | putative LysR-family transcriptional regulator |
| SHJG0796 | 970331 | 969321 | 1011bp | LysR-family transcriptional regulator |
| SHJG0802 | 980780 | 979794 | 987bp | putative LysR-family transcriptional regulator |
| SHJG0847 | 1025425 | 1026351 | 927bp | LysR-family transcriptional regulator |
| SHJG0849 | 1030023 | 1027333 | 2691bp | LysR-family transcriptional regulator |
| SHJG0858 | 1039661 | 1042384 | 2724bp | LysR-family transcriptional regulator |
| SHJG0900 | 1081723 | 1082604 | 882bp | LysR-family transcriptional regulator |
| SHJG0909 | 1089890 | 1088976 | 915bp | LysR-family transcriptional regulator |
| SHJG1093 | 1312016 | 1311135 | 882bp | LysR-family transcriptional regulator |
| SHJG1098 | 1316605 | 1317546 | 942bp | LysR-family transcriptional regulator |
| SHJG1122 | 1340801 | 1341706 | 906bp | LysR-family transcriptional regulator |
| SHJG1151 | 1385631 | 1386581 | 951bp | LysR-family transcriptional regulator |
| SHJG1213 | 1452736 | 1453704 | 969bp | LysR-family transcriptional regulator |
| SHJG1300 | 1559398 | 1559156 | 243bp | LysR-family transcriptional regulator |
| SHJG1410 | 1690449 | 1690168 | 282bp | LysR-family transcriptional regulator |
| SHJG1571 | 1879301 | 1880212 | 912bp | LysR-family transcription regulator |
| SHJG1696 | 2060812 | 2061648 | 837bp | LysR-family transcriptional regulator |
| SHJG1757 | 2131985 | 2131104 | 882bp | LysR-family transcriptional regulator |
| SHJG1771 | 2144175 | 2143249 | 927bp | putative LysR-family transcriptional regulator |
| SHJG1871 | 2266231 | 2267127 | 897bp | LysR-family transcriptional regulator |
| SHJG1900 | 2303268 | 2302357 | 912bp | LysR-family transcriptional regulator |
| SHJG1958 | 2411599 | 2412069 | 471bp | LysR-family transcriptional regulator |
| SHJG2114 | 2574172 | 2575059 | 888bp | putative LysR-family transcriptional regulator |
| SHJG2140 | 2604558 | 2603596 | 963bp | LysR-family transcriptional regulator |
| SHJG2167 | 2629165 | 2630073 | 909bp | LysR-family transcriptional regulator |
| SHJG2351 | 2827843 | 2826914 | 930bp | putative LysR-family transcriptional regulator |
| SHJG2363 | 2840759 | 2841628 | 870bp | LysR-family transcriptional regulator |
| SHJG2576 | 3086632 | 3085868 | 765bp | LysR-family trancsriptional regulator |
| SHJG2773 | 3282171 | 3281269 | 903bp | LysR-family transcriptional regulator |
| SHJG3052 | 3592906 | 3591989 | 918bp | LysR-family transcriptional regulator |
| SHJG4434 | 5096647 | 5095670 | 978bp | LysR-family transcriptional regulator |
| SHJG5151 | 5900221 | 5901126 | 906bp | LysR-family transcriptional regulator |
| SHJG5680 | 6490497 | 6491357 | 861bp | LysR-family transcriptional regulator |
| SHJG5863 | 6668441 | 6669346 | 906bp | LysR-family transcriptional regulator |
| SHJG6013 | 6857566 | 6858462 | 897bp | LysR-family transcriptional regulator |
| SHJG6130 | 6978108 | 6978992 | 885bp | hydrogen peroxide sensing regulator, LysR-family |
| SHJG7158 | 8170784 | 8171710 | 927bp | LysR-family transcriptional regulator |
| SHJG7508 | 8543643 | 8542774 | 870bp | LysR-family transcriptional regulator |
| SHJG7676 | 8736038 | 8736940 | 903bp | LysR-family transcriptional regulator |
| SHJG7790 | 8860454 | 8859531 | 924bp | LysR-family transcriptional regulator |
| SHJG7879 | 8973118 | 8972234 | 885bp | LysR-family transcriptional regulator |
| SHJG8016 | 9164509 | 9165723 | 1215bp | LysR-family transcriptional regulator |
| SHJG8265 | 9476950 | 9475973 | 978bp | LysR-family transcriptional regulator |
| SHJG8399 | 9623454 | 9622555 | 900bp | LysR-family transcriptional regulator |
| SHJG8420 | 9641075 | 9642076 | 1002bp | LysR-family transcriptional regulator |
| SHJG8455 | 9684795 | 9683875 | 921bp | putative LysR-family transcriptional regulator |
| SHJG8582 | 9815217 | 9816134 | 918bp | putative LysR-family transcriptional regulator |
| SHJG8860 | 10133256 | 10134167 | 912bp | LysR-family transcription regulator |
| SHJG0975 | 1159988 | 1159233 | 756bp | putative GntR-family transcriptional regulator |
| SHJG1053 | 1254967 | 1254278 | 690bp | GntR-family transcriptional regulator |
| SHJG1231 | 1474618 | 1475268 | 651bp | GntR-family transcriptional regulator |
| SHJG1283 | 1541758 | 1542609 | 852bp | GntR-family transcriptional regulator |
| SHJG1715 | 2081102 | 2082190 | 1089bp | putative GntR-family transcriptional regulator |
| SHJG2293 | 2766749 | 2767492 | 744bp | putative GntR-family transcriptional regulator |
| SHJG2700 | 3208696 | 3207935 | 762bp | GntR-family transcriptional regulator |
| SHJG2775 | 3284463 | 3283240 | 1224bp | GntR-family transcriptional regulator |
| SHJG2838 | 3369707 | 3369006 | 702bp | GntR-family transcriptional regulator |
| SHJG2852 | 3384219 | 3382711 | 1509bp | GntR-family transcriptional regulator |
| SHJG3050 | 3590471 | 3591181 | 711bp | GntR-family transcriptional regulator |
| SHJG3101 | 3645364 | 3646035 | 672bp | GntR-family transcriptional regulator |
| SHJG3122 | 3667616 | 3666915 | 702bp | GntR-family transcriptional regulator |
| SHJG3164 | 3709154 | 3709900 | 747bp | GntR-family transcriptional regulator |
| SHJG3180 | 3725608 | 3725237 | 372bp | GntR-family transcriptional regulator |
| SHJG3263 | 3819381 | 3820073 | 693bp | GntR-family transcriptional regulator |
| SHJG3339 | 3898973 | 3898581 | 393bp | GntR-family transcriptional regulator |
| SHJG3442 | 4007297 | 4008733 | 1437bp | GntR-family transcriptional regulator |
| SHJG3457 | 4022946 | 4023647 | 702bp | GntR-family transcriptional regulator |
| SHJG3661 | 4251955 | 4252578 | 624bp | GntR-family transcriptional regulator |
| SHJG4334 | 4989097 | 4989786 | 690bp | GntR-family transcriptional regulator |
| SHJG4901 | 5627969 | 5627118 | 852bp | GntR-family transcriptional regulator |
| SHJG4903 | 5629272 | 5628358 | 915bp | GntR-family transcriptional regulator |
| SHJG5077 | 5820867 | 5821655 | 789bp | GntR-family transcriptional regulator |
| SHJG5108 | 5852777 | 5854174 | 1398bp | GntR-family transcriptional regulator |
| SHJG5259 | 6024761 | 6024414 | 348bp | GntR-family transcriptional regulator |
| SHJG5261 | 6025944 | 6026624 | 681bp | GntR-family transcriptional regulator |
| SHJG5327 | 6102244 | 6101402 | 843bp | GntR-family transcriptional regulator |
| SHJG5400 | 6178924 | 6178229 | 696bp | GntR-family transcriptional regulator |
| SHJG5959 | 6800027 | 6801457 | 1431bp | GntR-family transcriptional regulator |
| SHJG6195 | 7049508 | 7050260 | 753bp | GntR-family transcriptional regulator |
| SHJG6346 | 7223190 | 7222429 | 762bp | GntR-family transcriptional regulator |
| SHJG6550 | 7457130 | 7457822 | 693bp | GntR-family transcriptional regulator |
| SHJG6944 | 7913346 | 7914032 | 687bp | GntR-family transcriptional regulator |
| SHJG7049 | 8038336 | 8037518 | 819bp | GntR-family transcriptional regulator |
| SHJG7233 | 8254134 | 8254922 | 789bp | GntR-family transcriptional regulator |
| SHJG7307 | 8335186 | 8335923 | 738bp | GntR-family transcriptional regulator |
| SHJG7521 | 8558355 | 8559032 | 678bp | GntR-family transcriptional regulator |
| SHJG7717 | 8778426 | 8777695 | 732bp | putative GntR-family regulatory protein |
| SHJG7738 | 8799690 | 8798992 | 699bp | GntR-family transcriptional regulator |
| SHJG7849 | 8940364 | 8939633 | 732bp | GntR-family transcriptional regulator |
| SHJG8299 | 9508365 | 9509105 | 741bp | GntR-family transcriptional regulator |
| SHJG0493 | 632247 | 631285 | 963bp | AraC-family transcriptional regulator |
| SHJG0690 | 866504 | 865596 | 909bp | putative AraC-family transcriptional regulator |
| SHJG0962 | 1148166 | 1147270 | 897bp | AraC-family transcriptional regulator |
| SHJG1271 | 1532297 | 1532752 | 456bp | AraC-family transcriptional regulator |
| SHJG1313 | 1570390 | 1571202 | 813bp | Shy25-AraC-family transcriptional regulator |
| SHJG1358 | 1619083 | 1618178 | 906bp | Shy25-AraC-family transcriptional regulator |
| SHJG1419 | 1696958 | 1697821 | 864bp | AraC-family transcriptional regulator |
| SHJG1423 | 1700072 | 1700677 | 606bp | AraC-family transcriptional regulator |
| SHJG1554 | 1851152 | 1851670 | 519bp | AraC-family transcriptional regulator |
| SHJG1609 | 1932412 | 1931978 | 435bp | AraC-family transcriptional regulator |
| SHJG1824 | 2206150 | 2207070 | 921bp | putative AraC-family transcriptional regulator |
| SHJG1839 | 2221486 | 2222460 | 975bp | AraC-family transcriptional regulator |
| SHJG1858 | 2250311 | 2249325 | 987bp | AraC-family transcriptional regulator |
| SHJG1880 | 2277295 | 2276456 | 840bp | AraC-family transcriptional regulator |
| SHJG1932 | 2386729 | 2385779 | 951bp | AraC-family transcriptional regulator |
| SHJG2141 | 2604760 | 2605698 | 939bp | AraC-family transcriptional regulator |
| SHJG2359 | 2835796 | 2836776 | 981bp | AraC-family transcriptional regulator |
| SHJG3494 | 4066968 | 4066078 | 891bp | putative AraC-family transcriptional regulator |
| SHJG3785 | 4381219 | 4380371 | 849bp | AraC-family transcriptional regulator |
| SHJG4361 | 5020490 | 5019672 | 819bp | AraC-family transcriptional regulator |
| SHJG4735 | 5451127 | 5452155 | 1029bp | AraC-family transcriptional regulator |
| SHJG4885 | 5607910 | 5608707 | 798bp | AraC-family transcriptional regulator |
| SHJG5267 | 6031055 | 6030390 | 666bp | AraC-family transcriptional regulator |
| SHJG6337 | 7211249 | 7210287 | 963bp | AraC-family transcriptional regulator |
| SHJG6367 | 7240553 | 7240152 | 402bp | AraC-family transcriptional regulator |
| SHJG6551 | 7458851 | 7457934 | 918bp | AraC-family transcriptional regulator |
| SHJG6849 | 7805441 | 7806334 | 894bp | AraC-family transcriptional regulator |
| SHJG7196 | 8212918 | 8213511 | 594bp | AraC-family transcriptional regulator |
| SHJG7578 | 8624729 | 8625619 | 891bp | AraC-family transcriptional regulator |
| SHJG8076 | 9224548 | 9225504 | 957bp | AraC-family transcriptional regulator |
| SHJG8244 | 9453580 | 9452636 | 945bp | AraC-family transcriptional regulator |
| SHJG8344 | 9554249 | 9553308 | 942bp | AraC-family transcriptional regulator |
| SHJG8509 | 9740551 | 9739709 | 843bp | AraC-family transcriptional regulator |
| SHJG8510 | 9740769 | 9741380 | 612bp | AraC-family transcriptional regulator |
| SHJG8540 | 9771519 | 9770650 | 870bp | putative AraC-family regulator |
| SHJG8562 | 9792686 | 9793195 | 510bp | AraC-family transcriptional regulator |
| SHJG8839 | 10102125 | 10100218 | 1908bp | AraC-family transcriptional regulator |
| SHJG0081 | 111681 | 108895 | 2787bp | LuxR-family transcriptional regulator |
| SHJG0173 | 238021 | 235262 | 2760bp | LuxR-family transcriptional regulator |
| SHJG0337 | 444804 | 447593 | 2790bp | LuxR-family transcriptional regulator |
| SHJG0393 | 526612 | 523844 | 2769bp | LuxR-family transcriptional regulator |
| SHJG0411 | 545418 | 542626 | 2793bp | LuxR-family transcriptional regulator |
| SHJG0426 | 559185 | 556432 | 2754bp | LuxR-family transcriptional regulator |
| SHJG0435 | 567167 | 569956 | 2790bp | LuxR-family transcriptional regulator |
| SHJG0453 | 591287 | 588528 | 2760bp | LuxR-family transcriptional regulator |
| SHJG0474 | 609267 | 612314 | 3048bp | LuxR-family transcriptional regulator |
| SHJG0571 | 717970 | 716930 | 1041bp | LuxR-family transcriptional regulator |
| SHJG0805 | 983091 | 985820 | 2730bp | LuxR-family transcriptional regulator |
| SHJG0815 | 996917 | 994356 | 2562bp | LuxR-family transcriptional regulator |
| SHJG1067 | 1274669 | 1272099 | 2571bp | LuxR-family transcriptional regulator |
| SHJG1068 | 1277590 | 1274825 | 2766bp | LuxR-family transcriptional regulator |
| SHJG1230 | 1474262 | 1471566 | 2697bp | LuxR-family regulatory protein |
| SHJG1268 | 1528942 | 1528205 | 738bp | putative LuxR-family transcriptional regulator |
| SHJG1401 | 1681942 | 1681289 | 654bp | LuxR-family transcriptional regulator |
| SHJG1659 | 1994563 | 1997382 | 2820bp | LuxR-family transcriptional regulator |
| SHJG1728 | 2097045 | 2094721 | 2325bp | possible LuxR-family transcriptional regulator |
| SHJG1906 | 2311056 | 2313566 | 2511bp | LuxR-family transcriptional regulator |
| SHJG2768 | 3277645 | 3275069 | 2577bp | LuxR-family transcriptional regulator |
| SHJG5375 | 6151503 | 6150655 | 849bp | LuxR-family transcriptional regulator |
| SHJG5424 | 6209245 | 6206000 | 3246bp | LuxR-family transcriptional regulator |
| SHJG5752 | 6566724 | 6564298 | 2427bp | LuxR-family transcriptional regulator |
| SHJG6601 | 7508653 | 7511655 | 3003bp | LuxR-family transcriptional regulator |
| SHJG7225 | 8247468 | 8246479 | 990bp | LuxR-family transcriptional regulator |
| SHJG7255 | 8280945 | 8278309 | 2637bp | LuxR-family transcriptional regulator |
| SHJG7344 | 8379563 | 8376870 | 2694bp | regulatory protein LuxR |
| SHJG8143 | 9337933 | 9335132 | 2802bp | LuxR-family transcriptional regulator |
| SHJG8833 | 10094796 | 10092343 | 2454bp | LuxR-family transcriptional regulator |
| SHJG0534 | 680002 | 681048 | 1047bp | LacI-family transcriptional regulator |
| SHJG0881 | 1060010 | 1061092 | 1083bp | putative LacI-family transcriptional regulator |
| SHJG1117 | 1334942 | 1333971 | 972bp | LacI-family transcriptional regulator |
| SHJG1235 | 1480101 | 1481102 | 1002bp | LacI-family transcriptional regulator |
| SHJG1741 | 2112899 | 2111826 | 1074bp | putative LacI-family transcriptional regulator |
| SHJG1893 | 2295490 | 2296512 | 1023bp | LacI-family transcriptional regulator |
| SHJG2071 | 2529580 | 2528477 | 1104bp | putative LacI-family transcription regulator |
| SHJG2316 | 2793729 | 2794709 | 981bp | LacI-family transcriptional regulator |
| SHJG2528 | 3033593 | 3034642 | 1050bp | LacI-family transcriptional regulator |
| SHJG3079 | 3619758 | 3620777 | 1020bp | LacI-family transcriptional regulator |
| SHJG3412 | 3974184 | 3975311 | 1128bp | LacI-family transcriptional regulator |
| SHJG4246 | 4888101 | 4889159 | 1059bp | LacI-family transcriptional regulator, ribose operon repressor |
| SHJG4254 | 4897600 | 4898646 | 1047bp | LacI-family transcriptional regulator |
| SHJG4297 | 4950222 | 4949167 | 1056bp | LacI-family transcriptional regulator |
| SHJG4302 | 4956039 | 4957088 | 1050bp | putative LacI-family transcriptional regulator |
| SHJG4924 | 5645260 | 5646258 | 999bp | LacI-family regulatory protein |
| SHJG5134 | 5882860 | 5883855 | 996bp | putative LacI-family transcriptional regulator |
| SHJG5851 | 6656643 | 6657674 | 1032bp | LacI/GalR family transcriptional repressor |
| SHJG6780 | 7707846 | 7706833 | 1014bp | LacI-family transcriptional regulator |
| SHJG7590 | 8637367 | 8638344 | 978bp | LacI-family transcription regulator |
| SHJG7612 | 8665501 | 8666577 | 1077bp | LacI-family transcriptional regulator |
| SHJG7706 | 8763971 | 8763057 | 915bp | probable LacI-family transcriptional regulator |
| SHJG7728 | 8788438 | 8789445 | 1008bp | LacI-family transcriptional regulator |
| SHJG7912 | 9002946 | 9003947 | 1002bp | LacI-family transcriptional regulator |
| SHJG8042 | 9190747 | 9191784 | 1038bp | LacI-family transcriptional regulator |
| SHJG8454 | 9682764 | 9683762 | 999bp | sugar-binding LacI-family transcriptional regulator |
| SHJG0102 | 134602 | 134054 | 549bp | IclR-family transcriptional regulator |
| SHJG2083 | 2539857 | 2540624 | 768bp | IclR-family transcriptional regulator |
| SHJG2826 | 3349607 | 3350350 | 744bp | IclR-family transcriptional regulator |
| SHJG3334 | 3892890 | 3893663 | 774bp | IclR-family transcriptional regulator |
| SHJG3910 | 4505964 | 4505191 | 774bp | IclR-family transcriptional regulator |
| SHJG4327 | 4983665 | 4984417 | 753bp | IclR-family transcriptional regulator |
| SHJG4408 | 5064523 | 5063882 | 642bp | putative IclR-family transcriptional regulator |
| SHJG4682 | 5391652 | 5393307 | 1656bp | IclR-family transcriptional regulator |
| SHJG5145 | 5896764 | 5896051 | 714bp | IclR-family transcriptional regulator |
| SHJG6094 | 6941341 | 6942105 | 765bp | IclR-family transcriptional regulator |
| SHJG6663 | 7580584 | 7579868 | 717bp | IclR-family transcriptional regulator |
| SHJG6755 | 7681746 | 7681003 | 744bp | IclR-family transcriptional regulator |
| SHJG7295 | 8323878 | 8323081 | 798bp | IclR-family transcriptional regulator |
| SHJG8730 | 9976477 | 9975725 | 753bp | IclR-family transcriptional regulator |
| SHJG0442 | 577320 | 576349 | 972bp | AsnC-family transcriptional regulator |
| SHJG0510 | 651050 | 651343 | 294bp | AsnC-family transcriptional regulator |
| SHJG2049 | 2505202 | 2504753 | 450bp | AsnC-family transcriptional regulator |
| SHJG2573 | 3082901 | 3083380 | 480bp | AsnC-family transcriptional regulator |
| SHJG2656 | 3169014 | 3169499 | 486bp | AsnC-family transcriptional regulator |
| SHJG2708 | 3216274 | 3216759 | 486bp | AsnC-family transcriptional regulator |
| SHJG2720 | 3227125 | 3227571 | 447bp | AsnC-family transcriptional regulator |
| SHJG2860 | 3392061 | 3391621 | 441bp | AsnC-family transcriptional regulator |
| SHJG2889 | 3423234 | 3422782 | 453bp | AsnC-family transcriptional regulator |
| SHJG4406 | 5062502 | 5062981 | 480bp | AsnC-family transcriptional regulator |
| SHJG4727 | 5445864 | 5445412 | 453bp | AsnC-family transcriptional regulator |
| SHJG5242 | 6004359 | 6003877 | 483bp | AsnC-family transcriptional regulator |
| SHJG5672 | 6483015 | 6483470 | 456bp | AsnC-family transcriptional regulator |
| SHJG6745 | 7669849 | 7669409 | 441bp | AsnC-family transcriptional regulator |
| SHJG0733 | 910464 | 909274 | 1191bp | ROK-family transcriptional regulator |
| SHJG1805 | 2182726 | 2183901 | 1176bp | ROK-family transcriptional regulator |
| SHJG2135 | 2598694 | 2597471 | 1224bp | ROK-family transcriptional regulator |
| SHJG2188 | 2650295 | 2651479 | 1185bp | ROK-family transcriptional regulator |
| SHJG2504 | 3010587 | 3009334 | 1254bp | ROK-family transcriptional regulator |
| SHJG2527 | 3033388 | 3032435 | 954bp | ROK-family transcriptional regulator |
| SHJG2621 | 3134521 | 3135729 | 1209bp | xylose repressor, ROK-family transcriptional regulator |
| SHJG2699 | 3206531 | 3207688 | 1158bp | ROK-family transcriptional regulator |
| SHJG2881 | 3414408 | 3412756 | 1653bp | ROK-family transcriptional regulator |
| SHJG3969 | 4579141 | 4577915 | 1227bp | ROK-family transcriptional regulator |
| SHJG4161 | 4787517 | 4788740 | 1224bp | ROK-family transcriptional regulator |
| SHJG4339 | 4994953 | 4993712 | 1242bp | ROK-family transcriptional regulator |
| SHJG7216 | 8235732 | 8234482 | 1251bp | ROK-family transcriptional regulator |
| SHJG8057 | 9209030 | 9210319 | 1290bp | putative ROK-family transcriptional regulator |
| SHJG0301 | 405999 | 405643 | 357bp | MerR-family transcriptional regulator |
| SHJG0477 | 616016 | 615726 | 291bp | MerR-family transcriptional regulator |
| SHJG1280 | 1539070 | 1538633 | 438bp | MerR-family transcriptional regulator |
| SHJG1681 | 2046537 | 2045638 | 900bp | MerR-family transcriptional regulator |
| SHJG2193 | 2657514 | 2657143 | 372bp | MerR-family transcriptional regulator |
| SHJG4644 | 5351601 | 5352221 | 621bp | MerR-family transcriptional regulator |
| SHJG3871 | 4468653 | 4468198 | 456bp | MerR-family transciptional regulator |
| SHJG3147 | 3692540 | 3692082 | 459bp | MerR-family transcriptional regulator |
| SHJG4581 | 5277537 | 5277178 | 360bp | MerR-family transcriptional regulator |
| SHJG5525 | 6312530 | 6313294 | 765bp | MerR-family transcriptional regulator |
| SHJG6546 | 7453177 | 7454088 | 912bp | MerR-family transcriptional regulator |
| SHJG7627 | 8682582 | 8681818 | 765bp | MerR-family transcriptional regulator |
| SHJG8470 | 9700464 | 9701480 | 1017bp | putative MerR-family transcriptional regulator |
| SHJG1513 | 1794774 | 1795115 | 342bp | ArsR-family transcriptional regulator |
| SHJG1516 | 1797475 | 1797825 | 351bp | ArsR-family transcriptional regulator |
| SHJG1673 | 2036517 | 2035900 | 618bp | putative ArsR-family transcriptional regulator |
| SHJG2730 | 3241331 | 3240978 | 354bp | ArsR-family transcriptional regulator |
| SHJG3153 | 3696112 | 3696825 | 714bp | ArsR-family transcriptional regulator |
| SHJG3524 | 4101049 | 4100462 | 588bp | ArsR-family transcriptional regulator |
| SHJG5031 | 5774256 | 5773849 | 408bp | ArsR-family transcriptional regulator |
| SHJG5299 | 6068752 | 6069102 | 351bp | ArsR-family transcriptional regulator |
| SHJG5551 | 6340165 | 6339062 | 1104bp | putative ArsR-family transcriptional regulator |
| SHJG7136 | 8146639 | 8147007 | 369bp | putative ArsR-family transcriptional regulator |
| SHJG7336 | 8366058 | 8365732 | 327bp | ArsR-family transcriptional regulator |
| SHJG8319 | 9528355 | 9528726 | 372bp | putative ArsR-family transcriptional regulator |
| SHJG0989 | 1173260 | 1172499 | 762bp | DeoR-family transcriptional regulator |
| SHJG1644 | 1969824 | 1969054 | 771bp | DeoR-family transcriptional regulator |
| SHJG2487 | 2994333 | 2995166 | 834bp | DeoR-family transcriptional regulator |
| SHJG2509 | 3014411 | 3015190 | 780bp | DeoR-family transcriptional regulator |
| SHJG3348 | 3905737 | 3906507 | 771bp | DeoR-family transcriptional regulator |
| SHJG3663 | 4255628 | 4256611 | 984bp | DeoR-family transcriptional regulator |
| SHJG4670 | 5378758 | 5377997 | 762bp | DeoR-family transcriptional regulator |
| SHJG4672 | 5380614 | 5381609 | 996bp | DeoR-family transcriptional regulator |
| SHJG5614 | 6410888 | 6411886 | 999bp | DeoR-family transcriptional regulator |
| SHJG6034 | 6879657 | 6880532 | 876bp | DeoR-family transcriptional regulator |
| SHJG6574 | 7484146 | 7485108 | 963bp | DeoR-family transcriptional regulator |
| SHJG0298 | 402035 | 402883 | 849bp | XRE-family transcriptional regulator |
| SHJG0319 | 422736 | 421978 | 759bp | XRE-family transcriptional regulator |
| SHJG0986 | 1170452 | 1169520 | 933bp | XRE-family transcriptional regulator |
| SHJG1166 | 1402407 | 1403036 | 630bp | XRE-family transcriptional regulator |
| SHJG1769 | 2142399 | 2141551 | 849bp | XRE-family transcriptional regulator |
| SHJG5644 | 6444876 | 6445757 | 882bp | XRE-family transcriptional regulator |
| SHJG8472 | 9703193 | 9702306 | 888bp | XRE-family transcriptional regulator |
| SHJG8646 | 9885164 | 9882837 | 2328bp | XRE-family transcriptional regulator |
| SHJG1452 | 1733594 | 1733322 | 273bp | PadR-family transcriptional regulator |
| SHJG2275 | 2749274 | 2748729 | 546bp | putative PadR-like family transcriptional regulator |
| SHJG2296 | 2769440 | 2768847 | 594bp | putative PadR-like family transcriptional regulator |
| SHJG2610 | 3122501 | 3123034 | 534bp | putative PadR-like family transcriptional regulator |
| SHJG2781 | 3289185 | 3288802 | 384bp | putative PadR-family transcriptional regulator |
| SHJG3393 | 3952284 | 3952799 | 516bp | PadR-family transcriptional regulator |
| SHJG5172 | 5922265 | 5922948 | 684bp | PadR-family transcriptional regulator |
| SHJG6133 | 6982599 | 6982072 | 528bp | PadR family transcriptional regulator |
| SHJG6379 | 7252660 | 7251638 | 1023bp | PadR-like family transcriptional regulator |
| SHJG0394 | 527162 | 527560 | 399bp | transcription factor WhiB |
| SHJG1878 | 2275460 | 2275179 | 282bp | WhiB family transcriptional regulator |
| SHJG4500 | 5190044 | 5189781 | 264bp | sporulation regulatory protein WhiB |
| SHJG5481 | 6274706 | 6274834 | 129bp | transcription factor WhiB |
| SHJG5864 | 6669759 | 6669505 | 255bp | WhiB-family transcriptional regulator |
| SHJG6143 | 6994006 | 6994278 | 273bp | WhiB family transcriptional regulator |
| SHJG7350 | 8386488 | 8386300 | 189bp | transcription factor WhiB |
| SHJG7614 | 8667683 | 8667928 | 246bp | WhiB-family transcriptional regulator |
| SHJG2365 | 2845040 | 2842716 | 2325bp | putative regulatory protein AfsR-like |
| SHJG6190 | 7043080 | 7045995 | 2916bp | putative AfsR-like transcriptional regulator |
| SHJG6506 | 7402225 | 7400285 | 1941bp | AfsR-like regulator |
| SHJG7339 | 8369308 | 8372715 | 3408bp | putative AfsR-like transcriptional regulator |
| SHJG7352 | 8388256 | 8387342 | 915bp | putative regulatory protein AfsR-like |
| SHJG0322 | 425044 | 425661 | 618bp | SARP-family transcriptional regulator |
| SHJG1162 | 1398825 | 1396879 | 1947bp | SARP-family transcriptional regulator |
| SHJG1204 | 1442111 | 1442851 | 741bp | SARP-family pathway-specific regulator |
| SHJG0100 | 131768 | 132349 | 582bp | putative Crp/Fnr family transcriptional regulator |
| SHJG5789 | 6605436 | 6604720 | 717bp | Crp/Fnr family transcriptional regulator |
| SHJG7787 | 8855982 | 8856680 | 699bp | DJ-1/PfpI-family transcriptional regulator |
| SHJG8015 | 9164364 | 9163669 | 696bp | DJ-1/PfpI-family transcriptional regulator |
| SHJG0244 | 324645 | 323908 | 738bp | FadR-family transcriptional regulator |
| SHJG8428 | 9652622 | 9653359 | 738bp | putative FadR-family transcriptional regulator |
| SHJG4818 | 5544747 | 5545652 | 906bp | RpiR family transcriptional regulator |
| SHJG1178 | 1412713 | 1413075 | 363bp | HxlR-family transcriptional regulator |
| SHJG7421 | 8454721 | 8454206 | 516bp | SIR2-family transcriptional regulator |
| SHJG1831 | 2214185 | 2215084 | 900bp | SIR2-family transcriptional regulator |
| SHJG6542 | 7449942 | 7450445 | 504bp | AbaA-like regulatory protein |
| SHJG7222 | 8243039 | 8244436 | 1398bp | ADA-like regulatory protein |
| SHJG7419 | 8452209 | 8453675 | 1467bp | ADA-like regulatory protein |
| SHJG6822 | 7763295 | 7763005 | 291bp | BldB regulator |
| SHJG0515 | 656913 | 657317 | 405bp | cell division protein, regulatory protein |
| SHJG2204 | 2669467 | 2669054 | 414bp | cell division protein, regulatory protein |
| SHJG2961 | 3500931 | 3501344 | 414bp | cell division protein, regulatory protein |
| SHJG4629 | 5335185 | 5334919 | 267bp | cell division protein, regulatory protein |
| SHJG7618 | 8672491 | 8672075 | 417bp | cell division protein, regulatory protein |
| SHJG1492 | 1774266 | 1773811 | 456bp | CRP-like regulatory protein |
| SHJG8259 | 9469364 | 9469801 | 438bp | Fe regulatory protein |
| SHJG3097 | 3639990 | 3640754 | 765bp | glycerol operon regulatory protein |
| SHJG4750 | 5467839 | 5467147 | 693bp | iron dependent regulatory protein |
| SHJG3722 | 4315496 | 4316491 | 996bp | maltose operon transcriptional repressor |
| SHJG3748 | 4348128 | 4344871 | 3258bp | multi-domain regulatory protein |
| SHJG4025 | 4636797 | 4637135 | 339bp | nitrogen regulatory protein P-II |
| SHJG6705 | 7628676 | 7629014 | 339bp | nitrogen regulatory protein P-II |
| SHJG0256 | 340155 | 340505 | 351bp | NmrA-like protein |
| SHJG5926 | 6746369 | 6747049 | 681bp | pathway specific regulatory protein |
| SHJG4865 | 5590921 | 5591610 | 690bp | phosphate transport system regulator |
| SHJG1794 | 2169604 | 2170320 | 717bp | possible transcriptional regulator |
| SHJG2036 | 2493374 | 2492421 | 954bp | probable transcriptional regulator |
| SHJG7081 | 8085648 | 8086847 | 1200bp | probable transcriptional repressor protein |
| SHJG0069 | 84643 | 87042 | 2400bp | protein kinase/ transcriptional regulator |
| SHJG1531 | 1814819 | 1815094 | 276bp | putative regulator |
| SHJG2149 | 2613542 | 2613970 | 429bp | putative regulator |
| SHJG2343 | 2819334 | 2818909 | 426bp | putative regulator |
| SHJG3196 | 3745871 | 3744666 | 1206bp | putative regulator |
| SHJG6881 | 7835915 | 7834767 | 1149bp | putative regulator component |
| SHJG6882 | 7836429 | 7835908 | 522bp | putative regulator component |
| SHJG2473 | 2978085 | 2976166 | 1920bp | putative regulator of polyketide synthase expression |
| SHJG0042 | 47316 | 47816 | 501bp | putative regulatory protein |
| SHJG0354 | 481987 | 479627 | 2361bp | putative regulatory protein |
| SHJG0513 | 655589 | 652647 | 2943bp | putative regulatory protein |
| SHJG0755 | 932681 | 929835 | 2847bp | putative regulatory protein |
| SHJG1046 | 1243375 | 1241231 | 2145bp | putative regulatory protein |
| SHJG1217 | 1457441 | 1457557 | 117bp | putative regulatory protein |
| SHJG1938 | 2391020 | 2391685 | 666bp | putative regulatory protein |
| SHJG2096 | 2553117 | 2552599 | 519bp | putative regulatory protein |
| SHJG2104 | 2564769 | 2565203 | 435bp | putative regulatory protein |
| SHJG2111 | 2571584 | 2572042 | 459bp | putative regulatory protein |
| SHJG2456 | 2955938 | 2956276 | 339bp | putative regulatory protein |
| SHJG2639 | 3153475 | 3152903 | 573bp | putative regulatory protein |
| SHJG5333 | 6106926 | 6107348 | 423bp | putative regulatory protein |
| SHJG7776 | 8848933 | 8849742 | 810bp | putative regulatory protein |
| SHJG8176 | 9373510 | 9372764 | 747bp | putative regulatory protein |
| SHJG8276 | 9487760 | 9486477 | 1284bp | putative regulatory protein |
| SHJG0388 | 519412 | 519795 | 384bp | transcription factor |
| SHJG2300 | 2772576 | 2773724 | 1149bp | transcription termination factor Rho |
| SHJG2915 | 3450898 | 3450464 | 435bp | transcription antitermination protein NusB |
| SHJG4045 | 4656087 | 4655071 | 1017bp | heat-inducible transcription repressor |
| SHJG5776 | 6586166 | 6587029 | 864bp | transcription antitermination protein |
| SHJG6070 | 6917331 | 6916834 | 498bp | transcription elongation factor |
| SHJG6438 | 7328633 | 7330675 | 2043bp | transcription termination factor Rho |
| SHJG6794 | 7721120 | 7722106 | 987bp | transcription elongation factor NusA |
| SHJG6919 | 7883229 | 7884095 | 867bp | sporulation transcription factor WhiH |
| SHJG7636 | 8694196 | 8691815 | 2382bp | transcription accessory protein |
| SHJG7324 | 8354241 | 8355428 | 1188bp | putative TylR-like regulatory protein |
| SHJG2913 | 3449541 | 3448960 | 582bp | pyrimidine regulatory protein PyrR |
| SHJG6858 | 7813349 | 7813765 | 417bp | recombination regulator RecX |
| SHJG5604 | 6400724 | 6401500 | 777bp | redox-sensing transcriptional repressor Rex |
| SHJG6901 | 7859270 | 7858491 | 780bp | SOS regulatory protein LexA |
| SHJG3405 | 3967621 | 3966632 | 990bp | sporulation regulatory protein |
| SHJG7325 | 8355492 | 8356520 | 1029bp | streptomycin biosynthesis operon regulator |
| SHJG8657 | 9894809 | 9895747 | 939bp | streptomycin biosynthesis operon regulator |
| SHJG8572 | 9802962 | 9806372 | 3411bp | putative transcriptional activator |
| SHJG0599 | 748342 | 749649 | 1308bp | putative transcriptional regulator |
| SHJG0638 | 803421 | 802597 | 825bp | putative transcriptional regulator |
| SHJG0754 | 929730 | 928738 | 993bp | putative transcriptional regulator |
| SHJG0801 | 979548 | 976651 | 2898bp | putative transcriptional regulator |
| SHJG0813 | 993622 | 993257 | 366bp | putative transcriptional regulator |
| SHJG0814 | 994359 | 993619 | 741bp | putative transcriptional regulator |
| SHJG0936 | 1121864 | 1120986 | 879bp | putative transcriptional regulator |
| SHJG1594 | 1908844 | 1910043 | 1200bp | putative transcriptional regulator |
| SHJG1736 | 2106149 | 2105856 | 294bp | putative transcriptional regulator |
| SHJG1766 | 2139157 | 2139822 | 666bp | putative transcriptional regulator |
| SHJG1799 | 2176307 | 2175591 | 717bp | putative transcriptional regulator |
| SHJG2361 | 2839051 | 2837999 | 1053bp | putative transcriptional regulator |
| SHJG2364 | 2841740 | 2842723 | 984bp | putative transcriptional regulator |
| SHJG2463 | 2963774 | 2963658 | 117bp | putative transcriptional regulator |
| SHJG4356 | 5015333 | 5016421 | 1089bp | putative transcriptional regulator |
| SHJG4549 | 5239738 | 5240592 | 855bp | putative transcriptional regulator |
| SHJG4873 | 5596841 | 5598025 | 1185bp | putative transcriptional regulator |
| SHJG5285 | 6053842 | 6055182 | 1341bp | putative transcriptional regulator |
| SHJG5514 | 6303615 | 6303962 | 348bp | putative transcriptional regulator |
| SHJG6351 | 7228261 | 7228004 | 258bp | putative transcriptional regulator |
| SHJG6678 | 7595917 | 7592753 | 3165bp | putative transcriptional regulator |
| SHJG7199 | 8215767 | 8214841 | 927bp | putative transcriptional regulator |
| SHJG8187 | 9385854 | 9384937 | 918bp | putative transcriptional regulator |
| SHJG8320 | 9529075 | 9530598 | 1524bp | putative transcriptional regulator |
| SHJG8327 | 9536163 | 9536651 | 489bp | putative transcriptional regulator |
| SHJG8362 | 9574512 | 9575474 | 963bp | putative transcriptional regulator |
| SHJG8597 | 9832147 | 9832473 | 327bp | putative transcriptional regulator |
| SHJG8666 | 9904979 | 9904506 | 474bp | putative transcriptional regulator |
| SHJG7677 | 8736987 | 8737553 | 567bp | putative transcriptional regulators |
| SHJG0905 | 1085582 | 1086172 | 591bp | putative transcriptional regulatory protein |
| SHJG4295 | 4946968 | 4948185 | 1218bp | transcriptional activator |
| SHJG4861 | 5587349 | 5586867 | 483bp | transcriptional factor regulator |
| SHJG0213 | 285445 | 284735 | 711bp | transcriptional regulator |
| SHJG0336 | 443622 | 444605 | 984bp | transcriptional regulator |
| SHJG0475 | 615285 | 612463 | 2823bp | transcriptional regulator |
| SHJG0516 | 657413 | 657691 | 279bp | transcriptional regulator |
| SHJG0653 | 820048 | 820335 | 288bp | transcriptional regulator |
| SHJG0684 | 857197 | 858141 | 945bp | transcriptional regulator |
| SHJG0688 | 861677 | 862723 | 1047bp | transcriptional regulator |
| SHJG0992 | 1177781 | 1178803 | 1023bp | transcriptional regulator |
| SHJG1052 | 1253996 | 1252926 | 1071bp | transcriptional regulator |
| SHJG1080 | 1295926 | 1294886 | 1041bp | transcriptional regulator |
| SHJG1082 | 1298248 | 1299075 | 828bp | transcriptional regulator |
| SHJG1601 | 1919937 | 1918969 | 969bp | transcriptional regulator |
| SHJG1700 | 2065748 | 2064894 | 855bp | transcriptional regulator |
| SHJG1748 | 2119648 | 2121546 | 1899bp | transcriptional regulator |
| SHJG1864 | 2257356 | 2256352 | 1005bp | transcriptional regulator |
| SHJG1882 | 2279796 | 2278543 | 1254bp | transcriptional regulator |
| SHJG2279 | 2752371 | 2752009 | 363bp | transcriptional regulator |
| SHJG2330 | 2806473 | 2806075 | 399bp | transcriptional regulator |
| SHJG2625 | 3139850 | 3141157 | 1308bp | transcriptional regulator |
| SHJG2749 | 3257597 | 3258067 | 471bp | transcriptional regulator |
| SHJG2895 | 3428544 | 3427525 | 1020bp | transcriptional regulator |
| SHJG3023 | 3563826 | 3562366 | 1461bp | transcriptional regulator |
| SHJG3030 | 3569687 | 3568347 | 1341bp | transcriptional regulator |
| SHJG3145 | 3691231 | 3690269 | 963bp | transcriptional regulator |
| SHJG3216 | 3765039 | 3764389 | 651bp | transcriptional regulator |
| SHJG3227 | 3777833 | 3779479 | 1647bp | transcriptional regulator |
| SHJG3536 | 4110721 | 4111224 | 504bp | transcriptional regulator |
| SHJG3624 | 4207202 | 4207483 | 282bp | transcriptional regulator |
| SHJG3689 | 4280579 | 4281568 | 990bp | transcriptional regulator |
| SHJG3701 | 4290034 | 4290498 | 465bp | transcriptional regulator |
| SHJG3782 | 4378361 | 4377480 | 882bp | transcriptional regulator |
| SHJG3903 | 4497392 | 4497033 | 360bp | transcriptional regulator |
| SHJG4509 | 5198650 | 5199837 | 1188bp | transcriptional regulator |
| SHJG4514 | 5204979 | 5206346 | 1368bp | transcriptional regulator |
| SHJG4516 | 5207127 | 5208362 | 1236bp | transcriptional regulator |
| SHJG4545 | 5236799 | 5235954 | 846bp | transcriptional regulator |
| SHJG4697 | 5413060 | 5414034 | 975bp | transcriptional regulator |
| SHJG4747 | 5464770 | 5463487 | 1284bp | transcriptional regulator |
| SHJG4965 | 5700168 | 5696770 | 3399bp | transcriptional regulator |
| SHJG5159 | 5907218 | 5907670 | 453bp | transcriptional regulator |
| SHJG5441 | 6225813 | 6225139 | 675bp | transcriptional regulator |
| SHJG5507 | 6293965 | 6297654 | 3690bp | transcriptional regulator |
| SHJG5521 | 6308943 | 6309584 | 642bp | transcriptional regulator |
| SHJG5632 | 6433827 | 6433423 | 405bp | transcriptional regulator |
| SHJG5735 | 6544723 | 6545190 | 468bp | transcriptional regulator |
| SHJG5852 | 6657929 | 6658888 | 960bp | transcriptional regulator |
| SHJG6188 | 7042349 | 7040874 | 1476bp | transcriptional regulator |
| SHJG6439 | 7331044 | 7332177 | 1134bp | transcriptional regulator |
| SHJG6615 | 7527896 | 7526634 | 1263bp | transcriptional regulator |
| SHJG6620 | 7533080 | 7533994 | 915bp | transcriptional regulator |
| SHJG6661 | 7579187 | 7578543 | 645bp | transcriptional regulator |
| SHJG6691 | 7611186 | 7610791 | 396bp | transcriptional regulator |
| SHJG7276 | 8303902 | 8304225 | 324bp | transcriptional regulator |
| SHJG7588 | 8636047 | 8635391 | 657bp | transcriptional regulator |
| SHJG7899 | 8992206 | 8992889 | 684bp | transcriptional regulator |
| SHJG8302 | 9510768 | 9511160 | 393bp | transcriptional regulator |
| SHJG8343 | 9553178 | 9550431 | 2748bp | transcriptional regulator |
| SHJG8465 | 9696651 | 9695533 | 1119bp | transcriptional regulator |
| SHJG8473 | 9703391 | 9703798 | 408bp | transcriptional regulator |
| SHJG8522 | 9751154 | 9750942 | 213bp | transcriptional regulator |
| SHJG8547 | 9776379 | 9777308 | 930bp | transcriptional regulator |
| SHJG8615 | 9849913 | 9849137 | 777bp | transcriptional regulator |
| SHJG8616 | 9850385 | 9849924 | 462bp | transcriptional regulator |
| SHJG8647 | 9885342 | 9886424 | 1083bp | transcriptional regulator |
| SHJG8650 | 9889266 | 9888256 | 1011bp | transcriptional regulator |
| SHJG8656 | 9893941 | 9892829 | 1113bp | transcriptional regulator |
| SHJG8681 | 9925026 | 9924352 | 675bp | transcriptional regulator |
| SHJG0576 | 722327 | 721509 | 819bp | transcriptional regulator protein |
| SHJG2733 | 3243664 | 3243092 | 573bp | transcriptional regulatory protein |
| SHJG3588 | 4172314 | 4171949 | 366bp | transcriptional regulatory protein |
| SHJG8166 | 9363436 | 9364545 | 1110bp | transcriptional repressor |
| SHJG4582 | 5281108 | 5277566 | 3543bp | transcriptional-repair coupling factor |
| SHJG4145 | 4773212 | 4773754 | 543bp | UbiC transcription regulator-associated domain protein |
| SHJG0139 | 189448 | 187148 | 2301bp | regulator |
| SHJG0144 | 202114 | 204957 | 2844bp | regulator |
| SHJG0349 | 473911 | 474378 | 468bp | regulator |
| SHJG0364 | 490563 | 492362 | 1800bp | regulator |
| SHJG0391 | 522566 | 522177 | 390bp | regulator |
| SHJG0452 | 587584 | 587141 | 444bp | regulator |
| SHJG0483 | 622972 | 620717 | 2256bp | regulator |
| SHJG0519 | 661191 | 661805 | 615bp | regulator |
| SHJG0550 | 696353 | 696712 | 360bp | regulator |
| SHJG0803 | 981549 | 982007 | 459bp | regulator |
| SHJG0817 | 1001667 | 998743 | 2925bp | regulator |
| SHJG1292 | 1555069 | 1554647 | 423bp | regulator |
| SHJG1502 | 1785903 | 1785100 | 804bp | regulator |
| SHJG1529 | 1813316 | 1812627 | 690bp | regulator |
| SHJG2435 | 2933377 | 2931983 | 1395bp | regulator |
| SHJG2928 | 3463553 | 3465640 | 2088bp | regulator |
| SHJG3166 | 3712183 | 3711500 | 684bp | regulator |
| SHJG3671 | 4264958 | 4263492 | 1467bp | regulator |
| SHJG4402 | 5057808 | 5058221 | 414bp | regulator |
| SHJG5000 | 5738193 | 5738570 | 378bp | regulator |
| SHJG5144 | 5895951 | 5895514 | 438bp | regulator |
| SHJG5516 | 6304580 | 6304849 | 270bp | regulator |
| SHJG5517 | 6305441 | 6304968 | 474bp | regulator |
| SHJG7043 | 8033583 | 8032771 | 813bp | regulator |
| SHJG4531 | 5222203 | 5221625 | 579bp | regulator of Sig15 |
| SHJG2163 | 2626625 | 2626146 | 480bp | regulator protein |
| SHJG7496 | 8526020 | 8525562 | 459bp | regulator protein |
| SHJG8803 | 10057436 | 10057017 | 420bp | regulator protein |
| SHJG6703 | 7625519 | 7626997 | 1479bp | regulator |
| SHJG0573 | 719265 | 720614 | 1350bp | regulatory protein |
| SHJG0598 | 746645 | 747931 | 1287bp | regulatory protein |
| SHJG0797 | 972788 | 970779 | 2010bp | regulatory protein |
| SHJG0947 | 1129730 | 1130743 | 1014bp | regulatory protein |
| SHJG1420 | 1698061 | 1697831 | 231bp | regulatory protein |
| SHJG1943 | 2393722 | 2396559 | 2838bp | regulatory protein |
| SHJG1945 | 2397181 | 2398884 | 1704bp | regulatory protein |
| SHJG1985 | 2435939 | 2435613 | 327bp | regulatory protein |
| SHJG2004 | 2456502 | 2456951 | 450bp | regulatory protein |
| SHJG2080 | 2538126 | 2537269 | 858bp | regulatory protein |
| SHJG2115 | 2575741 | 2575992 | 252bp | regulatory protein |
| SHJG2206 | 2672787 | 2671291 | 1497bp | regulatory protein |
| SHJG2370 | 2848999 | 2848469 | 531bp | regulatory protein |
| SHJG2675 | 3187730 | 3187275 | 456bp | regulatory protein |
| SHJG3054 | 3595423 | 3594851 | 573bp | regulatory protein |
| SHJG3221 | 3771422 | 3769362 | 2061bp | regulatory protein |
| SHJG3574 | 4155292 | 4156338 | 1047bp | regulatory protein |
| SHJG3704 | 4292223 | 4292798 | 576bp | regulatory protein |
| SHJG3714 | 4302178 | 4302483 | 306bp | regulatory protein |
| SHJG4271 | 4919117 | 4920703 | 1587bp | regulatory protein |
| SHJG4483 | 5163907 | 5164899 | 993bp | regulatory protein |
| SHJG4714 | 5432989 | 5435856 | 2868bp | regulatory protein |
| SHJG4737 | 5454602 | 5454129 | 474bp | regulatory protein |
| SHJG5075 | 5820305 | 5819877 | 429bp | regulatory protein |
| SHJG5101 | 5845815 | 5846327 | 513bp | regulatory protein |
| SHJG5209 | 5969594 | 5968893 | 702bp | regulatory protein |
| SHJG5293 | 6062691 | 6064034 | 1344bp | regulatory protein |
| SHJG5374 | 6149658 | 6150635 | 978bp | regulatory protein |
| SHJG5433 | 6218637 | 6218876 | 240bp | regulatory protein |
| SHJG5681 | 6491790 | 6491308 | 483bp | regulatory protein |
| SHJG6074 | 6919823 | 6919461 | 363bp | regulatory protein |
| SHJG6161 | 7009138 | 7008737 | 402bp | regulatory protein |
| SHJG6198 | 7051409 | 7051837 | 429bp | regulatory protein |
| SHJG6513 | 7409314 | 7408700 | 615bp | regulatory protein |
| SHJG6775 | 7702757 | 7704334 | 1578bp | regulatory protein |
| SHJG6834 | 7783185 | 7780555 | 2631bp | regulatory protein |
| SHJG7035 | 8023319 | 8022480 | 840bp | regulatory protein |
| SHJG7036 | 8023526 | 8024857 | 1332bp | regulatory protein |
| SHJG7040 | 8029328 | 8028876 | 453bp | regulatory protein |
| SHJG7192 | 8208083 | 8206773 | 1311bp | regulatory protein |
| SHJG7230 | 8251227 | 8251670 | 444bp | regulatory protein |
| SHJG7235 | 8257687 | 8256014 | 1674bp | regulatory protein |
| SHJG7377 | 8406453 | 8406785 | 333bp | regulatory protein |
| SHJG7400 | 8432150 | 8433715 | 1566bp | regulatory protein |
| SHJG7602 | 8652950 | 8655514 | 2565bp | regulatory protein |
| SHJG7639 | 8695627 | 8696016 | 390bp | regulatory protein |
| SHJG7660 | 8719615 | 8720244 | 630bp | regulatory protein |
| SHJG7752 | 8815239 | 8814154 | 1086bp | regulatory protein |
| SHJG7753 | 8815334 | 8817058 | 1725bp | regulatory protein |
| SHJG7809 | 8883020 | 8883502 | 483bp | regulatory protein |
| SHJG8131 | 9322442 | 9324889 | 2448bp | regulatory protein |
| SHJG8253 | 9464144 | 9463257 | 888bp | regulatory protein |
| SHJG8405 | 9628293 | 9628775 | 483bp | regulatory protein |
| SHJG8593 | 9828632 | 9828940 | 309bp | regulatory protein |
| SHJG8864 | 10136865 | 10136398 | 468bp | regulatory protein |
| SHJG1704 | 2071211 | 2068422 | 2790bp | response regulator |
| SHJG7256 | 8282163 | 8281333 | 831bp | response regulator |
| SHJG2082 | 2539673 | 2539236 | 438bp | response regulator receiver protein |
| SHJG0511 | 651405 | 651770 | 366bp | response regulator receiver protein |
| SHJG2404 | 2893993 | 2894631 | 639bp | response regulator receiver protein |

**⑶ Sigma / anti-sigma / anti-anti-sigma factors**

| **ID** | **Start** | **End** | **Size** | **Function** |
| --- | --- | --- | --- | --- |
| SHJG0145 | 205418 | 205753 | 336bp | RNA polymerase ECF-subfamily sigma factor |
| SHJG0180 | 243129 | 242572 | 558bp | RNA polymerase ECF-subfamily sigma-24 subunit |
| SHJG0450 | 585184 | 584204 | 981bp | RNA polymerase ECF-subfamily sigma factor |
| SHJG0714 | 885725 | 886870 | 1146bp | RNA polymerase ECF-subfamily sigma factor |
| SHJG0741 | 918434 | 918598 | 165bp | RNA polymerase ECF-subfamily sigma factor |
| SHJG0774 | 952004 | 951486 | 519bp | putative RNA polymerase sigma factor |
| SHJG1267 | 1527457 | 1527975 | 519bp | putative RNA polymerase sigma factor |
| SHJG1682 | 2047415 | 2046822 | 594bp | RNA polymerase ECF-subfamily sigma factor |
| SHJG2092 | 2548279 | 2549085 | 807bp | RNA polymerase secondary sigma factor |
| SHJG2108 | 2570270 | 2569404 | 867bp | putative RNA polymerase sigma factor |
| SHJG2162 | 2625195 | 2626085 | 891bp | putative RNA polymerase sigma factor |
| SHJG2176 | 2638264 | 2637551 | 714bp | RNA polymerase ECF-subfamily sigma factor |
| SHJG2329 | 2805909 | 2803951 | 1959bp | putative RNA polymerase ECF-subfamily sigma factor |
| SHJG2341 | 2815746 | 2816681 | 936bp | putative RNA polymerase principal sigma factor HrdC |
| SHJG2372 | 2850861 | 2850451 | 411bp | RNA polymerase sigma factor SigL |
| SHJG2701 | 3209845 | 3208961 | 885bp | RNA polymerase ECF-subfamily sigma factor |
| SHJG2986 | 3526575 | 3526069 | 507bp | RNA polymerase sigma factor |
| SHJG3007 | 3548022 | 3547435 | 588bp | RNA polymerase ECF-subfamily sigma-24 subunit |
| SHJG3173 | 3717072 | 3716530 | 543bp | RNA polymerase sigma factor SigK |
| SHJG3800 | 4396712 | 4396173 | 540bp | putative RNA polymerase ECF-subfamily sigma factor |
| SHJG3942 | 4548595 | 4547471 | 1125bp | RNA polymerase sigma factor |
| SHJG4141 | 4768517 | 4767966 | 552bp | RNA polymerase ECF-subfamily sigma factor |
| SHJG4152 | 4778885 | 4779493 | 609bp | RNA polymerase ECF-subfamily sigma factor |
| SHJG4427 | 5087900 | 5087412 | 489bp | RNA polymerase sigma factor SigL |
| SHJG4533 | 5223261 | 5224157 | 897bp | RNA polymerase sigma factor |
| SHJG4674 | 5383808 | 5382810 | 999bp | RNA polymerase principal sigma factor HrdD |
| SHJG4716 | 5436073 | 5436264 | 192bp | sigma-like protein |
| SHJG4740 | 5456594 | 5457151 | 558bp | RNA polymerase ECF-subfamily sigma factor |
| SHJG4792 | 5516403 | 5515384 | 1020bp | putative RNA polymerase sigma factor |
| SHJG4837 | 5566843 | 5565911 | 933bp | RNA polymerase ECF-subfamily sigma factor |
| SHJG5051 | 5789415 | 5790146 | 732bp | RNA polymerase sigma factor |
| SHJG5052 | 5790484 | 5791425 | 942bp | RNA polymerase sigma factor |
| SHJG5093 | 5839156 | 5838599 | 558bp | putative RNA polymerase ECF-subfamily sigma factor |
| SHJG5179 | 5933904 | 5934653 | 750bp | RNA polymerase sigma factor SigM |
| SHJG5311 | 6084383 | 6085327 | 945bp | putative RNA polymerase ECF-subfamily sigma factor |
| SHJG5332 | 6106697 | 6105852 | 846bp | RNA polymerase sigma factor SigB |
| SHJG5338 | 6109423 | 6109920 | 498bp | putative RNA polymerase ECF-subfamily sigma factor |
| SHJG5412 | 6192793 | 6193302 | 510bp | RNA polymerase ECF-subfamily sigma factor |
| SHJG5573 | 6361762 | 6361223 | 540bp | RNA polymerase ECF-subfamily sigma factor |
| SHJG5601 | 6398838 | 6398059 | 780bp | RNA polymerase ECF-subfamily sigma factor |
| SHJG5854 | 6659402 | 6660658 | 1257bp | RNA polymerase ECF-subfamily sigma factor |
| SHJG5866 | 6671702 | 6672289 | 588bp | RNA polymerase sigma factor SigD |
| SHJG5987 | 6827127 | 6827747 | 621bp | RNA polymerase ECF-subfamily sigma factor |
| SHJG5989 | 6829463 | 6830125 | 663bp | RNA polymerase ECF-subfamily sigma factor |
| SHJG6011 | 6856000 | 6854987 | 1014bp | RNA polymerase factor sigma-70 |
| SHJG6022 | 6865653 | 6866387 | 735bp | RNA polymerase ECF-subfamily sigma factor |
| SHJG6065 | 6912607 | 6912128 | 480bp | RNA polymerase ECF-subfamily sigma factor |
| SHJG6247 | 7108602 | 7109276 | 675bp | RNA polymerase sigma factor SigE |
| SHJG6329 | 7202000 | 7202695 | 696bp | RNA polymerase sigma factor RpoE |
| SHJG6354 | 7231226 | 7230228 | 999bp | RNA polymerase sigma factor SigH |
| SHJG6537 | 7443584 | 7443078 | 507bp | RNA polymerase ECF-subfamily sigma factor |
| SHJG6725 | 7648252 | 7649028 | 777bp | RNA polymerase sigma factor WhiG |
| SHJG6920 | 7884531 | 7886072 | 1542bp | RNA polymerase sigma factor RpoD |
| SHJG7065 | 8059316 | 8060254 | 939bp | RNA polymerase ECF-subfamily sigma factor |
| SHJG7756 | 8821583 | 8822158 | 576bp | RNA polymerase sigma factor |
| SHJG7864 | 8954650 | 8954102 | 549bp | RNA polymerase sigma factor SigL |
| SHJG7994 | 9136101 | 9135133 | 969bp | RNA polymerase ECF-subfamily sigma factor |
| SHJG8013 | 9162937 | 9162275 | 663bp | RNA polymerase ECF-subfamily sigma-24 subunit |
| SHJG8095 | 9243319 | 9243867 | 549bp | RNA polymerase ECF-subfamily sigma factor |
| SHJG8155 | 9353276 | 9353995 | 720bp | putative RNA polymerase ECF-subfamily sigma factor |
| SHJG8323 | 9532556 | 9533173 | 618bp | RNA polymerase ECF-subfamily sigma factor |
| SHJG8564 | 9795934 | 9795059 | 876bp | putative RNA polymerase ECF-subfamily sigma factor |
| SHJG3923 | 4525608 | 4524646 | 963bp | anti-sigma factor |
| SHJG5462 | 6251513 | 6251947 | 435bp | anti-sigma factor |
| SHJG6330 | 7202692 | 7203003 | 312bp | anti-sigma factor |
| SHJG6355 | 7231771 | 7231355 | 417bp | anti-sigma factor |
| SHJG8251 | 9462880 | 9462434 | 447bp | anti-sigma factor |
| SHJG0976 | 1160262 | 1160627 | 366bp | anti-sigma factor antagonist |
| SHJG1797 | 2174165 | 2173794 | 372bp | anti-sigma factor antagonist |
| SHJG4532 | 5223014 | 5222637 | 378bp | anti-sigma factor antagonist |
| SHJG4739 | 5456253 | 5455879 | 375bp | anti-sigma factor antagonist |
| SHJG5461 | 6250990 | 6251331 | 342bp | anti-sigma factor antagonist |
| SHJG6464 | 7354477 | 7354800 | 324bp | anti-sigma factor antagonist |
| SHJG7376 | 8406153 | 8405866 | 288bp | anti-sigma factor antagonist |
| SHJG7493 | 8523162 | 8522776 | 387bp | anti-sigma-factor antagonist |
| SHJG8074 | 9223398 | 9223018 | 381bp | anti-sigma factor antagonist |
| SHJG8252 | 9463260 | 9462880 | 381bp | anti-sigma factor antagonist |
| SHJG8317 | 9526498 | 9526094 | 405bp | anti-sigma factor antagonist |

**⑷ Serine / threonine protein kinases**

| **ID** | **Start** | **End** | **Size** | **Function** |
| --- | --- | --- | --- | --- |
| SHJG0034 | 40763 | 41374 | 612bp | putative serine/threonine protein kinase |
| SHJG1557 | 1856677 | 1860291 | 3615bp | serine/threonine protein kinase |
| SHJG2147 | 2610821 | 2612401 | 1581bp | putative serine/threonine protein kinase |
| SHJG2972 | 3509759 | 3511234 | 1476bp | serine/threonine protein kinase |
| SHJG3594 | 4176489 | 4178456 | 1968bp | eukaryotic-type serine/threonine protein kinase |
| SHJG3625 | 4210049 | 4207500 | 2550bp | putative serine/threonine protein kinase |
| SHJG3921 | 4519506 | 4523432 | 3927bp | serine/threonine protein kinase |
| SHJG4173 | 4800272 | 4802206 | 1935bp | serine/threonine protein kinase |
| SHJG4443 | 5106290 | 5105064 | 1227bp | putative serine/threonine protein kinase |
| SHJG4444 | 5108126 | 5106402 | 1725bp | serine/threonine protein kinase |
| SHJG4573 | 5269428 | 5267914 | 1515bp | serine/threonine protein kinase |
| SHJG4723 | 5443654 | 5441291 | 2364bp | serine/threonine protein kinase (fragment) |
| SHJG5207 | 5967852 | 5966482 | 1371bp | serine/threonine protein kinase |
| SHJG5218 | 5978061 | 5976067 | 1995bp | serine/threonine protein kinase |
| SHJG5252 | 6015970 | 6014330 | 1641bp | serine/threonine protein kinase |
| SHJG5253 | 6016389 | 6017951 | 1563bp | serine/threonine protein kinase |
| SHJG5346 | 6118277 | 6119536 | 1260bp | putative serine/threonine protein kinase |
| SHJG5350 | 6122274 | 6123704 | 1431bp | serine/threonine protein kinase |
| SHJG5402 | 6180628 | 6182463 | 1836bp | serine-threonine protein kinase |
| SHJG5584 | 6376781 | 6374571 | 2211bp | serine/threonine protein kinase |
| SHJG5685 | 6493714 | 6495327 | 1614bp | serine-threonine protein kinase |
| SHJG5872 | 6679202 | 6681163 | 1962bp | serine/threonine protein kinase |
| SHJG5873 | 6681327 | 6684056 | 2730bp | serine/threonine protein kinase |
| SHJG5875 | 6686156 | 6687868 | 1713bp | serine/threonine protein kinase |
| SHJG5878 | 6688403 | 6689341 | 939bp | serine/threonine protein kinase |
| SHJG6816 | 7751530 | 7752480 | 951bp | putative serine/threonine protein kinase |
| SHJG7247 | 8269694 | 8271961 | 2268bp | serine/threonine protein kinase |
| SHJG7801 | 8870579 | 8871397 | 819bp | putative serine/threonine protein kinase |
| SHJG7802 | 8871579 | 8872841 | 1263bp | serine/threonine protein kinase |
| SHJG7817 | 8894058 | 8892046 | 2013bp | serine/threonine protein kinase |
| SHJG8094 | 9243271 | 9241880 | 1392bp | serine/threonine protein kinase |
| SHJG8149 | 9347833 | 9346580 | 1254bp | serine/threonine protein kinase |
| SHJG8370 | 9583859 | 9581664 | 2196bp | putative serine/threonine protein kinase |
| SHJG8371 | 9585997 | 9583859 | 2139bp | putative serine/threonine protein kinase |
| SHJG8846 | 10110633 | 10114247 | 3615bp | serine/threonine protein kinase |
| pSHJG1.181 | 157328 | 158452 | 1125bp | serine/threonine protein kinase |
