# Supplementary material for: Genomic and transcriptomic insights into the thermo-regulated biosynthesis of validamycin in Streptomyces hygroscopicus 5008
Source: BMC Genomics. 2012 Jul 24;13:337. doi: 10.1186/1471-2164-13-337 (PMC3424136; doi:10.1186/1471-2164-13-337)
Supplement: Additional file 10 — Table S7. Alignment between ArpA/AfsA from S. griseus and S. hygroscopicus 5008 genome with BLASTP program (e value = 1e-10). [file 1471-2164-13-337-S10.docx]

**Additional file 12: Table S7 Alignment between ArpA / AfsA from *S. griseus* and *S. hygroscopicus* 5008 genome with BLASTP program (e value=1e-10)**

| **Query** | **Subject** | **Function** | **Identity (%)** |
| --- | --- | --- | --- |
| ArpA | SHJG0887 | putative TetR-family transcriptional regulator | 32.12 |
|  |  |  |  |
| ArpA | SHJG4003 | gamma-butyrolactone receptor protein | 34.97 |
| AfsA | SHJG4004 | hypothetical gamma-butyrolactone biosynthesis enzyme | 33.91 |
|  |  |  |  |
| ArpA | SHJG7144 | A-factor receptor homolog | 34.92 |
|  |  |  |  |
| ArpA | SHJG7318 | TetR-family transcriptional regulator | 30.35 |
| AfsA | SHJG7319 | gamma-butyrolactone biosynthesis protein | 35.71 |
|  |  |  |  |
| ArpA | SHJG8325 | putative gamma-butyrolactone-binding protein | 41.12 |
|  |  |  |  |
| AfsA | SHJG8650 | transcriptional regulator | 33.9 |
| ArpA | SHJG8651 | butyrolactone receptor | 36.63 |
