# Supplementary material for: Genomic and transcriptomic insights into the thermo-regulated biosynthesis of validamycin in Streptomyces hygroscopicus 5008
Source: BMC Genomics. 2012 Jul 24;13:337. doi: 10.1186/1471-2164-13-337 (PMC3424136; doi:10.1186/1471-2164-13-337)

**Additional file 4: Figure S4 Constructions of other knock-out mutants of strain 5008. (A)** Construction of mutant XH3 with the deletion of validamycin gene cluster; **(B)** Construction and PCR confirmation of △*SHJG4152* (RNA polymerase ECF-subfamily sigma factor) mutant JG34; **(C)** Construction and PCR confirmation of △*SHJG4359* (putative heat shock protein) mutant JG35; **(D)** Construction and PCR confirmation of △*SHJG8393* (heat shock protein Hsp90) mutant JG36; **(E)** VAL-A production in strain 5008 and mutants JG34, JG35, JG36 cultivated at 30°C or 37°C. Mean values of three independent experiments with SD are indicated by error bars.


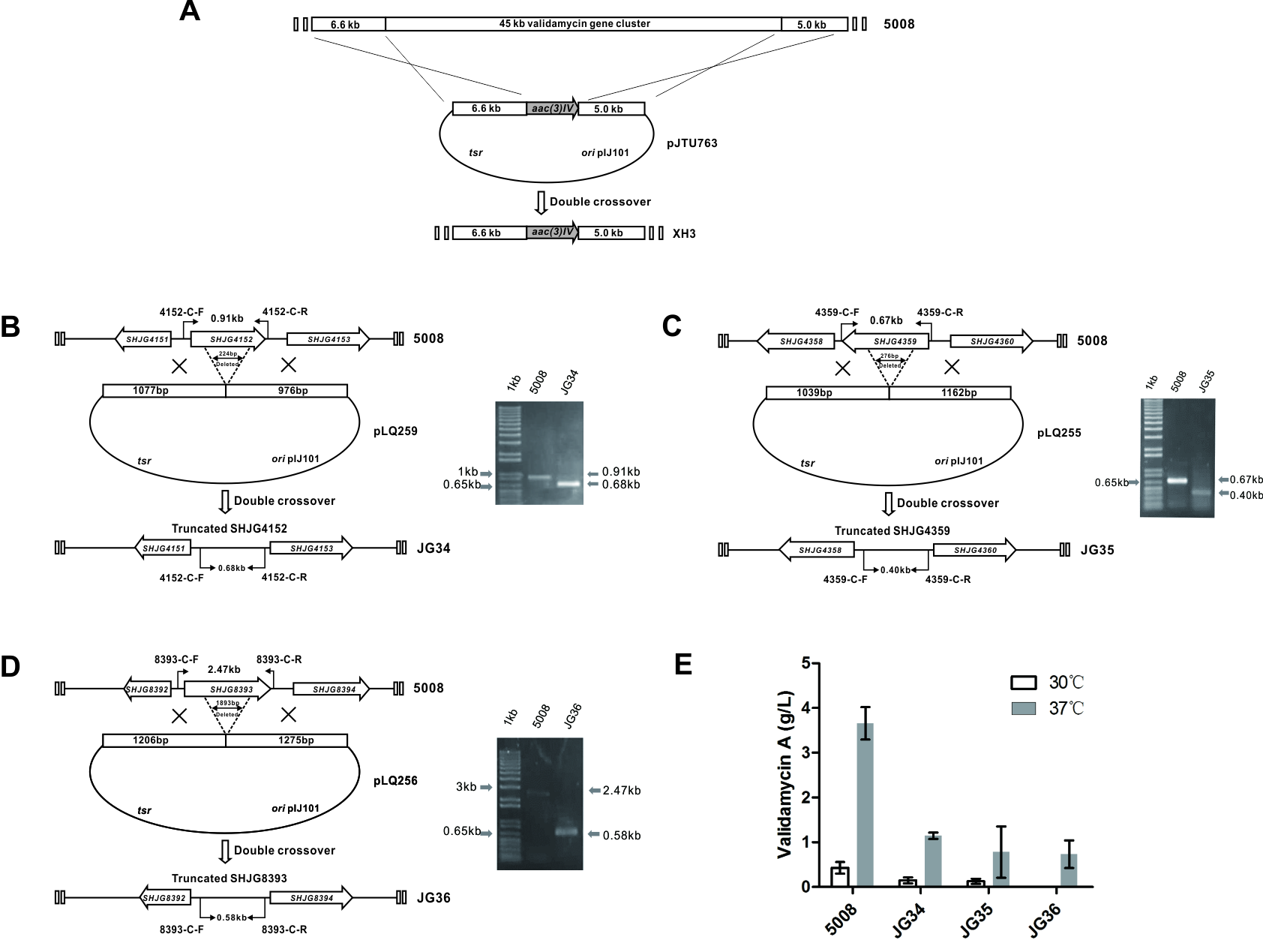

Supplement: Additional file 12 — Figure S4. Constructions of other knock-out mutants of S. hygroscopicus 5008. [file 1471-2164-13-337-S12.docx]
