# Supplementary material for: Genomic and transcriptomic insights into the thermo-regulated biosynthesis of validamycin in Streptomyces hygroscopicus 5008
Source: BMC Genomics. 2012 Jul 24;13:337. doi: 10.1186/1471-2164-13-337 (PMC3424136; doi:10.1186/1471-2164-13-337)
Supplement: Additional file 13 — Method SI. Constructions of other knock-out mutants of S. hygroscopicus 5008 described in this study. [file 1471-2164-13-337-S13.docx]

**Additional file 5: Method SI Constructions of other knock-out mutants described in this study**

**Construction of mutant XH3 with the deletion of validamycin gene cluster**

Two segments of 6.6-kb EcoRI/BamHI and 5.0-kb EcoRI/EcoRI respectively flanking the left and right of the 45-kb validamycin gene cluster, and a BamHI/EcoRI segment carrying the 1.4-kb *aac(3)IV* cassette were ligated into EcoRI-digested pIJ2925[1], a pUC18 derivative with a multi-cloning site flanked by BglII, to generate pJTU762. A BglII segment from pJTU762 was ligated into BamHI-digested pHZ1358[2], an *E. coli*-*Streptomyces* shuttle vector, to generate pJTU763. Then, pJTU763 was used for mediating gene placement to obtain the mutant XH3 with validamycin gene cluster deleted. Desired mutant was confirmed by PCR amplification and sequencing of specific PCR products.

**Inactivation of the ECF sigma factor gene *SHJG4152***

Two segments of 1.08-kb HindIII/EcoRI and 976-bp EcoRI/BamHI from strain 5008 were amplified using two primer pairs 4152-L-F/4152-L-R (5'-ATATGGATCCAGTAGAGCTGGACGAGCA-3'; 5'-ATATGAATTCTGACGAACGTGTCCTGGA-3') and 4152-R-F/4152-R-R (5'-ATATGAATTCGCGCTCGACCGGATCTCC-3'; 5'-ATATAAGCTTGCCATCGGTGCCAGGACG-3'), and ligated into HindIII/BamHI-digested pJTU1278[3] to obtain pLQ259. Then, pLQ259 was introduced into strain 5008 by intergeneric conjugation, and single-crossover exconjugants were screened by thiostrepton resistance (Tsr^R^). Subsequently, marker-free deletion mutant JG34 was selected from the initial Tsr^R^ exconjugants after several rounds of nonselective growth, and confirmed by PCR amplification using the primer pairs 4152-C-F (5'-GGGTGATCCGGGTGATCC-3') and 4152-C-R (5'-CACCGCTTCCTCCGATAC-3'). Instead of a 0.91-kb fragment from the wild-type, the PCR product from the mutant JG34 was 0.68 kb.

**Inactivation of putative heat shock protein gene *SHJG4359***

Two segments of 1.04-kb EcoRI/BamHI and 1.16-kb BamHI/HindIII from strain 5008 were amplified using two primer pairs 4152-L-F/4152-L-R (5'-AATGAATTCGCTGGTGTAACGCATGAC-3'; 5'-AATGGATCCCGACCGCAAGGAGATCAG-3') and 4152-R-F/4152-R-R (5'-AATGGATCCAGTGGACCAGGAACTCTTC-3'; 5'-AATAAGCTTATGATCCGCAGGGACTTC-3'), and ligated into EcoRI /HindIII-digested pJTU1278 to obtain pLQ255. Then, pLQ255 was introduced into strain 5008 by intergeneric conjugation, and single-crossover exconjugants were screened by thiostrepton resistance (Tsr^R^). Subsequently, marker-free deletion mutant JG35 was selected from the initial Tsr^R^ exconjugants after several rounds of nonselective growth, and confirmed by PCR amplification using the primer pairs 4359-C-F (5'-GATGCCTAGTACGCCTGG-3') and 4359-C-R (5'-AAGGAGTGACCCGTGATG-3'). Instead of a 0.67-kb fragment from the wild-type, the PCR product from the mutant JG35 was 0.40 kb.

**Inactivation of the heat shock protein gene *SHJG8393***

Two segments of 1.21-kb HindIII/EcoRI and 1.28-kb EcoRI/KpnI from strain 5008 were respectively amplified using two primer pairs 8393-L-F/8393-L-R (5'-AATAAGCTTTCGTAGGTGATGACACATTC-3'; 5'-AATGAATTCCGAGTAGACCGAGTGGAT-3') and 8393-R-F/8393-R-R (5'- AATGAATTCGGTTCGTGAAGCTGATGG-3'; 5'- AATGGTACCGCTGTAGTTGACGGTCTC-3'), and ligated into HindIII/KpnI-digested pJTU1278 to obtain pLQ256. Then, pLQ256 was introduced into strain 5008 by intergeneric conjugation, and single-crossover exconjugants were screened by thiostrepton resistance (Tsr^R^). Subsequently, marker-free deletion mutant JG36 was selected from the initial Tsr^R^ exconjugants after several rounds of nonselective growth, and confirmed by PCR amplification using the primer pairs 8393-C-F (5'-ACCGTGCTCATCTCTGTA-3') and 8393-C-R (5'-GTAAGTCTCGTGCGTCTC-3'). Instead of a 2.47-kb fragment from the wild-type, the PCR product from the mutant JG36 was 0.58 kb.

1. Janssen GR, Bibb MJ: Derivatives of pUC18 that have BglII sites flanking a modified multiple cloning site and that retain the ability to identify recombinant clones by visual screening of *Escherichia coli* colonies. *Gene* 1993, 124(1):133-134.

2. Sun Y, Zhou X, Liu J, Bao K, Zhang G, Tu G, Kieser T, Deng Z: '*Streptomyces nanchangensis*', a producer of the insecticidal polyether antibiotic nanchangmycin and the antiparasitic macrolide meilingmycin, contains multiple polyketide gene clusters. *Microbiology* 2002, 148(Pt 2):361-371.

3. He Y, Wang Z, Bai L, Liang J, Zhou X, Deng Z: Two pHZ1358-derivative vectors for efficient gene knockout in streptomyces. *J Microbiol Biotechnol* 2010, 20(4):678-682.
