# Supplementary material for: Genomic and transcriptomic insights into the thermo-regulated biosynthesis of validamycin in Streptomyces hygroscopicus 5008
Source: BMC Genomics. 2012 Jul 24;13:337. doi: 10.1186/1471-2164-13-337 (PMC3424136; doi:10.1186/1471-2164-13-337)
Supplement: Additional file 14 — Table S9. Quality-control parameters of DNA microarray data set in S. hygroscopicus 5008 and S. avermitilis NRRL8165. [file 1471-2164-13-337-S14.docx]

**Additional file 14: Table S9 Quality-Control Parameters of DNA Microarray Data Set in *S. hygroscopicus* 5008 and *S. avermitilis* NRRL8165**

| **Samples /Cy3** | **Coefficient of variation (%)** | **Detected rate (%)** |
| --- | --- | --- |
| 5008-30^◦^C -2 | 14.05 | 99.62 |
| 5008-30^◦^C -5 | 14.14 | 98.36 |
| 5008-30^◦^C -5 | 13.50 | 99.75 |
| 5008-30^◦^C -6 | 10.77 | 99.75 |
| 5008-37^◦^C -1 | 13.05 | 98.00 |
| 5008-37^◦^C -4 | 13.04 | 98.87 |
| 5008-37^◦^C -4 | 12.10 | 98.42 |
| 5008-37^◦^C -6 | 13.89 | 99.34 |
| NRRL8165-30^◦^C -2 | 6.64 | 99.31 |
| NRRL8165-30^◦^C -4 | 6.30 | 99.61 |
| NRRL8165-30^◦^C -6 | 8.11 | 99.58 |
| NRRL8165-30^◦^C -6 | 6.47 | 99.44 |
| NRRL8165-37^◦^C -2 | 7.29 | 99.47 |
| NRRL8165-37^◦^C -4 | 4.60 | 98.60 |
| NRRL8165-37℃-6 | 6.73 | 99.48 |
| NRRL8165-37^◦^C -6 | 6.86 | 99.54 |
