# Supplementary material for: Genomic and transcriptomic insights into the thermo-regulated biosynthesis of validamycin in Streptomyces hygroscopicus 5008
Source: BMC Genomics. 2012 Jul 24;13:337. doi: 10.1186/1471-2164-13-337 (PMC3424136; doi:10.1186/1471-2164-13-337)
Supplement: Additional file 15 — Table S10. Quantitative RT-PCR analysis of selected genes in S. hygroscopicus 5008. [file 1471-2164-13-337-S15.docx]

**Additional file 15: Table S10 qRT-PCR analysis of selected genes in *S. hygroscopicus* 5008**

| **Gene** | **Description** | **qRT-PCR** | | **DNA Microarray** | | |
| --- | --- | --- | --- | --- | --- | --- |
|  |  | **Regulation ^a^** | **Fold change ^b^** | **Regulation ^a^** | **Fold change ^b^** | ***p* value** |
| SHJG0322 | SARP-family transcriptional regulator | up | 63.22 | up | 127.35 | 8.3E-08 |
| SHJG2200 | asparagine synthase | up | 13.58 | up | 6.82 | 0.03194 |
| SHJG3007 | RNA polymerase ECF subfamily sigma | up | 4.44 | up | 1.34 | 0.01142 |
| SHJG4290 | phosphoesterase | down | 30.18 | down | 4.02 | 0.00169 |
| SHJG7337 | putative MFS sugar transporter protein | down | 489.29 | down | 194.80 | 0.00623 |
| SHJG7755 | protease | down | 6.50 | down | 3.49 | 0.00091 |
| SHJG8009 | alkaline phosphatase | down | 19.01 | down | 4.32 | 0.01109 |
| SHJG8667 | MFS permease | down | 14.91 | down | 79.59 | 0.00011 |

**^a^** Words represent the trends of the transcriptional changes at 37^◦^C relative to at 30^◦^C

**^b^** Numbers represent fold changes of gene transcription at 37^◦^C relative to at 30^◦^C
