# Supplementary material for: Genomic and transcriptomic insights into the thermo-regulated biosynthesis of validamycin in Streptomyces hygroscopicus 5008
Source: BMC Genomics. 2012 Jul 24;13:337. doi: 10.1186/1471-2164-13-337 (PMC3424136; doi:10.1186/1471-2164-13-337)
Supplement: Additional file 16 — Table S11. Sequences of primer pairs for qRT-PCR assay. [file 1471-2164-13-337-S16.docx]

**Additional file 16: Table S11 Sequences of primer pairs for quantitative RT-PCR (qRT-PCR) assay**

| **Name** | **Sequence (5′-3′)** |
| --- | --- |
| SHJG0275 | Forward: CAGGTGGCTGCCTTGATG  Reverse: AGTGCGCGTGCGATGTT |
| SHJG0276 | Forward: CATACCCACGACCCTGAT  Reverse: CCAACCGATTCTTGTGCT |
| SHJG0322 | Forward: AAGCCCGGATACGGCTGAG  Reverse: GGCGGCGAAGGTGGTTGT |
| SHJG2200 | Forward: CACCGACCACCAGGACA  Reverse: AAGAGCAGCAGCAGGGAG |
| SHJG3007 | Forward: CCGGCATCTACGGCATGA  Reverse: CGGAGAAGCGACGGTGGT |
| SHJG4290 | Forward: ACCACTGCGCCTACTGCA  Reverse: CCTTGCCCGCCTTGAT |
| SHJG7337 | Forward: ACATCGGCGACGACCTG  Reverse: ACGGCGTAGACGGTGAGTG |
| SHJG7755 | Forward: GCCGTCCTGGAGTCGGAGTT  Reverse: TGTCGCTGCCGTTGTTGC |
| SHJG8009 | Forward: GCACGAAGGGCAACCTG  Reverse: GGGCGCTGCTGGAACAT |
| SHJG8667 | Forward: CCACCACCTCCGTCTTCTCC  Reverse: TCCGTTTCGCTCCTCTGCTT |
